# The effect of experimental hybridization on cognition and brain anatomy: Limited phenotypic variation and transgression in Poeciliidae

**DOI:** 10.1111/evo.14644

**Published:** 2022-10-10

**Authors:** Catarina Vila‐Pouca, Hannah De Waele, Alexander Kotrschal

**Affiliations:** ^1^ Behavioural Ecology Group Wageningen University & Research Wageningen 6700 HB The Netherlands

**Keywords:** Brain morphology, cognitive flexibility, guppy, learning, morphospace, transgressive segregation

## Abstract

Hybridization can promote phenotypic variation and often produces trait combinations distinct from the parental species. This increase in available variation can lead to the manifestation of functional novelty when new phenotypes bear adaptive value under the environmental conditions in which they occur. Although the role of hybridization as a driver of variation and novelty in traits linked to fitness is well recognized, it remains largely unknown whether hybridization can fuel behavioral novelty by promoting phenotypic variation in brain morphology and/or cognitive traits. To address this question, we investigated the effect of hybridization on brain anatomy, learning ability, and cognitive flexibility in first‐ and second‐generation hybrids of two closely related fish species (*Poecilia reticulata* and *Poecilia wingei*). Overall, we found that F1 and F2 hybrids showed intermediate brain morphology and cognitive traits compared to parental groups. Moreover, as phenotypic dispersion and transgression were low for both brain and cognitive traits, we suggest that hybridization is not a strong driver of brain anatomical and cognitive diversification in these Poeciliidae. To determine the generality of this conclusion, hybridization experiments with cognitive tests need to be repeated in other families.

In the last decade, studies on phenotypic diversification have established the role of hybridization as a driver of trait variation that often leads to ecological and evolutionary innovations (Rieseberg et al. 2003a; Mallet 2007; Stelkens et al. 2009; Seehausen 2013). Through recombination of parental alleles, hybridization can generate high levels of heritable variation in hybrids, including individuals that express new trait combinations that exceed the range of both parental species (Lewontin and Birch 1966; Dittrich‐Reed and Fitzpatrick 2013). When novel phenotypes bear adaptive value, hybrids may persist and influence the evolutionary trajectories of parental species, for example, through novel adaptations or colonization of underused niches (Mallet 2007; Seehausen 2013).

Novel or transgressive phenotypes are commonly defined as traits or combinations of traits that fall outside the range of variation of parental species (Rieseberg et al. 2003b; Bell and Travis 2005; Stelkens et al. 2009). Transgressive phenotypes are common in both plants and animals and have so far been demonstrated for several fitness‐related traits, including morphology (skull morphology in fish, Stelkens et al. 2009; wing morphology in butterflies, Mérot et al. 2020), physiology (temperature tolerance in copepods, Pereira et al. 2014), life history (number and size of offspring in snails, Facon et al. 2008), and behavioral traits (mating behavior in fruit flies, Ranganath and Aruna 2003; foraging behavior in fish, Selz and Seehausen 2019; Feller et al. 2020). Different mechanisms have been proposed to explain how recombination of parental genomes can result in novel traits (Rieseberg et al. 2003b; Bell and Travis 2005; Stelkens et al. 2009; Thompson et al. 2021). Extreme hybrid phenotypes can arise in first‐generation (F1) hybrids, a phenomenon usually referred to as heterosis, through dominance of some loci contributing to variation in the same trait or epistatic interactions of alleles at different loci, among other mechanisms (Stelkens et al. 2009; Mérot et al. 2020; Thompson et al. 2021). More often, however, increased phenotypic variation and extreme phenotypes occur in subsequent hybrid generations. Possible sources of transgression include dominance/overdominance of some loci and epistasis, as in F1 hybrids, but it may also arise from the action of complementary genes between quantitative trait loci that carry alleles of opposing effects. When alleles have complementary additive effects, F1 hybrids can only express intermediate phenotypes but effects may sum in the F2 and later generations and lead to increased phenotypic variance and transgression, even if the mean hybrid phenotype is intermediate between the parentals (Rieseberg et al. 2003b; Thompson et al. 2021).

Although not classically considered a transgressive effect from a genetic point of view, hybrid novelty may also be observed when hybrid traits fall between disjunct parental ranges or when individual traits do not segregate outside the parental range but their combination results in functional transgression (Dittrich‐Reed and Fitzpatrick 2013; Holzman and Hulsey 2017; Thompson et al. 2021). Because these novel phenotypes may perform new functions or facilitate expansion into new areas of functional space, they also represent important variation with potential impacts on hybrid fitness and on the evolutionary trajectory of the species. In addition to increasing heritable phenotypic variation, hybridization can also relax the genetic constraints and covariance structures between certain traits, generating novel trait combinations that may facilitate expansion into new areas of functional space (Selz et al. 2014). Quantifying general patterns of phenotype expression in hybrids is therefore important to help clarify if, and by which mechanism, phenotypic diversification may emerge from hybridization.

One way in which hybridization can influence the trajectories of species is through effects on behavioral and cognitive phenotypes. Although hybrids may show maladaptive behaviors, such as impaired foraging, compromised reproductive behaviors, or poor learning and memory (Linn et al. 2004; Bridle et al. 2006; Turissini et al. 2017; McQuillan et al. 2018; Pärssinen et al. 2020), hybridization can also drive behavioral novelty and promote evolvability. For example, African cichlid hybrids show higher feeding efficiency on novel food types, even though they have lower efficiency on parental food types, indicating they may have an advantage in ecological contexts outside the parental species’ niches (Selz and Seehausen 2019). In addition, behavioral novelty may also arise indirectly from transgression in related traits, such as morphological traits. Hybrids of two Galapagos finches showed transgressive segregation of bill size and shape, which allowed them to forage on novel food items and become ecologically successful, ultimately leading to the formation of a new species (Lamichhaney et al. 2018). Similarly, transgression in jaw morphology of hybrids of two African cichlids was observed together with a novel sand‐sifting foraging behavior, a specialized mode of feeding on a resource that neither parental species typically exploits (Feller et al. 2020).

Similar to head morphology and associated foraging behaviors, one morphological trait that is tightly linked to behavioral phenotypes and that can have important fitness consequences is brain anatomy. Variation in overall brain size and in the relative size and function of different brain regions is ubiquitous in nature, and this variation often correlates with behavioral and cognitive traits. For example, larger overall brains or larger sizes of certain brain regions tend to positively correlate with spatial learning and memory, self‐control, cognitive flexibility, and innovation and problem‐solving (Lefebvre et al. 2004; Deaner et al. 2007; Herculano‐Houzel 2017; Buechel et al. 2018; Triki et al. 2022). In addition, both brain morphology and cognitive traits are associated with survival, reproduction, or colonisation success, among others (Dukas and Bernays 2000; Sol et al. 2005; Cole et al. 2012; Kotrschal et al. 2013; Madden et al. 2018). Therefore, any effects of hybridization on brain morphology and/or cognitive traits are likely to be important for hybrid ecological success and fitness. Although the role of hybridization as a driver of variation and novelty in several morphological traits linked to fitness is well recognized, it remains largely unknown whether hybridization can fuel behavioral novelty by promoting phenotypic variation in brain morphology and/or cognitive traits.

To address this empirical gap, here we investigate the effect of hybridization on brain anatomy, learning ability, and cognitive flexibility in F1 and F2 hybrids of two closely related fish species (*Poecilia reticulata* and *Poecilia wingei*). Our previous study of learning ability and cognitive flexibility in F1 females of this cross showed that female hybrids had slightly higher dispersion in cognitive traits, some hybrid individuals had transgressive trait scores, and the mean phenotype of one hybrid group deviated away from the axis of variation of the parentals, suggesting that hybridization may promote cognitive variation and generate new trait combinations (Vila‐Pouca et al. 2022). These results on F1 female hybrids provided an important first test of whether hybridization can promote cognitive variation, a prerequisite for hybrids to have cognitive innovation potential (Seehausen 2013; Selz and Seehausen 2019). However, a few questions were left unanswered. Because only F1 individuals were tested, we could not assess if the observed increased phenotypic variation was due to heterosis, and is therefore transient, or if hybrid phenotypic variation in cognitive abilities is observed in subsequent hybrid generations and may therefore be heritable. Furthermore, the observed asymmetry between the reciprocal crossings of F1 hybrids (only one hybrid group was mismatched with the parentals) suggested the contribution of uniparentally inherited genetic factors to the traits analyzed. Yet, only females were tested, which are the homogametic sex in *Poecilia* guppies; examining if greater reciprocal asymmetries are observed in males compared to females is a vital test to assess whether asymmetries derive from uniparentally inherited factors. Finally, a few studies have suggested that behavioral novelty may emerge from transgression in morphological traits linked to fitness (Lamichhaney et al. 2018; Feller et al. 2020). Because brain morphology is tightly linked to behavior and cognition, and both brain and cognitive traits have important fitness consequences, is it essential to include brain morphology analyses if we are to gain a complete understanding on whether hybridization may fuel behavioral novelty through effects on the brain and/or cognitive abilities.

The present study therefore aims to address these questions by testing both males and females of F1 and F2 fish and by examining not only learning and cognitive flexibility but also overall brain size and brain region anatomy. Using probability density estimation methods combined with an analytical geometry approach, we quantify patterns of phenotype expression in hybrids and test if hybrid phenotypes are overdispersed relative to parentals and/or deviate from parental mean phenotypes. If hybrids show poorer or intermediate brain and/or cognitive traits, then these traits may represent a postzygotic isolating barrier against hybridization (Rice and McQuillan 2018; Rice 2020). This is because hybrids with intermediate traits can be ecologically mismatched with either parental environment if there is divergent ecological selection acting on parental species, for example, arising from differences in their environment, such as habitat structure, predation, or competition, or from sexual selection of traits linked to mate recognition, among other causes of ecological speciation (Rundle and Nosil 2005). Additionally, hybrids with intermediate traits have invested energy into trait development but without obtaining the benefits they should provide, at least compared to the parental group with the most developed traits (Niven and Laughlin 2008; Dunlap and Stephens 2016). In such circumstances, hybrids with intermediate traits will be at a disadvantage with parentals and will be selected against, leading to postzygotic isolation (Rundle and Nosil 2005). However, if hybrid phenotypes are overdispersed relative to parents or deviate from parental mean phenotypes, then those hybrids may have cognitive innovation potential (Seehausen 2013; Thompson et al. 2021). Additionally, we may expect differences between F1 and F2 hybrids depending on the traits’ genetic architecture, namely, if heterosis is the mechanism underlying phenotypic variation in F1 fish. Furthermore, we may expect brain anatomy traits to be particularly affected by hybridization if covariation between traits is relaxed (Selz et al. 2014; Johnson et al. 2015) and leads to a releasing of the energy trade‐offs that constrains variation in brain region sizes (Niven and Laughlin 2008; Kotrschal et al. 2013).

## Methods

### PARENTAL SPECIES AND HYBRID BREEDING

*Poecilia wingei* is evolved from *P. reticulata* (Meredith et al. 2010) and occurs in a small area in northern Venezuela, largely separate from the ubiquitous *P. reticulata*. Although the two species seem to have a very similar ecology (M. Kempkes pers. comm.), males differ in color pattern and courtship behavior (Alexander and Breden 2004; Poeser et al. 2005), and because those differences are most pronounced in areas where they co‐occur (Poeser et al. 2005) it is assumed that the species are separated by divergent sexual selection (Alexander and Breden 2004). Although field data on hybridization are lacking, hybridization events seem likely as *P. wingei* and *P. reticulata* are genetically compatible (Alexander and Breden 2004) and readily hybridize in the laboratory (Ramsay 2014).

Parental fish used for breeding were derived from laboratory populations that were kept in identical conditions at Wageningen University & Research. Common guppy (*P. reticulata*) populations descended from wild individuals from the upper Aripo river, Trinidad, and Endler's guppy (*P. wingei*) populations descended from wild individuals from Cumaná, Venezuela in 2006. Experimental fish were produced according to standard hybridization methods (Stelkens et al. 2009; Vila‐Pouca et al. 2022). In brief, nonhybrid (parental) crosses and reciprocal first‐generation (F1) hybrids (i.e., *P. reticulata* females crossed with *P. wingei* males, and *P. wingei* females crossed with *P. reticulata* males) were bred simultaneously, in January and August 2020. Second‐generation (F2) hybrids were then obtained by crossing females and males from F1 hybrid stock populations. To ensure that focal F2 hybrids were compared to parental individuals of the same age, F2 fish were bred simultaneously with a group of common and Endler's guppies, in January 2021. All groups of parental, F1, and F2 fish were bred and raised in identical conditions in a common garden environment and examined for brain morphology or cognitive traits at approximately 1 year of age. In the F1 cohort, F1 hybrids and parental offspring were obtained each from 12–16 females (Vila‐Pouca et al. 2022). In the F2 cohort, F2 hybrids were obtained each from 16 females and parental offspring each from 10–12 females. All aquaria were part of a recirculation system, in which water chemistry, temperature, light conditions, and feeding regime were kept constant. For further details on breeding and rearing, see Supporting Information S1.1 and Vila‐Pouca et al. (2022). We will refer to the parental species as R for *P. reticulata* and W for *P. wingei* and use these abbreviations for the hybrid crosses as mother species × father species (i.e., R×W and W×R).

### ETHICS

The care and use of experimental animals complied with animal welfare laws, guidelines, and policies as approved by the Animal Welfare Body (Instantie voor Dierenwelwijn, IvD) at Wageningen University and Research and the Centrale Commissie Dierproeven (AVD10400202010625), Netherlands.

### BRAIN MORPHOLOGY MEASUREMENTS

Both males and females from each crossing group (*n* = 232; detailed sample size in Supporting Information S2) were euthanized in a water bath containing an overdose of 2‐phenoxyethanol. Lateral view photographs were taken with a Nikon DSLR camera immediately after euthanasia. Fish were then fixed with 4% paraformaldehyde in phosphate‐buffered saline (PBS) for 2 weeks. The samples were then washed twice in PBS and stored at 4°C pending dissections. Standard length of each fish (tip of the snout to the end of the caudal peduncle) was measured from photographs using *Fiji* (Schindelin et al. 2012). Whole brains were dissected out from each fish and photographed from a dorsal, right lateral, left lateral, and ventral view using a ZEISS SteREO Stemi 305cam microscope with integrated camera. Brains were stored in PBS and later weighed to the nearest 0.01 mg using a Mettler Toledo XPE105 analytical scale. The length, width, and height of six major brain regions (telencephalon, optic tectum, cerebellum, dorsal medulla, hypothalamus, and olfactory bulbs) were measured using *Fiji* (Schindelin et al. 2012). The volume of each brain region was then estimated using: *V* = (*L* × *W* × *H*)π/6 (White and Brown 2015). All dissections were performed by the same researcher and body size and brain measurements were taken blind to species group.

### LEARNING PERFORMANCE

**Figure 1:**
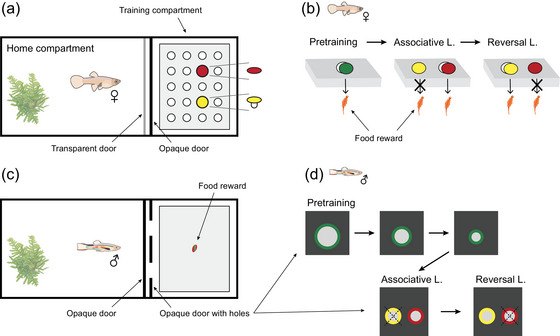
Schematic representation of the experimental tank and associative and reversal learning tasks for females (a, b) and males (c, d). For both experiments, the experimental tank consisted of a home compartment and a training compartment, accessible through guillotine doors operated by the researcher conducting the experiment (a, c). Females were pretrained to dislodge a green plastic disc to access a food reward and tested for associative and reversal learning using red and yellow discs (b). Males were pretrained to access the experimental chamber and obtain a free food reward by swimming through a hole with a green edge and tested for associative and reversal learning using holes with red and yellow edges (d).

Fish from each sex and crossing group (*n* = 278; detailed sample size in Supporting Information S2) were tested for associative learning and cognitive flexibility at approximately 1 year of age. Females were tested in a color discrimination assay that tests associative learning and in a reversal learning assay that tests cognitive flexibility, using red and yellow as stimulus colors (Fig. 1a,b; Buechel et al. 2018; Fuss and Witte 2019; Vila‐Pouca et al. 2022). F1 and parental females were tested with this assay in a previous study; detailed methods and results are given in Vila‐Pouca et al. (2022), and those data are included here as part of this larger study. For the present study, F2 and parental females were tested in September–October 2021. Because males have proven difficult to train with this protocol (unpublished pilot study; Fuss and Witte 2019), parental, F1, and F2 males were tested in this study in a different color discrimination task (Fig. 1c,d) adapted from Laland and Williams (1997), in November–December 2021. For both female and male assays, experimental fish were individually housed in the experimental tank. Each tank included a home compartment (45 × 20 cm) and an experimental compartment (15 × 20 cm) at the front of the tank. Fish were confined to the home compartment outside of training sessions. During training sessions, fish had access to the experimental compartment through a guillotine door operated by the researcher conducting the trials (Fig. 1a,c). The experimental compartment was visually isolated from neighbors to avoid social learning effects. To control for potential color biases, fish were randomly assigned to either red or yellow as the correct stimulus, balanced across the crossing groups. Trials were run blind to species group.

#### Pretraining: Females

The goal of the pretraining was to train females (*n* = 194) to dislodge a green disc to access a food reward (one defrosted *Artemia*) hidden in a hole underneath. During the first trials, the disc only partially covered the hole, leaving the reward exposed. We then gradually trained the fish to dislodge the disc by moving it from partially to fully covering the hole (over an average of 30 trials; additional details are in Vila‐Pouca et al. [2022] and Supporting Information S1.2). A total of 185 females (R, *n* = 40; W, *n* = 40; F1 R×W, *n* = 27; F1 W×R, *n* = 29; F2 R×W, *n* = 25; F2 W×R, *n* = 24) successfully retrieved the food reward by dislodging the disc in all trials of the last two sessions and continued the experiment.

#### Associative learning: Females

After being trained to retrieve a reward underneath a movable disc, females (*n* = 185) were tested in their ability to learn a color association. In this task, females were given a choice between a red and a yellow disc, both concealing a food reward. The disc assigned as the correct color for that fish could be dislodged to reveal the reward; the incorrect color disc was stuck in the hole with a plastic knob and could not be moved, preventing the fish from retrieving the food underneath. Because both the correct and incorrect options contained a hidden reward, fish could not rely on olfactory cues to learn the task. To control for potential side biases, we randomized the position (left/right) of the correct color in each trial. Choice was recorded as the first disc touched by the fish. The fish was given 1 min to dislodge the correct colored disc and eat the reward. If fish made an incorrect choice, correction was allowed within 3 min. If the fish failed to make any choice within 1 min or to correct its choice within 3 min, we moved the rewarded disc 5 mm to the side to allow easy access to the food and ensure that all fish obtained a reward in each trial. Fish received daily sessions of three or four trials, excluding weekends. All fish ran a minimum of 12 trials. The learning criterion consisted of seven consecutive correct choices (significant according to a binomial probability). As soon as a female ran 12 trials and reached the learning criterion, the next phase commenced. If a female did not reach the learning criterion within 40 trials, it was excluded from further training (R, *n* = 9; F1 R×W, *n* = 1; F1 W×R, *n* = 1; F2 R×W, *n* = 3; F2 W×R, *n* = 5).

#### Reversal learning: Females

After succeeding in the color association task, the reversal learning task started. The procedure was the same except the reward contingency was reversed: fish previously trained on yellow were now trained on red and vice versa. Each female ran a minimum of 24 training trials and continued the reversal task until it reached seven consecutive correct choices, up to a maximum of 60 trials.

#### Pretraining: Males

The goal of pretraining was to train males (*n* = 84) to swim through a hole in a partition to access the experimental chamber and obtain a food reward. It is worth noting that due to laboratory space constraints, the sample size of males initially included in the assays (*n* = 14 per group) is smaller than that of females. To familiarize the fish with obtaining food in the experimental chamber, we started by giving males three trials per day over 2 days where a guillotine door was lifted by the experimenter to give access to the experimental chamber and to a free food reward (a small portion of flake placed on the white tank floor). From the third day, when the guillotine door was lifted, the fish encountered a partition with a circular hole that gave them access to the experimental chamber. The edge of the hole was painted green to make the fish familiar with swimming through a colored hole. We gradually reduced the size of the circular hole (from 5.5 mm ø to 4.5 mm ø to 3 mm ø) over training trials (Fig. 1d). We performed four daily trials over 8 days (with rest days on Thursday and Sunday; additional details in Supporting Information S1.3). On the last day of pretraining, a total of 74 males (R, *n* = 13; W, *n* = 14; F1 R×W, *n* = 11; F1 W×R, *n* = 11; F2 R×W, *n* = 13; F2 W×R, *n* = 12) successfully swam through the 3‐mm ø green hole and ate the reward within 3 min in all trials of the last session, and therefore continued the experiment.

#### Associative learning: Males

After being trained to swim through a hole in a partition to access a food reward, males (*n* = 74) were tested in their ability to learn a color association. In this task, when the guillotine door was lifted, males were given a choice between a hole with a red edge and a hole with a yellow edge to access the experimental chamber and obtain the food reward (Fig. 1d). Fish with red assigned as the correct color were allowed to swim through the red hole, but not the yellow hole, which was blocked with a transparent acrylic sheet; the opposite was true for fish trained to yellow. To control for potential side biases, we randomized the position (left/right) of the correct colored hole in each trial. Choice was recorded as the first hole the fish attempted to swim through. The fish was given 3 min to swim through the correct colored hole and eat the reward. For incorrect trials, where fish kept pushing against the transparent sheet, correction was allowed within 5 min. If the fish failed to make any choice within 3 min or to correct its choice within 5 min, we covered the incorrect option with an opaque sheet and only the correct hole was visible; this ensured that all fish experienced a reward in each trial. Fish received daily sessions of four trials, excluding Thursdays and Sundays. All fish ran a minimum of 16 trials. The learning criterion consisted of seven consecutive correct choices (significant according to a binomial probability). As a control for the presence of the transparent sheet blocking the incorrect option, we ran two daily test sessions as soon as each fish ran at least 16 trials and reached the learning criterion. Test sessions consisted of five unrewarded trials where both red and yellow holes were unblocked interspersed with five regular trials. Fish progressed into the reversal learning task after the test. If a male did not reach the learning criterion within 40 trials, it was excluded from further training (R, *n* = 7; F1 R×W, *n* = 6; F2 R×W, *n* = 4; F1 W×R, *n* = 4; F2 R×W, *n* = 3; F2 W×R, *n* = 4).

#### Reversal learning: Males

After succeeding in the color association task, the reversal learning task started. The procedure was the same except the reward contingency was reversed: fish previously trained to swim through the red hole were now trained to the yellow hole and vice versa. Each male ran the reversal task until it reached seven consecutive correct choices, up to a maximum of 60 trials. One male (F1 W×R) was suddenly found dead in the housing compartment on day 5 of the reversal task; because it only ran 16 trials, it was excluded from the learning criterion analyses.

At the end of the experiment, 80 males (12–14 individuals of each of the six groups) were euthanized to complement the brain morphology dataset. To keep exposure to the learning task similar across all individuals, fish continued running one trial per day after they succeeded the task until all fish completed the learning experiment. It is unlikely that brain morphology comparisons between species groups are influenced by learning effects because our sample is balanced over groups, and a previous study in guppies found that learning does not impact coarse brain plasticity (Fong et al. 2019).

### STATISTICAL ANALYSIS

Statistical analyses were performed in R version 3.6.3 (R Core Team 2020). Linear models were generated using “*lme4*” (Bates et al. 2015). Comparisons with null models without interaction terms were done using the “*anova*” function from “*stats*” (R Core Team 2020). Model terms were tested for significance using the “*Anova*” function in “*car*” (Fox and Weisberg 2019). If species group was a significant predictor in the model, we assessed Tukey corrected multiple comparisons between species levels using the “*glht*” function in “*multcomp*” (Hothorn et al. 2008). Code and model results are available in the Supporting Information.

#### Brain morphology

To test for species differences in relative brain size, brain weight was fit to a linear model with the predictors of species group, body size, sex, and all two‐way interactions. Body size was fitted as a covariate to account for allometry and focus on species differences in relative brain size. To test for species differences in relative brain region volumes, each brain region volume was fit to a linear model with the predictors of species group, brain remainder volume, sex, and all two‐way interactions. Brain remainder volume (total brain volume minus each region volume) was used instead of total brain volume to avoid statistical confounds resulting from the inclusion of the region being analyzed in the covariate. To achieve normality and linearize the allometries between response variables and predictors, we log‐transformed body size (mm), brain weight (mg), and brain region volume (mm^3^). For all models, two‐way interactions were removed if they were not significant and not relevant for our study question.

#### Learning performance

To test for species differences in learning performance, we compared for each learning task (i) number of trials to reach learning criterion using a generalized linear model (Poisson distribution) with the predictors of species group and color and (ii) learning rate, that is, probability of success per trial (correct = 1; incorrect = 0) using a generalized linear mixed‐effect model (binomial distribution) with the predictors of trial number, species group, correct disc color, and the interaction of trial number × species group and trial number × color, as well as a random intercept and slope for fish identity to account for repeated observations of individual fish. We tested the significance of the random effects in models (ii) with likelihood ratio tests, by comparing models that culled the intercept or slope term to our final model. To examine if performance in the associative learning task had carryover effects on performance in the reversal task, we tested the inclusion of the predictor “trials to learn the associative learning task” and its interaction with species group in models (i, ii) for the reversal task. For model (ii) in the reversal task, trial number was log‐transformed to meet the assumption of linearity on the logit scale. These analyses were run separately for females and males. Due to the low sample size of males that succeeded the reversal learning task, we could not run model (i) to compare the number of trials to reach learning criterion. Additionally, for the test trials of male fish, a nonparametric one‐sample Wilcoxon rank sum test was used to compare the observed proportion of correct choices per individual against a chance value of 0.5 (null hypothesis of learning the task using cues from the transparent sheet blocking incorrect option).

#### Phenotypic trait variation and transgression

To quantify phenotypic dispersion, identify transgressive hybrids, and compare the mean phenotypes of species groups, we used a recent methodology developed to quantify functional transgression in multidimensional phenotypes (Mérot et al. 2020; Thompson et al. 2021; Vila‐Pouca et al. 2022). For brain morphology analyses, we ran an exploratory analysis to combine relative whole brain and brain region traits to define the brain morphospace of each species; however, PCA results were suboptimal (Supporting Information S5.1 and S5.2) and therefore we focused on brain weight relative to body size and on telencephalon and optic tectum volume because these are the brain regions most likely to be important neural correlates of cognitive abilities such as perception and learning (Broglio et al. 2011; Triki et al. 2022). Additionally, because we found in our brain morphology analyses little to no differences by sex on relative brain size and brain region volume (see *Results* below), we chose to pool females and males for the transgression analyses in brain traits to increase sample size and strengthen statistical power. For cognitive performance analyses, we used individual scores in the associative and reversal learning tasks (number of trials to learn), following Vila‐Pouca et al. (2022). The number of trials to learn was log‐transformed to approximate a continuous variable with Gaussian distribution.

Kernel density estimation (KDE) clusters of trait space (in three‐dimensional for brain morphology and two‐dimensional for cognitive performance) were computed with the “*kde*” function in “*ks*” (Duong 2021), with the bandwidth estimated using a grid‐search estimation. Phenotypic dispersion was estimated as the hypervolume of the KDE cluster that contains 95% of the individuals in the group (Mérot et al. 2020; Vila‐Pouca et al. 2022) with the “*contourSizes*” function in “*ks*.” We considered hybrid phenotypes to be transgressive if their trait values fell outside the range of both parental species, that is, outside the 95% KDE of both parents (Stelkens et al. 2009; Dittrich‐Reed and Fitzpatrick 2013). For each hybrid group, we quantified the frequency and proportion of transgressive individuals using a custom‐made function (available in the Supporting Information) based on the packages “*ks*,” “*misc3d*,” and “*akima*” (Feng and Tierney 2008; Duong 2021; Akima and Gebhardt 2022). To test if the mean phenotypes of our hybrid groups deviate from a linear combination of the parental phenotypes (Thompson et al. 2021), we simulated hybrid phenotypes by randomly sampling a pair of individuals from each parental species and calculating the mean trait values of the parental pair. To approximate the sample size of our experimental groups, we generated 35 simulated hybrids for brain morphology and 25 simulated hybrids for cognitive performance and repeated this process 100 times. We then compared our experimental hybrids’ mean phenotype with that of simulated hybrids in “parental bias,” which captures deviation from the arithmetic mean of the parental phenotypes in the direction of either parent, and “phenotypic mismatch,” which captures deviation away from the line connecting parental mean phenotypes (Mérot et al. 2020; Thompson et al. 2021; Vila‐Pouca et al. 2022). The two metrics were estimated by analytical geometry calculations using a custom‐made function (available in the Supporting Information).

## Results

### PARENTAL SPECIES AND HYBRIDS DIFFER IN SOME BRAIN MORPHOLOGY TRAITS

**Figure 2:**
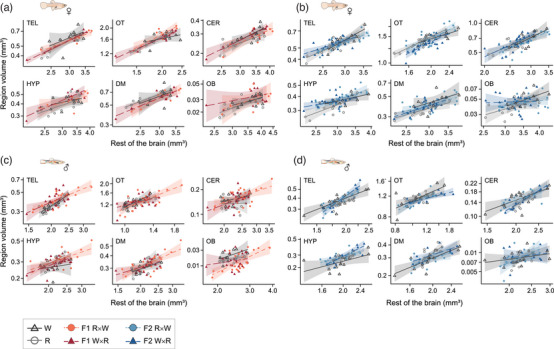
Brain region volume relative to brain remainder volume (total brain volume minus each region volume) of parental and F1 females (a), parental and F2 females (b), parental and F1 males (c), and parental and F2 males (d). Remainder brain volume (*x*‐axis) and brain region volume (*y*‐axis) are shown in log10 scales. Model predictions are plotted as the best fit line with 95% confidence interval. Parental groups are given in shades of gray; F1 hybrids are given in shades of red; F2 hybrids are given in shades of blue.

When comparing parentals and F1 hybrids, we found a strong allometry between brain weight and body length (*n* = 107, estimate = 1.17, SE = 0.11, *F*_1,101_ = 117.34, *P* < 0.0001) and no differences in allometry between species groups or sex (both interactions *P* > 0.05; Supporting Information S3.1.1). After accounting for these effects, we found differences among parentals and F1 hybrids in relative brain size (*F*_3,101_ = 5.07, *P* = 0.003) with Endler's guppies (W) showing smaller relative brain size compared to common guppies, R (est. = −0.050, SE = 0.014, *P* = 0.003) and to F1 R×W (est. = −0.034, SE = 0.012, *P* = 0.025). The analysis of brain region volumes, after accounting for the allometry associated with brain remainder volume (all *P* < 0.0001; Supporting Information S3.1.2), revealed no differences in allometry between parentals and F1 hybrids or by sex (interactions all *P* > 0.05; Fig. 2a,c) and no differences in relative brain region volume between species groups except for olfactory bulb volume (*F*_3,101_ = 2.80, *P* = 0.044), with Endler's guppies (W) showing larger relative olfactory bulbs compared to F1 R×W (F1 R×W – W: est. = −0.15, SE = 0.051, *P* = 0.026). This indicates that species differences in total brain size were overall equally expressed over the different brain regions. We also found an effect of sex on some brain region volumes, with males having a larger relative optic tectum but smaller cerebellum, dorsal medulla, and hypothalamus than females (Supporting Information S3.1.2; Fig. 2a,c).

When comparing parentals and F2 hybrids, we found a strong allometry between brain weight and body length that varied by sex (*n* = 125, est. = 0.88, SE = 0.24, *F*_1,118_ = 12.89, *P* < 0.001) but not between species groups (*F*_3,112_ = 0.77, *P* = 0.51; Supporting Information S3.2.1). After accounting for these effects, we found no differences among parentals and F2 hybrids in relative brain size (*F*_3,118_ = 1.45, *P* = 0.23). The analysis of brain region volumes, after accounting for the allometry associated with brain remainder volume (all *P* < 0.01; Supporting Information S3.2.2), revealed no differences in allometry between parentals and F2 hybrids (interactions all *P* > 0.05; Fig. 2b,d) or differences in allometry by sex except for telencephalon volume (sex × brain remainder volume, est. = 0.40, SE = 0.15, *F*_1,118_ = 7.23, *P* = 0.008; Supporting Information S3.2.2). We found differences between species groups in telencephalon, optic tectum, and hypothalamus volume, with F2 W×R showing a larger relative telencephalon compared to parental common guppies, R (F2 W×R – R: est. = 0.033, SE = 0.011, *P* = 0.017) and smaller relative optic tectum compared to parentals R and W and to F2 R×W (F2 W×R – R: est. = 0.030, SE = 0.010, *P* = 0.006; F2 W×R – W: est. = 0.026, SE = 0.010, *P* = 0.035; F2 W×R – F2 R×W: est. = 0.021, SE = 0.010, *P* = 0.043), and with both F2 hybrids showing a larger relative hypothalamus compared to parental Endler's guppies, W (F2 R×W – W: est. = 0.049, SE = 0.015, *P* = 0.008; F2 W×R – W: est. = 0.047, SE = 0.015, *P* = 0.014; Supporting Information S3.2.2; Fig. 2b,d).

### PARENTAL SPECIES AND HYBRIDS DO NOT DIFFER IN LEARNING PERFORMANCE

Over 90% of females could be successfully pretrained to dislodge the disc (R, 40/43; W, 40/41; F1 R×W, 27/30; F1 W×R, 29/30; F2 R×W, 25/25; F2 W×R, 24/25), with 185 females progressing to the learning tasks.

**Figure 3:**
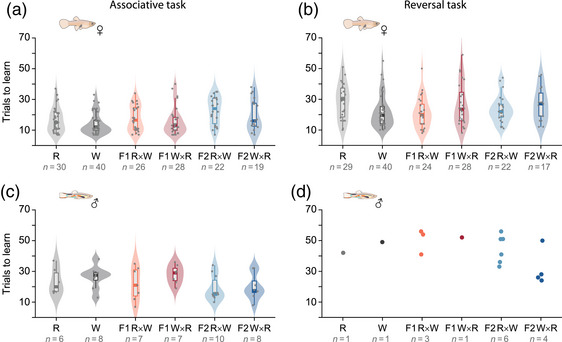
Number of trials needed to reach learning criterion in the associative learning (a, c) and reversal learning (b, d) tasks, for females (top) and males (bottom) of each species group. Note that data for parental groups in female plots (a, b) are pooled from the experiments in Vila‐Pouca et al. (2022) and the present study for ease of visualization.

Detailed results on F1 hybrid females’ performance in both associative and reversal learning are published elsewhere (Vila‐Pouca et al. 2022). In brief, we found that F1 hybrids showed overall intermediate learning ability between the two parental groups in the two tasks, as seen by the overlap in range and median values of trials to learning criterion in Figure 3a,b (Vila‐Pouca et al. 2022).

When comparing F2 hybrid and parental females in the associative learning task, we found that F2 W×R hybrids had the lowest success rate with only 79% (19/24) females succeeding in the task compared to 88% (22/25) in F2 R×W and to 85% (11/13) and 100% (10/10) in parental common (R) and Endler's (W) guppies, respectively, yet differences between groups were not significant (Supporting Information S4.2.1). When comparing the number of trials needed to learn the color association, we find that F2 R×W performed worse than parentals, taking significantly longer to succeed compared to common guppies (Fig. 3a; F2 R×W – R: est. = 0.25, SE = 0.084, *P* = 0.014; Supporting Information S4.2.1). Comparing learning rates, all four groups improved their success at a similar rate (species × trial number: *χ* ^2^ = 2.98, df = 3, *P* = 0.40; Supporting Information S4.2.1).

In the reversal learning task, success rates of F2 hybrid and parental females were very high with 100% (22/22) of F2 R×W, 90% (17/19) of F2 W×R, and 100% of both R (11/11) and W (10/10) females reaching learning criterion (no significant differences between groups; Supporting Information S4.2.2). Contrary to the associative learning task, we found that common guppies performed worse than F2 R×W hybrids, needing more trials to succeed in the reversal learning task (Fig. 3b; F2 R×W – R: est. = 0.19, SE = 0.072, *P* = 0.041; Supporting Information S4.2.2). All four groups improved their success at a similar rate as the task progressed (species × log trial number: *χ* ^2^ = 0.13, df = 3, *P* = 0.99; Supporting Information S4.2.2). Importantly, similar to our previous results with F1 females (Vila‐Pouca et al. 2022), we found a weak effect of the associative learning task on reversal learning performance with only W females showing a negative correlation between trials needed to learn the association and trials needed to learn the reversal task (species W × trials to success in A.L.: est. = −0.029, SE = 0.010, *z* = −2.80, *P* = 0.005; Supporting Information S4.2.2).

In males, success in pretraining the fish to swim through a hole to access a food reward was above 90% for parentals and F1 hybrids but of 79% for both F2 hybrid groups (R, 13/14; W, 14/14; F1 R×W, 11/14; F1 W×R, 11/14; F2 R×W, 13/14; F2 W×R, 12/13). At the end of pretraining, 74 males progressed to the learning tasks.

In the associative learning task, only 46 out of 74 males succeeded in learning to access the reward through the correctly colored hole. Comparing species groups, the success rate of parental males was slightly lower than F1 and F2 hybrid groups, and F2 R×W showed the highest success rate, yet differences between groups were not significant (R, 46%; W, 57% F1 R×W, 64%; F1 W×R, 64%; F2 R×W, 77%; F2 W×R, 67%; Supporting Information S4.3.1). Of the males that succeeded, we found that males from the F2 groups were slightly faster in learning the color association (Fig. 3c), but the differences were only significant between F2 R×W and F1 W×R (est. = –0.33, SE = 0.10, *P* = 0.021; Supporting Information S4.3.1), likely due to our small sample size. Comparing learning rates, all groups improved their success rate as the task progressed indicating learning of the task contingencies (*χ* ^2^ = 44.57, df = 1, *P* < 0.001; Supporting Information S4.3.1); no differences were detected in learning rate between parental and hybrid groups (species × trial number: *χ* ^2^ = 4.84, df = 5, *P* = 0.44; Supporting Information S4.3.1). As a control for the presence of the transparent sheet blocking the incorrect option during the associative learning trials, we ran unrewarded test trials where both correct and incorrect options were unblocked. Males showed a significant preference for the correct colored option during the test (mean % correct = 79%, 95% CI = 75%–83%; Wilcoxon rank sum test: *V* = 986.5, *P* < 0.001; Supporting Information S4.3.2), showing that they learnt the task using the color cues surrounding the hole.

In the reversal learning task, success rates of male fish were very low, with only 16 of 46 fish reaching learning criterion. F2 R×W were the most successful group with six out of 10 fish (60%) learning the task, followed by four out of seven fish (57%) of F2 W×R and three out of seven fish (43%) of F1 R×W. Parental groups and F1 W×R had the lowest success rates with a single individual reaching learning criterion (R, 17%; W, 13%; F1 W×R, 17%), yet differences between groups were not significant (Supporting Information S4.3.3). Due to the low success rate, we could not statistically compare the number of trials taken to learn per species group (Fig. 3d; Supporting Information S4.3.3). Regarding the probability of choosing the correct option over trials for the 46 males that participated in the task, all species groups improved their success rate as the task progressed (*χ* ^2^ = 67.71, df = 1, *P* < 0.001; Supporting Information S4.3.3) and the rate of learning was similar between parental and hybrid groups (species × log trial number: *χ* ^2^ = 3.01, df = 5, *P* = 0.70; Supporting Information S4.3.3).

### PHENOTYPIC DISPERSION IN BRAIN MORPHOLOGY IN F1 BUT NOT F2 HYBRIDS

**Figure 4:**
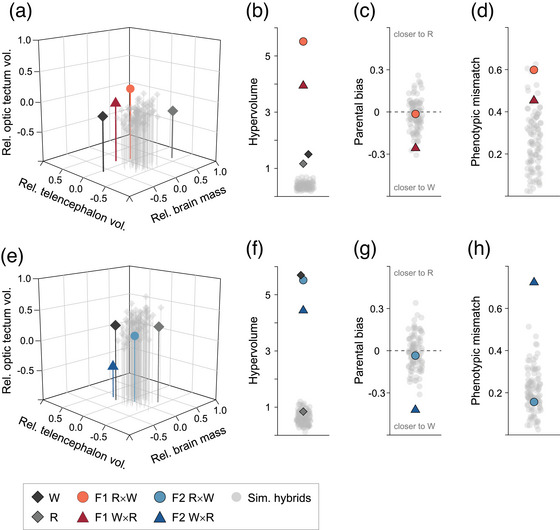
Summary of phenotypic variation and transgression in brain morphology for parentals and F1 hybrids (a–d) and for parentals and F2 hybrids (e–h). Position of the mean phenotype of hybrid and parental groups and of simulated hybrid populations (a, e). Hypervolume of observed and simulated 95% kernel density estimations (KDEs) as an estimate of phenotypic dispersion (b, f). Distance of observed and simulated mean hybrid phenotypes from the midpoint between the parentals as an estimate of parental bias (c, g). Deviation of observed and simulated mean hybrid phenotypes from the line connecting parental mean phenotypes as an estimate of phenotypic mismatch (d, h). Note that data for females and males are pooled together.

Comparing F1 hybrids and parentals (females and males pooled together), the hypervolume of brain morphospace describing phenotypic dispersion of F1 hybrid groups was over three times larger compared to parental groups and simulated hybrids, and larger for F1 R×W compared to F1 W×R (Fig. 4a,b; Supporting Information S5.1). The frequency of F1 hybrid individuals found outside parental ranges, considered as transgressive, was of 9% for F1 R×W (*n* = 3) and 6% for F1 W×R (*n* = 2) individuals, thus at levels close to the 5% expected by chance (Supporting Information S5.1). Regarding a deviation of the mean hybrid phenotypes, we found that both parental bias and phenotypic mismatch were low because the trait values of F1 R×W and F1 W×R largely overlapped with simulated hybrid populations, although there was a tendency for some phenotypic mismatch of F1 R×W (Fig. 4a,c,d; Supporting Information S5.1).

When comparing F2 hybrids and parentals (females and males pooled together), the phenotypic dispersion of brain morphospace of F2 hybrid groups was similar to one parental group (Endler's guppies, W). Common guppies (R) and simulated hybrids showed lower dispersion (Fig. 4f; Supporting Information S5.2). The frequency of transgressive F2 hybrids was of 5% for F2 R×W (*n* = 2) and 8% for F2 W×R (*n* = 3) individuals, thus also at levels close to the 5% expected by chance (Supporting Information S5.2). Regarding a deviation of the mean hybrid phenotypes, we found that parental bias of F2 R×W was low but F2 W×R hybrids were slightly biased toward Endler's guppies, W (Fig. 4e,g; Supporting Information S5.2). Similarly, the phenotypic mismatch of F2 R×W was low but F2 W×R showed high mismatch with parentals, because the mean phenotype of F2 W×R deviated away from the line connecting parental mean phenotypes toward a smaller relative optic tectum (Fig. 4e,h; Supporting Information S5.2).

### NO HYBRID TRANSGRESSION IN COGNITIVE TRAITS IN F2 HYBRIDS

**Figure 5:**
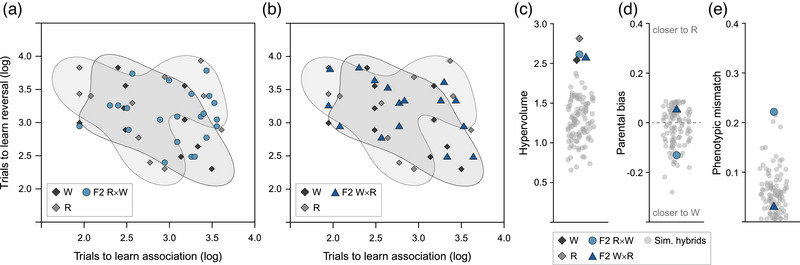
Summary of phenotypic variation and transgression in cognitive traits for female F2 hybrids. Distribution of individual hybrids and parentals in the two‐dimensional “cognitive space,” with shaded 95% parental kernel density estimations (KDEs) (a, b). Hypervolume of observed and simulated 95% KDEs as an estimate of phenotypic dispersion (c). Distance of observed and simulated mean hybrid phenotypes from the midpoint between the parentals as an estimate of parental bias (d). Deviation of observed and simulated mean hybrid phenotypes from the line connecting parental mean phenotypes as an estimate of phenotypic mismatch (e).

Due to the small number of males that succeeded in the reversal learning task, here we could only run phenotypic dispersion and transgression analyses on female cognitive traits. Measures of “cognitive space” describing female F1 hybrid groups are published elsewhere (Vila‐Pouca et al. 2022); in brief, we found that F1 phenotypes had slightly higher dispersion relative to parents, that some hybrid individuals were transgressive, and that F1 R×W had high phenotypic mismatch with parentals (Vila‐Pouca et al. 2022). Comparing female F2 hybrids and parentals, we found no evidence of phenotypic dispersion or transgression in F2 hybrids. The hypervolume of “cognitive space” describing phenotypic dispersion was similar between the four groups (Fig. 5a‐c; Supporting Information S5.4) and none of the F2 hybrid females had trait combinations outside the phenotypic space of the parentals (Fig. 5a,b; Supporting Information S5.4). Regarding a deviation of the mean hybrid phenotypes, we found that both parental bias and phenotypic mismatch were low because the trait values of F2 R×W and F2 W×R largely overlapped with simulated hybrid populations, although there was a tendency toward phenotypic mismatch of F2 R×W (Fig. 5d,e; Supporting Information S5.4).

## Discussion

We investigated brain anatomy, learning ability, and cognitive flexibility in F1 and F2 hybrids of two fish species (*P. reticulata* and *P. wingei*) to characterize patterns of hybrid trait expression and to test if hybridization may promote phenotypic variation in brain morphology and/or cognitive traits. Overall, we found that F1 and F2 hybrids showed intermediate brain anatomy traits, learning ability, and cognitive flexibility compared to the parental groups. Moreover, in both brain and cognitive traits, phenotypic dispersion and transgression were low and hybrid mean phenotypes did not deviate from the axis of variation of the parentals. These results therefore suggest that hybridization is not a strong driver of brain anatomical and cognitive diversification in these Poeciliidae.

Interspecific hybridization is generally assumed to be an important source of heritable phenotypic variation (Seehausen 2004; Mallet 2007; Dittrich‐Reed and Fitzpatrick 2013). Such variation may enlarge the working surface for natural selection and promote the evolutionary potential of hybrid populations (Rieseberg et al. 1999; Seehausen 2013). For example, some hybrids of African cichlids or Galapagos finches show greater variation in morphological and/or behavioral phenotypes and express transgressive traits that allow them to explore ecological opportunities unavailable to the parentals (Lamichhaney et al. 2018; Selz and Seehausen 2019; Feller et al. 2020). The two closely related species studied here, *P. reticulata* and *P. wingei*, seem to have an overall similar ecology, but differ in a few traits, including courtship behavior and male home range size (Alexander and Breden 2004; Poeser et al. 2005). Although *P. reticulata* males vagrantly cover large areas (Croft et al. 2003), *P. wingei* seem to stay within “territories” of 50‐ to 70‐cm diameter (M. Kempkens, pers. comm.). As home range size has been positively correlated with learning performance in spatial tasks (Sherry et al. 1992), learning and/or brain anatomy differences between common and Endler's guppies may be expected. In a previous study, we found that cognitive phenotypes of F1 hybrid females were slightly overdispersed compared to parentals, that some hybrid individuals had transgressive combinations of learning scores, and that F1 R×W female hybrids showed a significant deviation away from the axis of variation of the parentals (Vila‐Pouca et al. 2022). Here, we aimed to expand on these findings by testing both males and females of F1 and F2 hybrid groups and by assessing not only learning and cognitive flexibility but also relative brain size and brain region anatomy. In total, we assayed brain morphology of 232 animals and cognitive abilities of 259 animals. Despite this very large sample size, we found no evidence of phenotypic dispersion or transgression in cognitive phenotypes of F1 males and in F2 females and males, as well as little to no dispersion and transgression in brain anatomy traits of F1 and F2 fish. Therefore, hybridization does not seem to be a prominent promotor of diversification in brain anatomy and cognitive traits, at least in these Poeciliidae species.

What may explain the apparent discrepancy in cognitive results between F1 and F2 fish? We suggest that heterosis, also called hybrid vigor, might explain our previous results on cognitive traits of F1 females because it is only present in first‐generation hybrids (Lippman and Zamir 2007; Proops et al. 2009; Vila‐Pouca et al. 2022). Any fitness advantage from heterosis is generally short lived because recombination in subsequent generations causes hybrid breakdown (Lippman and Zamir 2007), indicating that the phenotypic variation we observed in cognitive traits of F1 females might have no adaptive potential.

Hybridization can alter trait correlations by relaxing phenotypic trade‐offs and genetic correlations (Selz et al. 2014; Johnson et al. 2015). In this study, we expected that brain anatomy traits might be particularly affected by relaxed covariation and a release of energetic trade‐offs constraining brain region development (Niven and Laughlin 2008; Kotrschal et al. 2013), leading to increased phenotypic variation in hybrid groups. However, we found that species differences in brain size were overall equally expressed over the different brain regions and that hybrids showed limited phenotypic dispersion compared to parentals. Furthermore, our main result was that hybrid phenotypes were overall intermediate between parentals, both in relative brain size, brain region volume, learning ability, and cognitive flexibility. Intermediate phenotypes are often at a competitive disadvantage in the presence of parental individuals (Rundle and Nosil 2005; Mallet 2007; Rice and McQuillan 2018; Rice 2020). Regarding the traits under consideration in this study, although the functional and fitness consequences of intermediate brain size and cognitive abilities are unclear, it is possible that hybrids with intermediate traits have invested significant energy into their development but without obtaining the benefits they should provide, at least compared to the parental group with the most developed traits (Niven and Laughlin 2008; Dunlap and Stephens 2016). If hybrids with intermediate phenotypes are indeed disadvantaged compared to parentals, then brain anatomy and/or cognitive traits may represent an extrinsic postzygotic isolating barrier against hybridization (McQuillan et al. 2018).

Two nonmutually exclusive hypotheses may explain the lack of transgression and phenotypic dispersion we found in these traits. One possibility is that *P. reticulata* and *P. wingei*, the two parental species used in this study, are too phylogenetically close, as the amount of transgression has been suggested to increase as a function of the genetic distance between the parental lines (Rieseberg et al. 1999; Stelkens et al. 2009). However, genetic distance may not necessarily predict transgression or any other aspect of trait expression in hybrids (Thompson et al. 2021). Another possibility is that the traits we chose to study are not impacted by hybridization, for example, if they are under consistent directional selection. A previous study on morphological transgression in hybrids between two cichlid fish species found that phenotypic diversity and transgression were reduced in traits with consistent directional selection, in contrast to traits that evolved in response to stabilizing selection (Albertson and Kocher 2005). These results indicate that the effects of hybridization can be limited to specific traits, that is, those that have not diverged in response to strong directional selection (Albertson and Kocher 2005). The basic organization of the brain and associated brain functions are highly conserved in vertebrates and under strong energetic constraints and selection pressures (Jerison 1973; Broglio et al. 2005; Niven and Laughlin 2008; Yopak et al. 2010). It is possible that in our parental species, which share a generally similar ecology (Alexander and Breden 2004; Poeser et al. 2005), brain anatomy and associated cognitive abilities are under consistent directional selection, thereby limiting the necessary genetic variation for transgressive segregation to occur. Additionally, our fish have been in captive conditions in the laboratory for several generations. They were always kept in several large aquaria to keep as much genetic variation in the populations, yet effects from domestication (Marchetti and Nevitt 2003; Burns et al. 2009) cannot be excluded. In the laboratory, natural selection pressures are relaxed, and any other pressures are likely to be in the same direction in both species, potentially eroding or limiting any potential effects of hybridization in these traits.

In our previous study on learning ability and cognitive flexibility in F1 females, we found that F1 R×W hybrids showed a phenotypic mismatch with the parentals, whereas the reciprocal W×R group did not (Vila‐Pouca et al. 2022). This observed asymmetry between reciprocal crossings suggested the contribution of uniparentally inherited genetic factors (Turelli and Moyle 2007). Hybrid asymmetries typically arise from incompatibilities involving uniparentally inherited genetic factors (from mitochondria, chloroplasts, maternal transcripts, or sex chromosomes) and tend to be more pronounced in the heterogametic sex (Turelli and Moyle 2007; Bolnick et al. 2008). In fruit flies, hybrid males (the heterogametic sex) show greater impairment of foraging behavior (Turissini et al. 2017), and in wild chickadees the females (heterogametic sex in birds) show poorer cognitive skills (McQuillan et al. 2018). In the present study, we expanded cognitive testing and brain analyses to males, which are the heterogametic sex in *Poecilia* fish. Although we are unable to draw strong conclusions regarding male cognitive flexibility due to the small number of males that succeeded in the reversal task, our results do not show evidence in favor of stronger asymmetries or incompatibilities in male *Poecilia* fish. Instead, we found that males needed a similar number of trials to learn the color association and the reversal task compared to females, that reciprocal male hybrid crossings showed similar cognitive performance, and that males and females differed little in how hybridization impacted brain anatomy traits.

Taken together, our results suggest that hybridization is not a strong driver of brain anatomical and cognitive diversification in the two Poeciliidae species studied here. As the first test of the impact of hybridization in brain morphology and cognitive traits, our results contribute to further elucidate the role of hybridization as a driver of variation and novelty in important traits linked to fitness. To determine the generality of our conclusions, hybridization experiments with further cognitive tests need to be repeated in other families.

## AUTHOR CONTRIBUTIONS

CVP developed ideas and designed the study with HDW and AK. CVP collected the data with HDW. CVP analyzed the data and wrote the first draft. All authors edited and contributed to finalizing the manuscript.

## CONFLICT OF INTEREST

The authors declare no conflict of interest.

## DATA ARCHIVING

Raw data and analysis code are deposited and freely accessible at https://doi.org/10.6084/m9.figshare.20752093.v1.

Associate Editor: M. Cummings

Handling Editor: T. Chapman

## Supporting information

Supplementary Information
