# Supplementary material for: The effect of experimental hybridization on cognition and brain anatomy: Limited phenotypic variation and transgression in Poeciliidae
Source: Evolution. 2022 Oct 10;76(12):2864–78. doi: 10.1111/evo.14644 (PMC10091962; doi:10.1111/evo.14644)
Supplement: Supplementary file 1 — Supplementary Information [file EVO-76-2864-s001.html]

Supplemental information The effect of experimental hybridization on cognition and brain anatomy: limited phenotypic variation and transgression in Poeciliidae 2022 Evolution


Code 

- Show All Code
- Hide All Code

# **Supplemental information** *The effect of experimental hybridization on cognition and brain anatomy: limited phenotypic variation and transgression in Poeciliidae* **2022 Evolution**

#### C Vila-Pouca, H De Waele, A Kotrschal

Raw data is deposited and freely accessible at https://doi.org/10.6084/m9.figshare.20752093.v1.

# 1 Supplemental Methods

## 1.1 Parental species and hybrid breeding

Common guppy (*Poecilia reticulata*) populations descend from wild individuals caught in the upper Aripo river, Trinidad, and have been kept in captivity for more than 10 generations. Endler’s guppy (*P. wingei*) populations descend from wild individuals from Cumana, Venezuela, and have been kept in captivity for more than 20 generations. For brain morphology analyses, females and males from first-generation (F1) hybrid groups were obtained from three 10L aquaria (30x20x20 cm) stocked with four virgin females and three males, and parental groups were obtained from eighteen 4L (23x15x16 cm) aquaria with one breeding pair. Females and males from second-generation (F2) hybrid groups were obtained from sixteen 4L aquaria stocked with one breeding pair, and parental groups were obtained from ten 4L aquaria stocked with one breeding pair. An additional group of F1 hybrids was bred and raised simultaneously with F2 hybrids, obtained from twelve 4L aquaria stocked with one breeding pair. Before being used for brain analyses, 80 males (12-14 individuals of each of the six groups) were previously tested in the learning tasks. Parental and F1 experimental females used in the learning tasks were bred and tested in two blocks; hybrid offspring were each obtained from three 10L aquaria stocked with four virgin females and three males in the first block, and from sixteen 4L aquaria with one female and one male in the second block. Offspring from parental crosses were each generated from twelve 4L aquaria with one female and one male in both blocks. To note that methods and results of cognitive testing of parental and F1 experimental females are published elsewhere (Vila Pouca et al. 2022). Females from second-generation (F2) hybrid groups used in the learning tasks were obtained from sixteen 4L aquaria stocked with one breeding pair, and parental groups were obtained from ten 4L aquaria stocked with one breeding pair. All aquaria had a bottom layer of gravel, java moss (Taxiphyllum sp.), and bottom-sinking plastic plants. Water temperature was kept at 24 ± 1 ºC and lights were on a 12:12 hr dark/light cycle. Fish were fed twice daily on dried tropical fish flakes or live *Artemia salina* nauplii (brine shrimp). Breeding tanks were checked daily for fry, which were moved to 4L aquaria with gravel and java moss and kept in groups of six until their sex could be identified (females by their gravid spot, and males by the presence of a modified anal fin called gonopodium). Mature fish used in brain morphology analyses were kept in identical single-sex aquaria in groups of 20 individuals, and mature fish used in the cognitive tasks were kept in large single-sex aquaria in groups of 60 individuals, until they were approximately one year old.

## 1.2 Learning assays - females

During pre-training, female fish (n=194) were trained to dislodge a green disc to access a food reward (one frozen Artemia) hidden in one of the holes. Eight females (R, n=2; W, n=1; F1 RxW, n=2; F1 WxR, n=1; F2 RxW, n=1; F2 WxR, n=1) did not feed during early pre-training stages and were replaced by new fish. In total, parental and F1 females ran between 30 and 34 pre-training trials, with the exception of a single F1 WxR individual who ran 24 trials (mean ± SD: R, 33 ± 0.7; W, 33 ± 0.2; F1 RxW, 32.9 ± 0.6; F1 WxR, 32.7 ± 1.7). Parental and F2 females ran between 22 and 30 pre-training trials (mean ± SD: R, 29.9 ± 0.27; W, 29.5 ± 1.08; F2 RxW, 29.1 ± 2.24; F2 WxR, 28.8 ± 2.25). This variation in number of pre-training trials was due to lack of participation by the fish in some trials and to the replacement of the eight fish a few days into pre-training. Nine females (R, n=3; W, n=1; F1 RxW, n=3; F1 WxR, n=1; F2 WxR, n=1) did not dislodge the green disc during late pre-training stages and were excluded from the experiment. A total of 185 females (R, n=40; W, n = 40; F1 RxW, n=27; F1 WxR, n=29; F2 RxW, n=25; F2 WxR, n=24) successfully retrieved the food reward by dislodging the disc in all trials of the last two sessions and continued the experiment.

## 1.3 Learning assays - males

During pre-training, male fish (n=84) were trained to swim through a hole in a partition to access the experimental chamber and obtain a food reward. Two males (W, n=1; F2 WxR, n=1) did not feed during early pre-training stages and were replaced by new fish. In total, males ran between 23 and 34 pre-training trials (mean ± SD: R, 29.9 ± 0.27; W, 29.6 ± 1.91; F1 RxW, 29.9 ± 1.38; F1 WxR, 29.8 ± 0.97; F2 RxW, 30.2 ± 1.54; F2 WxR, 29.4 ± 1.93). This variation in number of pre-training trials was due to lack of participation by the fish in some trials and to the replacement of the eight fish a few days into pre-training. One male (F2 WxR) was suddenly found dead in the last day of pre-training, and nine males (R, n=1; F1 RxW, n=3; F1 WxR, n=3; F2 RxW, n=1; F2 WxR, n=1) failed in swimming through a small hole to access food in < 3 min and so were excluded from the experiment. A total of 74 males (R, n=13; W, n = 14; F1 RxW, n=11; F1 WxR, n=11; F2 RxW, n=13; F2 WxR, n=12) successfully swam through the 3mm green hole and ate the reward within 3min in all trials of the last session and continued the experiment.

```
## CLEAR WORKSPACE
rm(list=ls())

## LOAD PACKAGES
library(xlsx)
library(reshape2)
library(dplyr)
library(tidyr)
library(kableExtra)
library(lme4)
library(car)
library(boot)
library(multcomp)
library(ggplot2)
library(effects)
library(ggpubr)
library(ks)
library(misc3d)
library(plot3D)
library(akima)

## FUNCTIONS
## ~~~~~~
## Run external functions for 2D and 3D transgression calculations
source("00-Function_Transgression_2D.R", local = knitr::knit_global())
source("01-Function_Transgression_3D.R", local = knitr::knit_global())
kdePlot <- function(kdeObject = NULL) {
  plot(kdeObject,  add = TRUE, drawpoints = TRUE, pch = 20, col.pt = simHCol, cont = 0, alpha = 0.5)
}
## binomial smooth for individual learning curves
binomial_smooth <- function(...) {
  geom_smooth(method = "glm", se = FALSE, alpha = 0.2, method.args = list(family = "binomial"), ...)
}


## OPTIONS
## ~~~~~~
## Colour scheme
## grey shades for parentals, reds for F1 and blues for F2
treatmentCol <- c("#777777", "#3b3b3b", "#f56e51", "#a22638", "#5a96b9", "#225995") # lighter grey (R), darker grey  (W), orange (RW), dark red (WR), light blue (RW f2), dark blue (WR f2)
simHCol <- "grey70" # light grey

# READ DATA FILES
## ~~~~~~
# brain traits
brainsF1 <- read.csv("BrainData_F1_fem_males.csv", header = TRUE)
brainsF2 <- read.csv("BrainData_F2_fem_males.csv", header = TRUE)
## ~~~~~~
# cognitive traits
# fish info - females
infoFem1 <- read.csv("FishID_F1_females.csv", header = TRUE); infoFem1$TankID <- infoFem1$FishID
infoFem2 <- read.csv("FishID_F2_females.csv", header = TRUE); infoFem2$TankID <- as.factor( paste0("T", infoFem2$TankID) )
infoFem2$Species <- factor(infoFem2$Species, levels = c("R", "W", "RW_F2", "WR_F2"))
# add reversal colour - oposite of AL
infoFem2$ColourRL <- ifelse(infoFem2$ColourAL == "Red", "Yellow", "Red"); infoFem2$ColourRL <- factor(infoFem2$ColourRL)
# merge females
infoFem <- merge(infoFem1, infoFem2, all = TRUE)
infoFem$Species <- recode_factor(infoFem$Species, RW = 'RW_F1', WR  = 'WR_F1')
infoFem$Species <- factor(infoFem$Species, levels = c("R", "W", "RW_F1", "WR_F1", "RW_F2", "WR_F2"))
# fish info - males
infoMale <- read.csv("FishID_F1_F2_males.csv", header = TRUE)
infoMale$Species <- recode_factor(infoMale$Species, f1_RW = 'RW_F1', f1_WR  = 'WR_F1', f2_RW = 'RW_F2', f2_WR  = 'WR_F2')
infoMale$Species <- factor(infoMale$Species, levels = c("R", "W", "RW_F1", "WR_F1", "RW_F2", "WR_F2"))
# add reversal colour - oposite of AL
infoMale$ColourRL <- ifelse(infoMale$ColourAL == "red", "yellow", "red"); infoMale$ColourRL <- factor(infoMale$ColourRL)
# trials to LC
LCFem1 <- read.csv("LC_F1_females.csv", header = TRUE)
LCFem2 <- read.csv("LC_F2_females.csv", header = TRUE)
LCFem <- merge(LCFem1, LCFem2, all = TRUE); LCFem <- merge(LCFem, infoFem)
LCFem2 <- merge(LCFem2, infoFem2)
LCMale <- read.csv("LC_F1_F2_males.csv", header = TRUE); LCMale <- merge(LCMale, infoMale)
# associative learning
assocLFem1 <- read.csv("associativeL_F1_females.csv", header = TRUE); assocLFem1 <- merge(assocLFem1, infoFem, by="FishID")
assocLFem2 <- read.csv("associativeL_F2_females.csv", header = TRUE, 
                       colClasses = c("character", "integer", "integer", "character", "integer"))
assocLFem2$TankID <- as.factor( paste0("T", assocLFem2$TankID) ); assocLFem2$Side <- as.factor(assocLFem2$Side)
assocLFem2 <- merge(assocLFem2, infoFem2, by="TankID")
assocLMale <- read.csv("associativeL_F1_F2_males.csv", header = TRUE); assocLMale <- merge(assocLMale, infoMale)
# reversal learning
reversLFem1 <- read.csv("reversalL_F1_females.csv", header = TRUE); reversLFem1 <- merge(reversLFem1, infoFem, by="FishID")
reversLFem2 <- read.csv("reversalL_F2_females.csv", header = TRUE); reversLFem2$TankID <- as.factor( paste0("T", reversLFem2$TankID) )
reversLFem2 <- merge(reversLFem2, infoFem2, by="TankID")
reversLMale <- read.csv("reversalL_F1_F2_males.csv", header = TRUE); reversLMale <- merge(reversLMale, infoMale)
```

# 2 Experimental details

```
experiment <- read.csv("sample size.csv", header=TRUE) # Add and prepare data
experiment %>%
    kable(col.names = c("Species groups","Males","Females","Total","Males","Females","Total"),
        caption = "Table 1. Number of individual males and females from each species group examined in the different parts of the study. Note that 80 males were re-used in the cognitive tasks and brain morphology analyses.")%>% 
  add_header_above(c(" " = 1, "Brain morphology" = 3, "Cognition" = 3)) %>%
  row_spec(7, bold = TRUE) %>%
  kable_styling(c("hover"), full_width=TRUE)
```

Table 1. Number of individual males and females from each species group examined in the different parts of the study. Note that 80 males were re-used in the cognitive tasks and brain morphology analyses.

|  | Brain morphology | | | Cognition | | |
| --- | --- | --- | --- | --- | --- | --- |
| Species groups | Males | Females | Total | Males | Females | Total |
| Poecilia reticulata (R) | 24 | 24 | 48 | 14 | 43 | 57 |
| P. wingei (W) | 23 | 20 | 43 | 14 | 41 | 55 |
| F1 R×W | 24 | 10 | 34 | 14 | 30 | 44 |
| F1 W×R | 22 | 10 | 32 | 14 | 30 | 44 |
| F2 R×W | 14 | 25 | 39 | 14 | 25 | 39 |
| F2 W×R | 12 | 24 | 36 | 14 | 25 | 39 |
| Total | 119 | 113 | 232 | 84 | 194 | 278 |

# 3 Brain morphology analyses

## 3.1 F1 hybrids

```
## get dataset from selected group
brainData <- eval(parse(text = "brainsF1"))

## Log transformation of brain variables + log transformation and mean center body length
## ~~~~~~
brainData$mcBodyLen <- log10(brainData$BodyLength) - mean(log10(brainData$BodyLength))
brainData$weightTotBr <- log10(brainData$BrainWeight_mg)
brainData$volTotBr <- log10(brainData$TotalBrainVol)
brainData$tel <- log10(brainData$TelVolTot)
brainData$tel_rest <- log10(brainData$BrainVol_minusTel)
brainData$ot <- log10(brainData$OTVolTot)
brainData$ot_rest <- log10(brainData$BrainVol_minusOT)
brainData$cer <- log10(brainData$CerVolAvg)
brainData$cer_rest <- log10(brainData$BrainVol_minusCer)
brainData$dm <- log10(brainData$DMVolAvg)
brainData$dm_rest <- log10(brainData$BrainVol_minusDM)
brainData$ob <- log10(brainData$OBVolTot)
brainData$ob_rest <- log10(brainData$BrainVol_minusOB)
brainData$hyp <- log10(brainData$HypVolTot)
brainData$hyp_rest <- log10(brainData$BrainVol_minusHyp)
```

```
# Sample size for F1 brain morphology analyses
table(brainData$Species, brainData$Sex); print(paste0("Total N = ", length(unique(brainData$FishID))))
```

```
##     
##       F  M
##   R  11 10
##   RW 10 24
##   W  10 10
##   WR 10 22
```

```
## [1] "Total N = 107"
```

### 3.1.1 Differences in relative brain size?

```
relBrain.m1 <- lm(weightTotBr ~ Species + Sex + mcBodyLen
                  + Species*mcBodyLen
                  + Species*Sex
                  + Sex*mcBodyLen
                  , data = brainData)
Anova(relBrain.m1)
```

```
## Anova Table (Type II tests)
## 
## Response: weightTotBr
##                     Sum Sq Df  F value    Pr(>F)    
## Species           0.027364  3   5.2652  0.002115 ** 
## Sex               0.002410  1   1.3909  0.241236    
## mcBodyLen         0.176379  1 101.8117 < 2.2e-16 ***
## Species:mcBodyLen 0.002862  3   0.5507  0.648922    
## Species:Sex       0.003241  3   0.6236  0.601502    
## Sex:mcBodyLen     0.000849  1   0.4900  0.485657    
## Residuals         0.162846 94                       
## ---
## Signif. codes:  0 '***' 0.001 '**' 0.01 '*' 0.05 '.' 0.1 ' ' 1
```

```
# test significance of interaction terms
relBrain.m2 <- lm(weightTotBr ~ Species + Sex + mcBodyLen
                  # + Species*mcBodyLen
                  + Species*Sex
                  + Sex*mcBodyLen
                  , data = brainData)
anova(relBrain.m1, relBrain.m2) ## interaction does not contribute to explain variance
```

```
## Analysis of Variance Table
## 
## Model 1: weightTotBr ~ Species + Sex + mcBodyLen + Species * mcBodyLen + 
##     Species * Sex + Sex * mcBodyLen
## Model 2: weightTotBr ~ Species + Sex + mcBodyLen + Species * Sex + Sex * 
##     mcBodyLen
##   Res.Df     RSS Df Sum of Sq      F Pr(>F)
## 1     94 0.16285                           
## 2     97 0.16571 -3 -0.002862 0.5507 0.6489
```

```
relBrain.m3 <- lm(weightTotBr ~ Species + Sex + mcBodyLen
                  + Species*mcBodyLen
                  # + Species*Sex
                  + Sex*mcBodyLen
                  , data = brainData)
anova(relBrain.m1, relBrain.m3) ## interaction does not contribute to explain variance
```

```
## Analysis of Variance Table
## 
## Model 1: weightTotBr ~ Species + Sex + mcBodyLen + Species * mcBodyLen + 
##     Species * Sex + Sex * mcBodyLen
## Model 2: weightTotBr ~ Species + Sex + mcBodyLen + Species * mcBodyLen + 
##     Sex * mcBodyLen
##   Res.Df     RSS Df  Sum of Sq      F Pr(>F)
## 1     94 0.16285                            
## 2     97 0.16609 -3 -0.0032409 0.6236 0.6015
```

```
relBrain.m4 <- lm(weightTotBr ~ Species + Sex + mcBodyLen
                  + Species*mcBodyLen
                  + Species*Sex
                  # + Sex*mcBodyLen
                  , data = brainData)
anova(relBrain.m1, relBrain.m4) ## interaction does not contribute to explain variance
```

```
## Analysis of Variance Table
## 
## Model 1: weightTotBr ~ Species + Sex + mcBodyLen + Species * mcBodyLen + 
##     Species * Sex + Sex * mcBodyLen
## Model 2: weightTotBr ~ Species + Sex + mcBodyLen + Species * mcBodyLen + 
##     Species * Sex
##   Res.Df     RSS Df   Sum of Sq    F Pr(>F)
## 1     94 0.16285                           
## 2     95 0.16369 -1 -0.00084887 0.49 0.4857
```

```
# ~~~~~~
## FINAL MODEL
relBrain.m <- lm(weightTotBr ~ Species + Sex + mcBodyLen, data = brainData)
Anova(relBrain.m)
```

```
## Anova Table (Type II tests)
## 
## Response: weightTotBr
##             Sum Sq  Df  F value    Pr(>F)    
## Species   0.025426   3   5.0663  0.002611 ** 
## Sex       0.002313   1   1.3824  0.242450    
## mcBodyLen 0.196291   1 117.3360 < 2.2e-16 ***
## Residuals 0.168963 101                       
## ---
## Signif. codes:  0 '***' 0.001 '**' 0.01 '*' 0.05 '.' 0.1 ' ' 1
```

```
summary(relBrain.m)
```

```
## 
## Call:
## lm(formula = weightTotBr ~ Species + Sex + mcBodyLen, data = brainData)
## 
## Residuals:
##       Min        1Q    Median        3Q       Max 
## -0.100130 -0.028732 -0.000245  0.028175  0.087514 
## 
## Coefficients:
##             Estimate Std. Error t value Pr(>|t|)    
## (Intercept)  0.60495    0.01057  57.259  < 2e-16 ***
## SpeciesRW   -0.01612    0.01342  -1.201 0.232506    
## SpeciesW    -0.04983    0.01387  -3.592 0.000508 ***
## SpeciesWR   -0.02730    0.01299  -2.102 0.038049 *  
## SexM        -0.01970    0.01675  -1.176 0.242450    
## mcBodyLen    1.16576    0.10762  10.832  < 2e-16 ***
## ---
## Signif. codes:  0 '***' 0.001 '**' 0.01 '*' 0.05 '.' 0.1 ' ' 1
## 
## Residual standard error: 0.0409 on 101 degrees of freedom
## Multiple R-squared:  0.8508, Adjusted R-squared:  0.8434 
## F-statistic: 115.2 on 5 and 101 DF,  p-value: < 2.2e-16
```

```
## Post-hoc comparison of groups
summary( glht(relBrain.m, mcp(Species = "Tukey")) )
```

```
## 
##   Simultaneous Tests for General Linear Hypotheses
## 
## Multiple Comparisons of Means: Tukey Contrasts
## 
## 
## Fit: lm(formula = weightTotBr ~ Species + Sex + mcBodyLen, data = brainData)
## 
## Linear Hypotheses:
##              Estimate Std. Error t value Pr(>|t|)   
## RW - R == 0  -0.01612    0.01342  -1.201   0.6245   
## W - R == 0   -0.04983    0.01387  -3.592   0.0026 **
## WR - R == 0  -0.02730    0.01299  -2.102   0.1574   
## W - RW == 0  -0.03371    0.01175  -2.869   0.0249 * 
## WR - RW == 0 -0.01118    0.01013  -1.103   0.6855   
## WR - W == 0   0.02252    0.01177   1.914   0.2263   
## ---
## Signif. codes:  0 '***' 0.001 '**' 0.01 '*' 0.05 '.' 0.1 ' ' 1
## (Adjusted p values reported -- single-step method)
```

---

#### Plot relative brain size

```
## Plot brain weight ~ body length by Species
brainData$Sex <- recode_factor(brainData$Sex, F = "Females", M = "Males")
brainData$Species <- recode_factor(brainData$Species, RW = "F1 RxW", WR = "F1 WxR")
brainData$Species <- factor(brainData$Species, levels = c("R", "W", "F1 RxW", "F1 WxR"))
ggplot(brainData, aes(BodyLength, BrainWeight_mg, col = Species, shape = Species, linetype = Species)) +
  geom_point(size = 1.5, stroke = 1.5) +
  geom_smooth(method = "lm", se = TRUE, aes(fill = Species), alpha = 0.2) +
  ylab("Brain mass (mg)") +
  xlab("Body length (mm)") +
  scale_y_log10() + 
  scale_x_log10() +
  scale_linetype_manual(values = c("solid", "solid", "longdash", "dashed")) +
  scale_shape_manual(values = c(1, 2, 16, 17)) +
  scale_colour_manual(values=treatmentCol)+
  scale_fill_manual(values=treatmentCol)+
  facet_wrap(~ Sex) +
  theme_classic(base_size = 11) + theme(
    panel.background = element_blank(),
    panel.border = element_blank(),
    strip.text = element_text(face = "bold", size = rel(1.2)),
    strip.background = element_rect(fill = "white", colour = "white", size = 1),
    axis.text = element_text(colour = "black"),
    axis.title.x = element_text(margin = unit(c(3, 0, 0, 0), "mm")),
    axis.title.y = element_text(margin = unit(c(0, 3, 0, 0), "mm")) )
```

**Figure 3.1.1.1.** Differences in relative brain size between parentals and F1 hybrids for a) females and b) males. Model predictions are plotted as the best fit line. Body length (x axis) and brain mass (y axis) are shown in log10 scales.

---

### 3.1.2 Differences in relative brain region volumes?

```
## Telencephalon
tel.m <- lm(tel ~ Species + Sex + tel_rest
            # + Species*Sex 
            # + Species*tel_rest 
            # + Sex*tel_rest 
            , data = brainData)
Anova(tel.m)
```

```
## Anova Table (Type II tests)
## 
## Response: tel
##            Sum Sq  Df  F value Pr(>F)    
## Species   0.01411   3   1.6485 0.1830    
## Sex       0.00277   1   0.9704 0.3269    
## tel_rest  0.38203   1 133.9428 <2e-16 ***
## Residuals 0.28807 101                    
## ---
## Signif. codes:  0 '***' 0.001 '**' 0.01 '*' 0.05 '.' 0.1 ' ' 1
```

```
summary(tel.m)
```

```
## 
## Call:
## lm(formula = tel ~ Species + Sex + tel_rest, data = brainData)
## 
## Residuals:
##       Min        1Q    Median        3Q       Max 
## -0.150783 -0.036096 -0.003897  0.029723  0.137600 
## 
## Coefficients:
##               Estimate Std. Error t value Pr(>|t|)    
## (Intercept)   -0.73813    0.04140 -17.830   <2e-16 ***
## SpeciesW       0.01961    0.01673   1.172   0.2440    
## SpeciesF1 RxW  0.02855    0.01546   1.847   0.0677 .  
## SpeciesF1 WxR  0.03234    0.01530   2.113   0.0370 *  
## SexMales      -0.01707    0.01733  -0.985   0.3269    
## tel_rest       1.00508    0.08684  11.573   <2e-16 ***
## ---
## Signif. codes:  0 '***' 0.001 '**' 0.01 '*' 0.05 '.' 0.1 ' ' 1
## 
## Residual standard error: 0.05341 on 101 degrees of freedom
## Multiple R-squared:  0.7958, Adjusted R-squared:  0.7857 
## F-statistic: 78.73 on 5 and 101 DF,  p-value: < 2.2e-16
```

```
## Optic tectum
ot.m <- lm(ot ~ Species + Sex + ot_rest
           # + Species*Sex 
           # + Species*ot_rest 
           # + Sex*ot_rest 
           , data = brainData)
Anova(ot.m)
```

```
## Anova Table (Type II tests)
## 
## Response: ot
##            Sum Sq  Df  F value    Pr(>F)    
## Species   0.00082   3   0.1284  0.943028    
## Sex       0.01870   1   8.7871  0.003785 ** 
## ot_rest   0.22874   1 107.4864 < 2.2e-16 ***
## Residuals 0.21494 101                       
## ---
## Signif. codes:  0 '***' 0.001 '**' 0.01 '*' 0.05 '.' 0.1 ' ' 1
```

```
summary(ot.m)
```

```
## 
## Call:
## lm(formula = ot ~ Species + Sex + ot_rest, data = brainData)
## 
## Residuals:
##       Min        1Q    Median        3Q       Max 
## -0.108313 -0.023299  0.000661  0.028773  0.125119 
## 
## Coefficients:
##                 Estimate Std. Error t value Pr(>|t|)    
## (Intercept)    0.0059317  0.0209161   0.284  0.77730    
## SpeciesW       0.0008476  0.0144722   0.059  0.95341    
## SpeciesF1 RxW  0.0062038  0.0134364   0.462  0.64528    
## SpeciesF1 WxR -0.0002031  0.0132912  -0.015  0.98784    
## SexMales       0.0517615  0.0174616   2.964  0.00378 ** 
## ot_rest        0.7131989  0.0687914  10.368  < 2e-16 ***
## ---
## Signif. codes:  0 '***' 0.001 '**' 0.01 '*' 0.05 '.' 0.1 ' ' 1
## 
## Residual standard error: 0.04613 on 101 degrees of freedom
## Multiple R-squared:  0.6916, Adjusted R-squared:  0.6763 
## F-statistic: 45.29 on 5 and 101 DF,  p-value: < 2.2e-16
```

```
## Cerebellum
cer.m <- lm(cer ~ Species + Sex + cer_rest
            # + Species*Sex 
            # + Species*cer_rest 
            # + Sex*cer_rest 
            , data = brainData)
Anova(cer.m)
```

```
## Anova Table (Type II tests)
## 
## Response: cer
##            Sum Sq  Df F value    Pr(>F)    
## Species   0.02444   3  1.7221    0.1672    
## Sex       0.22026   1 46.5574 6.702e-10 ***
## cer_rest  0.25667   1 54.2535 4.899e-11 ***
## Residuals 0.47782 101                      
## ---
## Signif. codes:  0 '***' 0.001 '**' 0.01 '*' 0.05 '.' 0.1 ' ' 1
```

```
summary(cer.m)
```

```
## 
## Call:
## lm(formula = cer ~ Species + Sex + cer_rest, data = brainData)
## 
## Residuals:
##       Min        1Q    Median        3Q       Max 
## -0.182679 -0.040780 -0.001046  0.043929  0.190365 
## 
## Coefficients:
##               Estimate Std. Error t value Pr(>|t|)    
## (Intercept)   -0.99590    0.05642 -17.652  < 2e-16 ***
## SpeciesW       0.04714    0.02155   2.187   0.0310 *  
## SpeciesF1 RxW  0.03379    0.01997   1.692   0.0936 .  
## SpeciesF1 WxR  0.03047    0.01975   1.542   0.1261    
## SexMales      -0.14737    0.02160  -6.823  6.7e-10 ***
## cer_rest       0.80706    0.10957   7.366  4.9e-11 ***
## ---
## Signif. codes:  0 '***' 0.001 '**' 0.01 '*' 0.05 '.' 0.1 ' ' 1
## 
## Residual standard error: 0.06878 on 101 degrees of freedom
## Multiple R-squared:  0.8107, Adjusted R-squared:  0.8013 
## F-statistic: 86.49 on 5 and 101 DF,  p-value: < 2.2e-16
```

```
## Dorsal medulla
dm.m <- lm(dm ~ Species + Sex + dm_rest
           # + Species*Sex 
           # + Species*dm_rest 
           # + Sex*dm_rest 
           , data = brainData)
Anova(dm.m)
```

```
## Anova Table (Type II tests)
## 
## Response: dm
##             Sum Sq  Df  F value    Pr(>F)    
## Species   0.009635   3   1.1596     0.329    
## Sex       0.258011   1  93.1597 5.247e-16 ***
## dm_rest   0.315965   1 114.0852 < 2.2e-16 ***
## Residuals 0.279725 101                       
## ---
## Signif. codes:  0 '***' 0.001 '**' 0.01 '*' 0.05 '.' 0.1 ' ' 1
```

```
summary(dm.m)
```

```
## 
## Call:
## lm(formula = dm ~ Species + Sex + dm_rest, data = brainData)
## 
## Residuals:
##       Min        1Q    Median        3Q       Max 
## -0.141898 -0.031550  0.002464  0.033154  0.147777 
## 
## Coefficients:
##               Estimate Std. Error t value Pr(>|t|)    
## (Intercept)   -0.61759    0.03927 -15.725  < 2e-16 ***
## SpeciesW      -0.02312    0.01654  -1.398   0.1652    
## SpeciesF1 RxW -0.01994    0.01540  -1.295   0.1984    
## SpeciesF1 WxR -0.02732    0.01520  -1.797   0.0753 .  
## SexMales      -0.15213    0.01576  -9.652 5.25e-16 ***
## dm_rest        0.90039    0.08430  10.681  < 2e-16 ***
## ---
## Signif. codes:  0 '***' 0.001 '**' 0.01 '*' 0.05 '.' 0.1 ' ' 1
## 
## Residual standard error: 0.05263 on 101 degrees of freedom
## Multiple R-squared:  0.8874, Adjusted R-squared:  0.8818 
## F-statistic: 159.2 on 5 and 101 DF,  p-value: < 2.2e-16
```

```
## Olfactory bulbs
ob.m <- lm(ob ~ Species + Sex + ob_rest
           # + Species*Sex
           # + Species*ob_rest
           # + Sex*ob_rest
           , data = brainData)
Anova(ob.m)
```

```
## Anova Table (Type II tests)
## 
## Response: ob
##            Sum Sq  Df F value    Pr(>F)    
## Species   0.26282   3  2.8017   0.04373 *  
## Sex       0.03115   1  0.9961   0.32065    
## ob_rest   0.78814   1 25.2042 2.223e-06 ***
## Residuals 3.15828 101                      
## ---
## Signif. codes:  0 '***' 0.001 '**' 0.01 '*' 0.05 '.' 0.1 ' ' 1
```

```
summary(ob.m)
```

```
## 
## Call:
## lm(formula = ob ~ Species + Sex + ob_rest, data = brainData)
## 
## Residuals:
##      Min       1Q   Median       3Q      Max 
## -0.42707 -0.11756  0.00398  0.13229  0.39793 
## 
## Coefficients:
##               Estimate Std. Error t value Pr(>|t|)    
## (Intercept)   -2.30621    0.15512 -14.867  < 2e-16 ***
## SpeciesW       0.06097    0.05547   1.099    0.274    
## SpeciesF1 RxW -0.08476    0.05149  -1.646    0.103    
## SpeciesF1 WxR -0.02114    0.05086  -0.416    0.679    
## SexMales      -0.05741    0.05752  -0.998    0.321    
## ob_rest        1.43147    0.28513   5.020 2.22e-06 ***
## ---
## Signif. codes:  0 '***' 0.001 '**' 0.01 '*' 0.05 '.' 0.1 ' ' 1
## 
## Residual standard error: 0.1768 on 101 degrees of freedom
## Multiple R-squared:  0.4931, Adjusted R-squared:  0.468 
## F-statistic: 19.65 on 5 and 101 DF,  p-value: 1.255e-13
```

```
summary( glht(ob.m, mcp(Species = "Tukey")) )   # Post-hoc comparison of species groups
```

```
## 
##   Simultaneous Tests for General Linear Hypotheses
## 
## Multiple Comparisons of Means: Tukey Contrasts
## 
## 
## Fit: lm(formula = ob ~ Species + Sex + ob_rest, data = brainData)
## 
## Linear Hypotheses:
##                      Estimate Std. Error t value Pr(>|t|)  
## W - R == 0            0.06097    0.05547   1.099   0.6894  
## F1 RxW - R == 0      -0.08476    0.05149  -1.646   0.3562  
## F1 WxR - R == 0      -0.02114    0.05086  -0.416   0.9755  
## F1 RxW - W == 0      -0.14573    0.05108  -2.853   0.0264 *
## F1 WxR - W == 0      -0.08211    0.05095  -1.612   0.3751  
## F1 WxR - F1 RxW == 0  0.06362    0.04388   1.450   0.4699  
## ---
## Signif. codes:  0 '***' 0.001 '**' 0.01 '*' 0.05 '.' 0.1 ' ' 1
## (Adjusted p values reported -- single-step method)
```

```
## Hypothalamus
hyp.m <- lm(hyp ~ Species + Sex + hyp_rest
            # + Species*Sex 
            # + Species*hyp_rest 
            # + Sex*hyp_rest 
            , data = brainData)
Anova(hyp.m)
```

```
## Anova Table (Type II tests)
## 
## Response: hyp
##            Sum Sq  Df F value    Pr(>F)    
## Species   0.00977   3  0.9183   0.43493    
## Sex       0.01536   1  4.3305   0.03996 *  
## hyp_rest  0.23231   1 65.4963 1.356e-12 ***
## Residuals 0.35824 101                      
## ---
## Signif. codes:  0 '***' 0.001 '**' 0.01 '*' 0.05 '.' 0.1 ' ' 1
```

```
summary(hyp.m)
```

```
## 
## Call:
## lm(formula = hyp ~ Species + Sex + hyp_rest, data = brainData)
## 
## Residuals:
##      Min       1Q   Median       3Q      Max 
## -0.17902 -0.03038  0.00150  0.03810  0.13203 
## 
## Coefficients:
##                Estimate Std. Error t value Pr(>|t|)    
## (Intercept)   -0.751060   0.046320 -16.215  < 2e-16 ***
## SpeciesW      -0.013907   0.018699  -0.744    0.459    
## SpeciesF1 RxW  0.013365   0.017301   0.773    0.442    
## SpeciesF1 WxR  0.008445   0.017117   0.493    0.623    
## SexMales      -0.039774   0.019113  -2.081    0.040 *  
## hyp_rest       0.757118   0.093553   8.093 1.36e-12 ***
## ---
## Signif. codes:  0 '***' 0.001 '**' 0.01 '*' 0.05 '.' 0.1 ' ' 1
## 
## Residual standard error: 0.05956 on 101 degrees of freedom
## Multiple R-squared:  0.7039, Adjusted R-squared:  0.6892 
## F-statistic: 48.01 on 5 and 101 DF,  p-value: < 2.2e-16
```

---

#### Plot relative brain regions

```
knitr::include_graphics("F1-Females_legend.jpg")
```

```
## Females
## tel
ggplot(brainData  %>% 
         filter(Sex == "Females"), 
       aes(x = BrainVol_minusTel, y = TelVolTot, col = Species, shape = Species, linetype=Species)) +
  geom_point(size = 2, stroke = 1.8) +
  geom_smooth(method = "lm", se = TRUE, aes(fill = Species), alpha = 0.2) +
  ylab(expression(Volume ~(mm^3))) +
  xlab(expression('Rest of the brain' ~(mm^3))) +
  ggtitle("TEL volume") +
  scale_y_log10() + 
  scale_x_log10() +
  scale_linetype_manual(values = c("solid", "solid", "longdash", "dashed")) +
  scale_shape_manual(values = c(1, 2, 16, 17)) +
  scale_colour_manual(values=treatmentCol)+
  scale_fill_manual(values=treatmentCol)+
  theme_classic(base_size = 18) + theme(
    panel.background = element_blank(),
    panel.border = element_blank(),
    legend.position="none",
    axis.text = element_text(colour = "black"),
    axis.title.x = element_text(margin = unit(c(3, 0, 0, 0), "mm")),
    axis.title.y = element_text(margin = unit(c(0, 3, 0, 0), "mm")) )
## ot
ggplot(brainData  %>% 
         filter(Sex == "Females"), 
       aes(x = BrainVol_minusOT, y = OTVolTot, col = Species, shape = Species, linetype=Species)) +
  geom_point(size = 2, stroke = 1.8) +
  geom_smooth(method = "lm", se = TRUE, aes(fill = Species), alpha = 0.2) +
  ylab(expression(Volume ~(mm^3))) +
  xlab(expression('Rest of the brain' ~(mm^3))) +
  ggtitle("OT volume") +
  scale_y_log10() + 
  scale_x_log10() +
  scale_linetype_manual(values = c("solid", "solid", "longdash", "dashed")) +
  scale_shape_manual(values = c(1, 2, 16, 17)) +
  scale_colour_manual(values=treatmentCol)+
  scale_fill_manual(values=treatmentCol)+
  theme_classic(base_size = 18) + theme(
    panel.background = element_blank(),
    panel.border = element_blank(),
    legend.position="none",
    axis.text = element_text(colour = "black"),
    axis.title.x = element_text(margin = unit(c(3, 0, 0, 0), "mm")),
    axis.title.y = element_text(margin = unit(c(0, 3, 0, 0), "mm")) )
## cer
ggplot(brainData  %>% 
         filter(Sex == "Females"), 
       aes(x = BrainVol_minusCer, y = CerVolAvg, col = Species, shape = Species, linetype=Species)) +
  geom_point(size = 2, stroke = 1.8) +
  geom_smooth(method = "lm", se = TRUE, aes(fill = Species), alpha = 0.2) +
  ylab(expression(Volume ~(mm^3))) +
  xlab(expression('Rest of the brain' ~(mm^3))) +
  ggtitle("CER volume") +
  scale_y_log10() + 
  scale_x_log10() +
  scale_linetype_manual(values = c("solid", "solid", "longdash", "dashed")) +
  scale_shape_manual(values = c(1, 2, 16, 17)) +
  scale_colour_manual(values=treatmentCol)+
  scale_fill_manual(values=treatmentCol)+
  theme_classic(base_size = 18) + theme(
    panel.background = element_blank(),
    panel.border = element_blank(),
    legend.position="none",
    axis.text = element_text(colour = "black"),
    axis.title.x = element_text(margin = unit(c(3, 0, 0, 0), "mm")),
    axis.title.y = element_text(margin = unit(c(0, 3, 0, 0), "mm")) )
## Hyp
ggplot(brainData  %>% 
         filter(Sex == "Females"), 
       aes(x = BrainVol_minusHyp, y = HypVolTot, col = Species, shape = Species, linetype = Species)) +
  geom_point(size = 2, stroke = 1.8) +
  geom_smooth(method = "lm", se = TRUE, aes(fill = Species), alpha = 0.2) +
  ylab(expression(Volume ~(mm^3))) +
  xlab(expression('Rest of the brain' ~(mm^3))) +
  ggtitle("HYP volume") +
  scale_y_log10() + 
  scale_x_log10() +
  scale_linetype_manual(values = c("solid", "solid", "longdash", "dashed")) +
  scale_shape_manual(values = c(1, 2, 16, 17)) +
  scale_colour_manual(values=treatmentCol)+
  scale_fill_manual(values=treatmentCol)+
  theme_classic(base_size = 18) + theme(
    panel.background = element_blank(),
    panel.border = element_blank(),
    legend.position="none",
    axis.text = element_text(colour = "black"),
    axis.title.x = element_text(margin = unit(c(3, 0, 0, 0), "mm")),
    axis.title.y = element_text(margin = unit(c(0, 3, 0, 0), "mm")) )
## DM
ggplot(brainData  %>% 
         filter(Sex == "Females"), 
       aes(x = BrainVol_minusDM, y = DMVolAvg, col = Species, shape = Species, linetype = Species)) +
  geom_point(size = 2, stroke = 1.8) +
  geom_smooth(method = "lm", se = TRUE, aes(fill = Species), alpha = 0.2) +
  ylab(expression(Volume ~(mm^3))) +
  xlab(expression('Rest of the brain' ~(mm^3))) +
  ggtitle("DM volume") +
  scale_y_log10() + 
  scale_x_log10() +
  scale_linetype_manual(values = c("solid", "solid", "longdash", "dashed")) +
  scale_shape_manual(values = c(1, 2, 16, 17)) +
  scale_colour_manual(values=treatmentCol)+
  scale_fill_manual(values=treatmentCol)+
  theme_classic(base_size = 18) + theme(
    panel.background = element_blank(),
    panel.border = element_blank(),
    legend.position="none",
    axis.text = element_text(colour = "black"),
    axis.title.x = element_text(margin = unit(c(3, 0, 0, 0), "mm")),
    axis.title.y = element_text(margin = unit(c(0, 3, 0, 0), "mm")) )
## ob
ggplot(brainData  %>% 
         filter(Sex == "Females"), 
       aes(x = BrainVol_minusOB, y = OBVolTot, col = Species, shape = Species, linetype = Species)) +
  geom_point(size = 2, stroke = 1.8) +
  geom_smooth(method = "lm", se = TRUE, aes(fill = Species), alpha = 0.2) +
  ylab(expression(Volume ~(mm^3))) +
  xlab(expression('Rest of the brain' ~(mm^3))) +
  ggtitle("OB volume") +
  scale_y_log10() + 
  scale_x_log10() +
  scale_linetype_manual(values = c("solid", "solid", "longdash", "dashed")) +
  scale_shape_manual(values = c(1, 2, 16, 17)) +
  scale_colour_manual(values=treatmentCol)+
  scale_fill_manual(values=treatmentCol)+
  theme_classic(base_size = 18) + theme(
    panel.background = element_blank(),
    panel.border = element_blank(),
    legend.position="none",
    axis.text = element_text(colour = "black"),
    axis.title.x = element_text(margin = unit(c(3, 0, 0, 0), "mm")),
    axis.title.y = element_text(margin = unit(c(0, 3, 0, 0), "mm")) )
```

**Figure 3.1.2.1a.** Differences in relative brain region volume between parentals and F1 hybrids for females. Model predictions are plotted as the best fit line. Remainder brain volume (x axis) and brain region volume (y axis) are shown in log10 scales.

```
knitr::include_graphics("F1-Males_legend.jpg")
```

```
## Males
## tel
ggplot(brainData  %>% 
         filter(Sex == "Males"), 
       aes(x = BrainVol_minusTel, y = TelVolTot, col = Species, shape = Species, linetype = Species)) +
  geom_point(size = 2, stroke = 1.8) +
  geom_smooth(method = "lm", se = TRUE, aes(fill = Species), alpha = 0.2) +
  ylab(expression(Volume ~(mm^3))) +
  xlab(expression('Rest of the brain' ~(mm^3))) +
  ggtitle("TEL volume") +
  scale_linetype_manual(values = c("solid", "solid", "longdash", "dashed")) +
  scale_shape_manual(values = c(1, 2, 16, 17)) +
  scale_colour_manual(values=treatmentCol)+
  scale_fill_manual(values=treatmentCol)+
  scale_y_log10() + 
  scale_x_log10() +
  theme_classic(base_size = 18) + theme(
    panel.background = element_blank(),
    panel.border = element_blank(),
    legend.position="none",
    axis.text = element_text(colour = "black"),
    axis.title.x = element_text(margin = unit(c(3, 0, 0, 0), "mm")),
    axis.title.y = element_text(margin = unit(c(0, 3, 0, 0), "mm")) )
## ot
ggplot(brainData  %>% 
         filter(Sex == "Males"), 
       aes(x = BrainVol_minusOT, y = OTVolTot, col = Species, shape = Species, linetype = Species)) +
  geom_point(size = 2, stroke = 1.8) +
  geom_smooth(method = "lm", se = TRUE, aes(fill = Species), alpha = 0.2) +
  ylab(expression(Volume ~(mm^3))) +
  xlab(expression('Rest of the brain' ~(mm^3))) +
  ggtitle("OT volume") +
  scale_y_log10() + 
  scale_x_log10() +
  scale_linetype_manual(values = c("solid", "solid", "longdash", "dashed")) +
  scale_shape_manual(values = c(1, 2, 16, 17)) +
  scale_colour_manual(values=treatmentCol)+
  scale_fill_manual(values=treatmentCol)+
  theme_classic(base_size = 18) + theme(
    panel.background = element_blank(),
    panel.border = element_blank(),
    legend.position="none",
    axis.text = element_text(colour = "black"),
    axis.title.x = element_text(margin = unit(c(3, 0, 0, 0), "mm")),
    axis.title.y = element_text(margin = unit(c(0, 3, 0, 0), "mm")) )
## cer
ggplot(brainData  %>% 
         filter(Sex == "Males"), 
       aes(x = BrainVol_minusCer, y = CerVolAvg, col = Species, shape = Species, linetype = Species)) +
  geom_point(size = 2, stroke = 1.8) +
  geom_smooth(method = "lm", se = TRUE, aes(fill = Species), alpha = 0.2) +
  ylab(expression(Volume ~(mm^3))) +
  xlab(expression('Rest of the brain' ~(mm^3))) +
  ggtitle("CER volume") +
  scale_y_log10() + 
  scale_x_log10() +
  scale_linetype_manual(values = c("solid", "solid", "longdash", "dashed")) +
  scale_shape_manual(values = c(1, 2, 16, 17)) +
  scale_colour_manual(values=treatmentCol)+
  scale_fill_manual(values=treatmentCol)+
  theme_classic(base_size = 18) + theme(
    panel.background = element_blank(),
    panel.border = element_blank(),
    legend.position="none",
    axis.text = element_text(colour = "black"),
    axis.title.x = element_text(margin = unit(c(3, 0, 0, 0), "mm")),
    axis.title.y = element_text(margin = unit(c(0, 3, 0, 0), "mm")) )
## Hyp
ggplot(brainData  %>% 
         filter(Sex == "Males"), 
       aes(x = BrainVol_minusHyp, y = HypVolTot, col = Species, shape = Species, linetype = Species)) +
  geom_point(size = 2, stroke = 1.8) +
  geom_smooth(method = "lm", se = TRUE, aes(fill = Species), alpha = 0.2) +
  ylab(expression(Volume ~(mm^3))) +
  xlab(expression('Rest of the brain' ~(mm^3))) +
  ggtitle("HYP volume") +
  scale_y_log10() + 
  scale_x_log10() +
  scale_linetype_manual(values = c("solid", "solid", "longdash", "dashed")) +
  scale_shape_manual(values = c(1, 2, 16, 17)) +
  scale_colour_manual(values=treatmentCol)+
  scale_fill_manual(values=treatmentCol)+
  theme_classic(base_size = 18) + theme(
    panel.background = element_blank(),
    panel.border = element_blank(),
    legend.position="none",
    axis.text = element_text(colour = "black"),
    axis.title.x = element_text(margin = unit(c(3, 0, 0, 0), "mm")),
    axis.title.y = element_text(margin = unit(c(0, 3, 0, 0), "mm")) )
## DM
ggplot(brainData  %>% 
         filter(Sex == "Males"), 
       aes(x = BrainVol_minusDM, y = DMVolAvg, col = Species, shape = Species, linetype = Species)) +
  geom_point(size = 2, stroke = 1.8) +
  geom_smooth(method = "lm", se = TRUE, aes(fill = Species), alpha = 0.2) +
  ylab(expression(Volume ~(mm^3))) +
  xlab(expression('Rest of the brain' ~(mm^3))) +
  ggtitle("DM volume") +
  scale_y_log10() + 
  scale_x_log10() +
  scale_linetype_manual(values = c("solid", "solid", "longdash", "dashed")) +
  scale_shape_manual(values = c(1, 2, 16, 17)) +
  scale_colour_manual(values=treatmentCol)+
  scale_fill_manual(values=treatmentCol)+
  theme_classic(base_size = 18) + theme(
    panel.background = element_blank(),
    panel.border = element_blank(),
    legend.position="none",
    axis.text = element_text(colour = "black"),
    axis.title.x = element_text(margin = unit(c(3, 0, 0, 0), "mm")),
    axis.title.y = element_text(margin = unit(c(0, 3, 0, 0), "mm")) )
## ob
ggplot(brainData  %>% 
         filter(Sex == "Males"), 
       aes(x = BrainVol_minusOB, y = OBVolTot, col = Species, shape = Species, linetype = Species)) +
  geom_point(size = 2, stroke = 1.8) +
  geom_smooth(method = "lm", se = TRUE, aes(fill = Species), alpha = 0.2) +
  ylab(expression(Volume ~(mm^3))) +
  xlab(expression('Rest of the brain' ~(mm^3))) +
  ggtitle("OB volume") +
  scale_y_log10() + 
  scale_x_log10() +
  scale_linetype_manual(values = c("solid", "solid", "longdash", "dashed")) +
  scale_shape_manual(values = c(1, 2, 16, 17)) +
  scale_colour_manual(values=treatmentCol)+
  scale_fill_manual(values=treatmentCol)+
  theme_classic(base_size = 18) + theme(
    panel.background = element_blank(),
    panel.border = element_blank(),
    legend.position="none",
    axis.text = element_text(colour = "black"),
    axis.title.x = element_text(margin = unit(c(3, 0, 0, 0), "mm")),
    axis.title.y = element_text(margin = unit(c(0, 3, 0, 0), "mm")) )
```

**Figure 3.1.2.1b.** Differences in relative brain region volume between parentals and F1 hybrids for males. Model predictions are plotted as the best fit line. Body length (x axis) and brain mass (y axis) are shown in log10 scales.

---

## 3.2 F2 hybrids

```
## get dataset from selected group
brainData <- eval(parse(text = "brainsF2"))

## Log transformation of brain variables + log transformation and mean center body length
## ~~~~~~
brainData$mcBodyLen <- log10(brainData$BodyLength) - mean(log10(brainData$BodyLength))
brainData$weightTotBr <- log10(brainData$BrainWeight_mg)
brainData$volTotBr <- log10(brainData$TotalBrainVol)
brainData$tel <- log10(brainData$TelVolTot)
brainData$tel_rest <- log10(brainData$BrainVol_minusTel)
brainData$ot <- log10(brainData$OTVolTot)
brainData$ot_rest <- log10(brainData$BrainVol_minusOT)
brainData$cer <- log10(brainData$CerVolAvg)
brainData$cer_rest <- log10(brainData$BrainVol_minusCer)
brainData$dm <- log10(brainData$DMVolAvg)
brainData$dm_rest <- log10(brainData$BrainVol_minusDM)
brainData$ob <- log10(brainData$OBVolTot)
brainData$ob_rest <- log10(brainData$BrainVol_minusOB)
brainData$hyp <- log10(brainData$HypVolTot)
brainData$hyp_rest <- log10(brainData$BrainVol_minusHyp)
```

```
# Sample size for F2 brain morphology analyses
table(brainData$Species, brainData$Sex); print(paste0("Total N = ", length(unique(brainData$FishID))))
```

```
##        
##          F  M
##   R     13 14
##   RW_F2 25 14
##   W     10 13
##   WR_F2 24 12
```

```
## [1] "Total N = 125"
```

### 3.2.1 Differences in relative brain size?

```
relBrain.m1 <- lm(weightTotBr ~ Species + Sex + mcBodyLen
                  + Species*mcBodyLen
                  + Species*Sex
                  + Sex*mcBodyLen
                  , data = brainData)
Anova(relBrain.m1)
```

```
## Anova Table (Type II tests)
## 
## Response: weightTotBr
##                     Sum Sq  Df F value    Pr(>F)    
## Species           0.006727   3  1.4288 0.2381709    
## Sex               0.005339   1  3.4021 0.0677566 .  
## mcBodyLen         0.110906   1 70.6719 1.511e-13 ***
## Species:mcBodyLen 0.003625   3  0.7699 0.5132128    
## Species:Sex       0.004879   3  1.0363 0.3794376    
## Sex:mcBodyLen     0.022793   1 14.5245 0.0002266 ***
## Residuals         0.175763 112                      
## ---
## Signif. codes:  0 '***' 0.001 '**' 0.01 '*' 0.05 '.' 0.1 ' ' 1
```

```
# test significance of interaction terms
relBrain.m2 <- lm(weightTotBr ~ Species + Sex + mcBodyLen
                  # + Species*mcBodyLen
                  + Species*Sex
                  + Sex*mcBodyLen
                  , data = brainData)
anova(relBrain.m1, relBrain.m2) ## interaction does not contribute to explain variance
```

```
## Analysis of Variance Table
## 
## Model 1: weightTotBr ~ Species + Sex + mcBodyLen + Species * mcBodyLen + 
##     Species * Sex + Sex * mcBodyLen
## Model 2: weightTotBr ~ Species + Sex + mcBodyLen + Species * Sex + Sex * 
##     mcBodyLen
##   Res.Df     RSS Df  Sum of Sq      F Pr(>F)
## 1    112 0.17576                            
## 2    115 0.17939 -3 -0.0036247 0.7699 0.5132
```

```
relBrain.m3 <- lm(weightTotBr ~ Species + Sex + mcBodyLen
                  + Species*mcBodyLen
                  # + Species*Sex
                  + Sex*mcBodyLen
                  , data = brainData)
anova(relBrain.m1, relBrain.m3) ## interaction does not contribute to explain variance
```

```
## Analysis of Variance Table
## 
## Model 1: weightTotBr ~ Species + Sex + mcBodyLen + Species * mcBodyLen + 
##     Species * Sex + Sex * mcBodyLen
## Model 2: weightTotBr ~ Species + Sex + mcBodyLen + Species * mcBodyLen + 
##     Sex * mcBodyLen
##   Res.Df     RSS Df  Sum of Sq      F Pr(>F)
## 1    112 0.17576                            
## 2    115 0.18064 -3 -0.0048788 1.0363 0.3794
```

```
relBrain.m4 <- lm(weightTotBr ~ Species + Sex + mcBodyLen
                  + Species*mcBodyLen
                  + Species*Sex
                  # + Sex*mcBodyLen
                  , data = brainData)
anova(relBrain.m1, relBrain.m4) ## ** interaction contributes to explain variance so we keep it **
```

```
## Analysis of Variance Table
## 
## Model 1: weightTotBr ~ Species + Sex + mcBodyLen + Species * mcBodyLen + 
##     Species * Sex + Sex * mcBodyLen
## Model 2: weightTotBr ~ Species + Sex + mcBodyLen + Species * mcBodyLen + 
##     Species * Sex
##   Res.Df     RSS Df Sum of Sq      F    Pr(>F)    
## 1    112 0.17576                                  
## 2    113 0.19856 -1 -0.022794 14.524 0.0002266 ***
## ---
## Signif. codes:  0 '***' 0.001 '**' 0.01 '*' 0.05 '.' 0.1 ' ' 1
```

```
# ~~~~~~
## FINAL MODEL
relBrain.m <- lm(weightTotBr ~ Species + Sex + mcBodyLen + Sex*mcBodyLen, data = brainData)
Anova(relBrain.m)
```

```
## Anova Table (Type II tests)
## 
## Response: weightTotBr
##                 Sum Sq  Df F value    Pr(>F)    
## Species       0.006727   3  1.4542 0.2306615    
## Sex           0.004015   1  2.6041 0.1092621    
## mcBodyLen     0.117622   1 76.2838 1.932e-14 ***
## Sex:mcBodyLen 0.019877   1 12.8912 0.0004822 ***
## Residuals     0.181945 118                      
## ---
## Signif. codes:  0 '***' 0.001 '**' 0.01 '*' 0.05 '.' 0.1 ' ' 1
```

```
summary(relBrain.m)
```

```
## 
## Call:
## lm(formula = weightTotBr ~ Species + Sex + mcBodyLen + Sex * 
##     mcBodyLen, data = brainData)
## 
## Residuals:
##       Min        1Q    Median        3Q       Max 
## -0.122565 -0.024339  0.004366  0.020181  0.162818 
## 
## Coefficients:
##                 Estimate Std. Error t value Pr(>|t|)    
## (Intercept)     0.568361   0.009846  57.727  < 2e-16 ***
## SpeciesRW_F2   -0.010432   0.010574  -0.987 0.325893    
## SpeciesW       -0.019161   0.012595  -1.521 0.130849    
## SpeciesWR_F2   -0.020741   0.010617  -1.953 0.053131 .  
## SexM            0.048745   0.017666   2.759 0.006716 ** 
## mcBodyLen       0.769411   0.152575   5.043 1.68e-06 ***
## SexM:mcBodyLen  0.875354   0.243802   3.590 0.000482 ***
## ---
## Signif. codes:  0 '***' 0.001 '**' 0.01 '*' 0.05 '.' 0.1 ' ' 1
## 
## Residual standard error: 0.03927 on 118 degrees of freedom
## Multiple R-squared:  0.7207, Adjusted R-squared:  0.7065 
## F-statistic: 50.76 on 6 and 118 DF,  p-value: < 2.2e-16
```

```
## Post-hoc comparison of groups
summary( glht(relBrain.m, mcp(Species = "Tukey")) )
```

```
## 
##   Simultaneous Tests for General Linear Hypotheses
## 
## Multiple Comparisons of Means: Tukey Contrasts
## 
## 
## Fit: lm(formula = weightTotBr ~ Species + Sex + mcBodyLen + Sex * 
##     mcBodyLen, data = brainData)
## 
## Linear Hypotheses:
##                     Estimate Std. Error t value Pr(>|t|)
## RW_F2 - R == 0     -0.010432   0.010574  -0.987    0.755
## W - R == 0         -0.019161   0.012595  -1.521    0.425
## WR_F2 - R == 0     -0.020741   0.010617  -1.953    0.209
## W - RW_F2 == 0     -0.008729   0.010652  -0.819    0.844
## WR_F2 - RW_F2 == 0 -0.010309   0.009260  -1.113    0.679
## WR_F2 - W == 0     -0.001580   0.011098  -0.142    0.999
## (Adjusted p values reported -- single-step method)
```

---

#### Plot relative brain size

```
## Plot brain weight ~ body length by Species
brainData$Sex <- recode_factor(brainData$Sex, F = "Females", M = "Males")
brainData$Species <- recode_factor(brainData$Species, RW_F2 = "F2 RxW", WR_F2 = "F2 WxR")
brainData$Species <- factor(brainData$Species, levels = c("R", "W", "F2 RxW", "F2 WxR"))
ggplot(brainData, aes(BodyLength, BrainWeight_mg, col = Species, shape = Species, linetype = Species)) +
  geom_point(size = 1.5, stroke = 1.5) +
  geom_smooth(method = "lm", se = TRUE, aes(fill = Species), alpha = 0.2) +
  ylab("Brain mass (mg)") +
  xlab("Body length (mm)") +
  scale_y_log10() + 
  scale_x_log10() +
  scale_linetype_manual(values = c("solid", "solid", "longdash", "dashed")) +
  scale_shape_manual(values = c(1, 2, 16, 17)) +
  scale_colour_manual(values=treatmentCol[c(1,2,5,6)])+
  scale_fill_manual(values=treatmentCol[c(1,2,5,6)])+
  facet_wrap(~ Sex) +
  theme_classic(base_size = 11) + theme(
    panel.background = element_blank(),
    panel.border = element_blank(),
    strip.text = element_text(face = "bold", size = rel(1.2)),
    strip.background = element_rect(fill = "white", colour = "white", size = 1),
    axis.text = element_text(colour = "black"),
    axis.title.x = element_text(margin = unit(c(3, 0, 0, 0), "mm")),
    axis.title.y = element_text(margin = unit(c(0, 3, 0, 0), "mm")) )
```

**Figure 3.2.1.1.** Differences in relative brain size between parentals and F2 hybrids for a) females and b) males. Model predictions are plotted as the best fit line. Body length (x axis) and brain mass (y axis) are shown in log10 scales.

---

### 3.2.2 Differences in relative brain region volumes?

```
## Telencephalon
tel.m <- lm(tel ~ Species + Sex + tel_rest
            # + Species*Sex
            # + Species*tel_rest
            + Sex*tel_rest
            , data = brainData)
Anova(tel.m)
```

```
## Anova Table (Type II tests)
## 
## Response: tel
##               Sum Sq  Df  F value    Pr(>F)    
## Species      0.01881   3   3.4352  0.019243 *  
## Sex          0.00411   1   2.2521  0.136103    
## tel_rest     0.40167   1 220.0782 < 2.2e-16 ***
## Sex:tel_rest 0.01320   1   7.2315  0.008201 ** 
## Residuals    0.21537 118                       
## ---
## Signif. codes:  0 '***' 0.001 '**' 0.01 '*' 0.05 '.' 0.1 ' ' 1
```

```
summary(tel.m)
```

```
## 
## Call:
## lm(formula = tel ~ Species + Sex + tel_rest + Sex * tel_rest, 
##     data = brainData)
## 
## Residuals:
##      Min       1Q   Median       3Q      Max 
## -0.13404 -0.02569 -0.00002  0.02562  0.11415 
## 
## Coefficients:
##                   Estimate Std. Error t value Pr(>|t|)    
## (Intercept)       -0.68659    0.04430 -15.499  < 2e-16 ***
## SpeciesW           0.02984    0.01239   2.408  0.01760 *  
## SpeciesF2 RxW      0.01832    0.01098   1.668  0.09789 .  
## SpeciesF2 WxR      0.03285    0.01098   2.992  0.00337 ** 
## SexMales          -0.11994    0.05491  -2.184  0.03091 *  
## tel_rest           0.88841    0.09900   8.974  5.3e-15 ***
## SexMales:tel_rest  0.40162    0.14935   2.689  0.00820 ** 
## ---
## Signif. codes:  0 '***' 0.001 '**' 0.01 '*' 0.05 '.' 0.1 ' ' 1
## 
## Residual standard error: 0.04272 on 118 degrees of freedom
## Multiple R-squared:  0.8554, Adjusted R-squared:  0.848 
## F-statistic: 116.3 on 6 and 118 DF,  p-value: < 2.2e-16
```

```
summary( glht(tel.m, mcp(Species = "Tukey")) )   # Post-hoc comparison of species groups
```

```
## 
##   Simultaneous Tests for General Linear Hypotheses
## 
## Multiple Comparisons of Means: Tukey Contrasts
## 
## 
## Fit: lm(formula = tel ~ Species + Sex + tel_rest + Sex * tel_rest, 
##     data = brainData)
## 
## Linear Hypotheses:
##                      Estimate Std. Error t value Pr(>|t|)  
## W - R == 0            0.02984    0.01239   2.408   0.0810 .
## F2 RxW - R == 0       0.01832    0.01098   1.668   0.3436  
## F2 WxR - R == 0       0.03285    0.01098   2.992   0.0175 *
## F2 RxW - W == 0      -0.01151    0.01136  -1.014   0.7405  
## F2 WxR - W == 0       0.00301    0.01178   0.256   0.9941  
## F2 WxR - F2 RxW == 0  0.01452    0.01006   1.444   0.4731  
## ---
## Signif. codes:  0 '***' 0.001 '**' 0.01 '*' 0.05 '.' 0.1 ' ' 1
## (Adjusted p values reported -- single-step method)
```

```
## Optic tectum
ot.m <- lm(ot ~ Species + Sex + ot_rest
           # + Species*Sex
           # + Species*ot_rest
           # + Sex*ot_rest
           , data = brainData)
Anova(ot.m)
```

```
## Anova Table (Type II tests)
## 
## Response: ot
##             Sum Sq  Df  F value    Pr(>F)    
## Species   0.017031   3   4.6763     0.004 ** 
## Sex       0.020943   1  17.2512 6.192e-05 ***
## ot_rest   0.187639   1 154.5614 < 2.2e-16 ***
## Residuals 0.144467 119                       
## ---
## Signif. codes:  0 '***' 0.001 '**' 0.01 '*' 0.05 '.' 0.1 ' ' 1
```

```
summary(ot.m)
```

```
## 
## Call:
## lm(formula = ot ~ Species + Sex + ot_rest, data = brainData)
## 
## Residuals:
##       Min        1Q    Median        3Q       Max 
## -0.106061 -0.020648 -0.002144  0.018202  0.125074 
## 
## Coefficients:
##                Estimate Std. Error t value Pr(>|t|)    
## (Intercept)   -0.015278   0.015272  -1.000  0.31915    
## SpeciesW      -0.004295   0.009940  -0.432  0.66641    
## SpeciesF2 RxW -0.008714   0.008850  -0.985  0.32683    
## SpeciesF2 WxR -0.030151   0.009022  -3.342  0.00111 ** 
## SexMales       0.056193   0.013529   4.153 6.19e-05 ***
## ot_rest        0.611981   0.049225  12.432  < 2e-16 ***
## ---
## Signif. codes:  0 '***' 0.001 '**' 0.01 '*' 0.05 '.' 0.1 ' ' 1
## 
## Residual standard error: 0.03484 on 119 degrees of freedom
## Multiple R-squared:  0.7545, Adjusted R-squared:  0.7442 
## F-statistic: 73.15 on 5 and 119 DF,  p-value: < 2.2e-16
```

```
summary( glht(ot.m, mcp(Species = "Tukey")) )   # Post-hoc comparison of species groups
```

```
## 
##   Simultaneous Tests for General Linear Hypotheses
## 
## Multiple Comparisons of Means: Tukey Contrasts
## 
## 
## Fit: lm(formula = ot ~ Species + Sex + ot_rest, data = brainData)
## 
## Linear Hypotheses:
##                       Estimate Std. Error t value Pr(>|t|)   
## W - R == 0           -0.004295   0.009940  -0.432  0.97269   
## F2 RxW - R == 0      -0.008714   0.008850  -0.985  0.75749   
## F2 WxR - R == 0      -0.030151   0.009022  -3.342  0.00622 **
## F2 RxW - W == 0      -0.004418   0.009256  -0.477  0.96377   
## F2 WxR - W == 0      -0.025855   0.009421  -2.744  0.03455 * 
## F2 WxR - F2 RxW == 0 -0.021437   0.008055  -2.661  0.04307 * 
## ---
## Signif. codes:  0 '***' 0.001 '**' 0.01 '*' 0.05 '.' 0.1 ' ' 1
## (Adjusted p values reported -- single-step method)
```

```
## Cerebellum
cer.m <- lm(cer ~ Species + Sex + cer_rest
            # + Species*Sex
            # + Species*cer_rest
            # + Sex*cer_rest
            , data = brainData)
Anova(cer.m)
```

```
## Anova Table (Type II tests)
## 
## Response: cer
##           Sum Sq  Df   F value Pr(>F)    
## Species   0.0053   3    0.5821 0.6279    
## Sex       3.7897   1 1256.0604 <2e-16 ***
## cer_rest  0.4020   1  133.2498 <2e-16 ***
## Residuals 0.3590 119                     
## ---
## Signif. codes:  0 '***' 0.001 '**' 0.01 '*' 0.05 '.' 0.1 ' ' 1
```

```
summary(cer.m)
```

```
## 
## Call:
## lm(formula = cer ~ Species + Sex + cer_rest, data = brainData)
## 
## Residuals:
##       Min        1Q    Median        3Q       Max 
## -0.175012 -0.028351  0.004797  0.043198  0.093509 
## 
## Coefficients:
##                Estimate Std. Error t value Pr(>|t|)    
## (Intercept)   -0.670878   0.041202 -16.283   <2e-16 ***
## SpeciesW       0.017344   0.015653   1.108    0.270    
## SpeciesF2 RxW  0.005019   0.013918   0.361    0.719    
## SpeciesF2 WxR  0.013929   0.014114   0.987    0.326    
## SexMales      -0.510551   0.014406 -35.441   <2e-16 ***
## cer_rest       1.072796   0.092936  11.543   <2e-16 ***
## ---
## Signif. codes:  0 '***' 0.001 '**' 0.01 '*' 0.05 '.' 0.1 ' ' 1
## 
## Residual standard error: 0.05493 on 119 degrees of freedom
## Multiple R-squared:  0.9721, Adjusted R-squared:  0.9709 
## F-statistic:   828 on 5 and 119 DF,  p-value: < 2.2e-16
```

```
## Dorsal medulla
dm.m <- lm(dm ~ Species + Sex + dm_rest
           # + Species*Sex 
           # + Species*dm_rest 
           # + Sex*dm_rest 
           , data = brainData)
Anova(dm.m)
```

```
## Anova Table (Type II tests)
## 
## Response: dm
##            Sum Sq  Df F value    Pr(>F)    
## Species   0.00106   3  0.1025  0.958453    
## Sex       0.03019   1  8.7348  0.003765 ** 
## dm_rest   0.33616   1 97.2583 < 2.2e-16 ***
## Residuals 0.41131 119                      
## ---
## Signif. codes:  0 '***' 0.001 '**' 0.01 '*' 0.05 '.' 0.1 ' ' 1
```

```
summary(dm.m)
```

```
## 
## Call:
## lm(formula = dm ~ Species + Sex + dm_rest, data = brainData)
## 
## Residuals:
##       Min        1Q    Median        3Q       Max 
## -0.151034 -0.038901 -0.006934  0.041157  0.170203 
## 
## Coefficients:
##                Estimate Std. Error t value Pr(>|t|)    
## (Intercept)   -0.860419   0.046782 -18.392  < 2e-16 ***
## SpeciesW       0.004217   0.016758   0.252  0.80178    
## SpeciesF2 RxW -0.001322   0.014889  -0.089  0.92940    
## SpeciesF2 WxR  0.005385   0.015110   0.356  0.72218    
## SexMales       0.061145   0.020689   2.955  0.00376 ** 
## dm_rest        0.962511   0.097598   9.862  < 2e-16 ***
## ---
## Signif. codes:  0 '***' 0.001 '**' 0.01 '*' 0.05 '.' 0.1 ' ' 1
## 
## Residual standard error: 0.05879 on 119 degrees of freedom
## Multiple R-squared:  0.6393, Adjusted R-squared:  0.6241 
## F-statistic: 42.18 on 5 and 119 DF,  p-value: < 2.2e-16
```

```
## Olfactory bulbs
ob.m <- lm(ob ~ Species + Sex + ob_rest
           # + Species*Sex
           # + Species*ob_rest
           # + Sex*ob_rest
           , data = brainData)
Anova(ob.m)
```

```
## Anova Table (Type II tests)
## 
## Response: ob
##            Sum Sq  Df  F value    Pr(>F)    
## Species   0.04504   3   1.2612    0.2909    
## Sex       1.44726   1 121.5832 < 2.2e-16 ***
## ob_rest   0.21332   1  17.9207 4.557e-05 ***
## Residuals 1.41651 119                       
## ---
## Signif. codes:  0 '***' 0.001 '**' 0.01 '*' 0.05 '.' 0.1 ' ' 1
```

```
summary(ob.m)
```

```
## 
## Call:
## lm(formula = ob ~ Species + Sex + ob_rest, data = brainData)
## 
## Residuals:
##       Min        1Q    Median        3Q       Max 
## -0.309984 -0.068954  0.002669  0.064745  0.263276 
## 
## Coefficients:
##               Estimate Std. Error t value Pr(>|t|)    
## (Intercept)   -2.78288    0.09589 -29.020  < 2e-16 ***
## SpeciesW       0.04184    0.03110   1.345   0.1811    
## SpeciesF2 RxW  0.01839    0.02762   0.666   0.5067    
## SpeciesF2 WxR  0.04915    0.02804   1.753   0.0822 .  
## SexMales       0.40951    0.03714  11.026  < 2e-16 ***
## ob_rest        0.76337    0.18032   4.233 4.56e-05 ***
## ---
## Signif. codes:  0 '***' 0.001 '**' 0.01 '*' 0.05 '.' 0.1 ' ' 1
## 
## Residual standard error: 0.1091 on 119 degrees of freedom
## Multiple R-squared:  0.6452, Adjusted R-squared:  0.6303 
## F-statistic: 43.28 on 5 and 119 DF,  p-value: < 2.2e-16
```

```
## Hypothalamus
hyp.m <- lm(hyp ~ Species + Sex + hyp_rest
            # + Species*Sex
            # + Species*hyp_rest
            # + Sex*hyp_rest
            , data = brainData)
Anova(hyp.m)
```

```
## Anova Table (Type II tests)
## 
## Response: hyp
##            Sum Sq  Df F value    Pr(>F)    
## Species   0.04773   3  4.8946   0.00304 ** 
## Sex       0.01311   1  4.0325   0.04690 *  
## hyp_rest  0.28302   1 87.0635 7.203e-16 ***
## Residuals 0.38683 119                      
## ---
## Signif. codes:  0 '***' 0.001 '**' 0.01 '*' 0.05 '.' 0.1 ' ' 1
```

```
summary(hyp.m)
```

```
## 
## Call:
## lm(formula = hyp ~ Species + Sex + hyp_rest, data = brainData)
## 
## Residuals:
##       Min        1Q    Median        3Q       Max 
## -0.186225 -0.031530  0.001259  0.035142  0.151558 
## 
## Coefficients:
##               Estimate Std. Error t value Pr(>|t|)    
## (Intercept)   -0.87535    0.04548 -19.247  < 2e-16 ***
## SpeciesW      -0.01707    0.01627  -1.049   0.2963    
## SpeciesF2 RxW  0.03211    0.01441   2.229   0.0277 *  
## SpeciesF2 WxR  0.03040    0.01465   2.075   0.0401 *  
## SexMales       0.03977    0.01980   2.008   0.0469 *  
## hyp_rest       0.87246    0.09350   9.331  7.2e-16 ***
## ---
## Signif. codes:  0 '***' 0.001 '**' 0.01 '*' 0.05 '.' 0.1 ' ' 1
## 
## Residual standard error: 0.05701 on 119 degrees of freedom
## Multiple R-squared:  0.6724, Adjusted R-squared:  0.6587 
## F-statistic: 48.85 on 5 and 119 DF,  p-value: < 2.2e-16
```

```
summary( glht(hyp.m, mcp(Species = "Tukey")) )   # Post-hoc comparison of species groups
```

```
## 
##   Simultaneous Tests for General Linear Hypotheses
## 
## Multiple Comparisons of Means: Tukey Contrasts
## 
## 
## Fit: lm(formula = hyp ~ Species + Sex + hyp_rest, data = brainData)
## 
## Linear Hypotheses:
##                       Estimate Std. Error t value Pr(>|t|)   
## W - R == 0           -0.017067   0.016270  -1.049  0.71986   
## F2 RxW - R == 0       0.032110   0.014407   2.229  0.12075   
## F2 WxR - R == 0       0.030398   0.014647   2.075  0.16610   
## F2 RxW - W == 0       0.049177   0.015160   3.244  0.00821 **
## F2 WxR - W == 0       0.047465   0.015483   3.066  0.01408 * 
## F2 WxR - F2 RxW == 0 -0.001712   0.013205  -0.130  0.99921   
## ---
## Signif. codes:  0 '***' 0.001 '**' 0.01 '*' 0.05 '.' 0.1 ' ' 1
## (Adjusted p values reported -- single-step method)
```

---

#### Plot relative brain regions

```
knitr::include_graphics("F2-Females_legend.jpg")
```

```
## Females
## tel
ggplot(brainData  %>% 
         filter(Sex == "Females"), 
       aes(x = BrainVol_minusTel, y = TelVolTot, col = Species, shape = Species, linetype = Species)) +
  geom_point(size = 2, stroke = 1.8) +
  geom_smooth(method = "lm", se = TRUE, aes(fill = Species), alpha = 0.2) +
  ylab(expression(Volume ~(mm^3))) +
  xlab(expression('Rest of the brain' ~(mm^3))) +
  ggtitle("TEL volume") +
  scale_y_log10() + 
  scale_x_log10() +
  scale_linetype_manual(values = c("solid", "solid", "longdash", "dashed")) +
  scale_shape_manual(values = c(1, 2, 16, 17)) +
  scale_colour_manual(values=treatmentCol[c(1,2,5,6)])+
  scale_fill_manual(values=treatmentCol[c(1,2,5,6)])+
  theme_classic(base_size = 18) + theme(
    panel.background = element_blank(),
    panel.border = element_blank(),
    legend.position="none",
    axis.text = element_text(colour = "black"),
    axis.title.x = element_text(margin = unit(c(3, 0, 0, 0), "mm")),
    axis.title.y = element_text(margin = unit(c(0, 3, 0, 0), "mm")) )
## ot
ggplot(brainData  %>% 
         filter(Sex == "Females"), 
       aes(x = BrainVol_minusOT, y = OTVolTot, col = Species, shape = Species, linetype = Species)) +
  geom_point(size = 2, stroke = 1.8) +
  geom_smooth(method = "lm", se = TRUE, aes(fill = Species), alpha = 0.2) +
  ylab(expression(Volume ~(mm^3))) +
  xlab(expression('Rest of the brain' ~(mm^3))) +
  ggtitle("OT volume") +
  scale_y_log10() + 
  scale_x_log10() +
  scale_linetype_manual(values = c("solid", "solid", "longdash", "dashed")) +
  scale_shape_manual(values = c(1, 2, 16, 17)) +
  scale_colour_manual(values=treatmentCol[c(1,2,5,6)])+
  scale_fill_manual(values=treatmentCol[c(1,2,5,6)])+
  theme_classic(base_size = 18) + theme(
    panel.background = element_blank(),
    panel.border = element_blank(),
    legend.position="none",
    axis.text = element_text(colour = "black"),
    axis.title.x = element_text(margin = unit(c(3, 0, 0, 0), "mm")),
    axis.title.y = element_text(margin = unit(c(0, 3, 0, 0), "mm")) )
## cer
ggplot(brainData  %>% 
         filter(Sex == "Females"), 
       aes(x = BrainVol_minusCer, y = CerVolAvg, col = Species, shape = Species, linetype = Species)) +
  geom_point(size = 2, stroke = 1.8) +
  geom_smooth(method = "lm", se = TRUE, aes(fill = Species), alpha = 0.2) +
  ylab(expression(Volume ~(mm^3))) +
  xlab(expression('Rest of the brain' ~(mm^3))) +
  ggtitle("CER volume") +
  scale_y_log10() + 
  scale_x_log10() +
  scale_linetype_manual(values = c("solid", "solid", "longdash", "dashed")) +
  scale_shape_manual(values = c(1, 2, 16, 17)) +
  scale_colour_manual(values=treatmentCol[c(1,2,5,6)])+
  scale_fill_manual(values=treatmentCol[c(1,2,5,6)])+
  theme_classic(base_size = 18) + theme(
    panel.background = element_blank(),
    panel.border = element_blank(),
    legend.position="none",
    axis.text = element_text(colour = "black"),
    axis.title.x = element_text(margin = unit(c(3, 0, 0, 0), "mm")),
    axis.title.y = element_text(margin = unit(c(0, 3, 0, 0), "mm")) )
## Hyp
ggplot(brainData  %>% 
         filter(Sex == "Females"), 
       aes(x = BrainVol_minusHyp, y = HypVolTot, col = Species, shape = Species, linetype = Species)) +
  geom_point(size = 2, stroke = 1.8) +
  geom_smooth(method = "lm", se = TRUE, aes(fill = Species), alpha = 0.2) +
  ylab(expression(Volume ~(mm^3))) +
  xlab(expression('Rest of the brain' ~(mm^3))) +
  ggtitle("HYP volume") +
  scale_y_log10() + 
  scale_x_log10() +
  scale_linetype_manual(values = c("solid", "solid", "longdash", "dashed")) +
  scale_shape_manual(values = c(1, 2, 16, 17)) +
  scale_colour_manual(values=treatmentCol[c(1,2,5,6)])+
  scale_fill_manual(values=treatmentCol[c(1,2,5,6)])+
  theme_classic(base_size = 18) + theme(
    panel.background = element_blank(),
    panel.border = element_blank(),
    legend.position="none",
    axis.text = element_text(colour = "black"),
    axis.title.x = element_text(margin = unit(c(3, 0, 0, 0), "mm")),
    axis.title.y = element_text(margin = unit(c(0, 3, 0, 0), "mm")) )
## DM
ggplot(brainData  %>% 
         filter(Sex == "Females"), 
       aes(x = BrainVol_minusDM, y = DMVolAvg, col = Species, shape = Species, linetype = Species)) +
  geom_point(size = 2, stroke = 1.8) +
  geom_smooth(method = "lm", se = TRUE, aes(fill = Species), alpha = 0.2) +
  ylab(expression(Volume ~(mm^3))) +
  xlab(expression('Rest of the brain' ~(mm^3))) +
  ggtitle("DM volume") +
  scale_y_log10() + 
  scale_x_log10() +
  scale_linetype_manual(values = c("solid", "solid", "longdash", "dashed")) +
  scale_shape_manual(values = c(1, 2, 16, 17)) +
  scale_colour_manual(values=treatmentCol[c(1,2,5,6)])+
  scale_fill_manual(values=treatmentCol[c(1,2,5,6)])+
  theme_classic(base_size = 18) + theme(
    panel.background = element_blank(),
    panel.border = element_blank(),
    legend.position="none",
    axis.text = element_text(colour = "black"),
    axis.title.x = element_text(margin = unit(c(3, 0, 0, 0), "mm")),
    axis.title.y = element_text(margin = unit(c(0, 3, 0, 0), "mm")) )
## ob
ggplot(brainData  %>% 
         filter(Sex == "Females"), 
       aes(x = BrainVol_minusOB, y = OBVolTot, col = Species, shape = Species, linetype = Species)) +
  geom_point(size = 2, stroke = 1.8) +
  geom_smooth(method = "lm", se = TRUE, aes(fill = Species), alpha = 0.2) +
  ylab(expression(Volume ~(mm^3))) +
  xlab(expression('Rest of the brain' ~(mm^3))) +
  ggtitle("OB volume") +
  scale_y_log10() + 
  scale_x_log10() +
  scale_linetype_manual(values = c("solid", "solid", "longdash", "dashed")) +
  scale_shape_manual(values = c(1, 2, 16, 17)) +
  scale_colour_manual(values=treatmentCol[c(1,2,5,6)])+
  scale_fill_manual(values=treatmentCol[c(1,2,5,6)])+
  theme_classic(base_size = 18) + theme(
    panel.background = element_blank(),
    panel.border = element_blank(),
    legend.position="none",
    axis.text = element_text(colour = "black"),
    axis.title.x = element_text(margin = unit(c(3, 0, 0, 0), "mm")),
    axis.title.y = element_text(margin = unit(c(0, 3, 0, 0), "mm")) )
```

**Figure 3.2.2.1a.** Differences in relative brain region volume between parentals and F2 hybrids for females. Model predictions are plotted as the best fit line. Body length (x axis) and brain mass (y axis) are shown in log10 scales.

```
knitr::include_graphics("F2-Males_legend.jpg")
```

```
## Males
## tel
ggplot(brainData  %>% 
         filter(Sex == "Males"), 
       aes(x = BrainVol_minusTel, y = TelVolTot, col = Species, shape = Species, linetype = Species)) +
  geom_point(size = 2, stroke = 1.8) +
  geom_smooth(method = "lm", se = TRUE, aes(fill = Species), alpha = 0.2) +
  ylab(expression(Volume ~(mm^3))) +
  xlab(expression('Rest of the brain' ~(mm^3))) +
  ggtitle("TEL volume") +
  scale_y_log10() + 
  scale_x_log10() +
  scale_linetype_manual(values = c("solid", "solid", "longdash", "dashed")) +
  scale_shape_manual(values = c(1, 2, 16, 17)) +
  scale_colour_manual(values=treatmentCol[c(1,2,5,6)])+
  scale_fill_manual(values=treatmentCol[c(1,2,5,6)])+
  theme_classic(base_size = 18) + theme(
    panel.background = element_blank(),
    panel.border = element_blank(),
    legend.position="none",
    axis.text = element_text(colour = "black"),
    axis.title.x = element_text(margin = unit(c(3, 0, 0, 0), "mm")),
    axis.title.y = element_text(margin = unit(c(0, 3, 0, 0), "mm")) )
## ot
ggplot(brainData  %>% 
         filter(Sex == "Males"), 
       aes(x = BrainVol_minusOT, y = OTVolTot, col = Species, shape = Species, linetype = Species)) +
  geom_point(size = 2, stroke = 1.8) +
  geom_smooth(method = "lm", se = TRUE, aes(fill = Species), alpha = 0.2) +
  ylab(expression(Volume ~(mm^3))) +
  xlab(expression('Rest of the brain' ~(mm^3))) +
  ggtitle("OT volume") +
  scale_y_log10() + 
  scale_x_log10() +
  scale_linetype_manual(values = c("solid", "solid", "longdash", "dashed")) +
  scale_shape_manual(values = c(1, 2, 16, 17)) +
  scale_colour_manual(values=treatmentCol[c(1,2,5,6)])+
  scale_fill_manual(values=treatmentCol[c(1,2,5,6)])+
  theme_classic(base_size = 18) + theme(
    panel.background = element_blank(),
    panel.border = element_blank(),
    legend.position="none",
    axis.text = element_text(colour = "black"),
    axis.title.x = element_text(margin = unit(c(3, 0, 0, 0), "mm")),
    axis.title.y = element_text(margin = unit(c(0, 3, 0, 0), "mm")) )
## cer
ggplot(brainData  %>% 
         filter(Sex == "Males"), 
       aes(x = BrainVol_minusCer, y = CerVolAvg, col = Species, shape = Species, linetype = Species)) +
  geom_point(size = 2, stroke = 1.8) +
  geom_smooth(method = "lm", se = TRUE, aes(fill = Species), alpha = 0.2) +
  ylab(expression(Volume ~(mm^3))) +
  xlab(expression('Rest of the brain' ~(mm^3))) +
  ggtitle("CER volume") +
  scale_y_log10() + 
  scale_x_log10() +
  scale_linetype_manual(values = c("solid", "solid", "longdash", "dashed")) +
  scale_shape_manual(values = c(1, 2, 16, 17)) +
  scale_colour_manual(values=treatmentCol[c(1,2,5,6)])+
  scale_fill_manual(values=treatmentCol[c(1,2,5,6)])+
  theme_classic(base_size = 18) + theme(
    panel.background = element_blank(),
    panel.border = element_blank(),
    legend.position="none",
    axis.text = element_text(colour = "black"),
    axis.title.x = element_text(margin = unit(c(3, 0, 0, 0), "mm")),
    axis.title.y = element_text(margin = unit(c(0, 3, 0, 0), "mm")) )
## Hyp
ggplot(brainData  %>% 
         filter(Sex == "Males"), 
       aes(x = BrainVol_minusHyp, y = HypVolTot, col = Species, shape = Species, linetype = Species)) +
  geom_point(size = 2, stroke = 1.8) +
  geom_smooth(method = "lm", se = TRUE, aes(fill = Species), alpha = 0.2) +
  ylab(expression(Volume ~(mm^3))) +
  xlab(expression('Rest of the brain' ~(mm^3))) +
  ggtitle("HYP volume") +
  scale_y_log10() + 
  scale_x_log10() +
  scale_linetype_manual(values = c("solid", "solid", "longdash", "dashed")) +
  scale_shape_manual(values = c(1, 2, 16, 17)) +
  scale_colour_manual(values=treatmentCol[c(1,2,5,6)])+
  scale_fill_manual(values=treatmentCol[c(1,2,5,6)])+
  theme_classic(base_size = 18) + theme(
    panel.background = element_blank(),
    panel.border = element_blank(),
    legend.position="none",
    axis.text = element_text(colour = "black"),
    axis.title.x = element_text(margin = unit(c(3, 0, 0, 0), "mm")),
    axis.title.y = element_text(margin = unit(c(0, 3, 0, 0), "mm")) )
## DM
ggplot(brainData  %>% 
         filter(Sex == "Males"), 
       aes(x = BrainVol_minusDM, y = DMVolAvg, col = Species, shape = Species, linetype = Species)) +
  geom_point(size = 2, stroke = 1.8) +
  geom_smooth(method = "lm", se = TRUE, aes(fill = Species), alpha = 0.2) +
  ylab(expression(Volume ~(mm^3))) +
  xlab(expression('Rest of the brain' ~(mm^3))) +
  ggtitle("DM volume") +
  scale_y_log10() + 
  scale_x_log10() +
  scale_linetype_manual(values = c("solid", "solid", "longdash", "dashed")) +
  scale_shape_manual(values = c(1, 2, 16, 17)) +
  scale_colour_manual(values=treatmentCol[c(1,2,5,6)])+
  scale_fill_manual(values=treatmentCol[c(1,2,5,6)])+
  theme_classic(base_size = 18) + theme(
    panel.background = element_blank(),
    panel.border = element_blank(),
    legend.position="none",
    axis.text = element_text(colour = "black"),
    axis.title.x = element_text(margin = unit(c(3, 0, 0, 0), "mm")),
    axis.title.y = element_text(margin = unit(c(0, 3, 0, 0), "mm")) )
## ob
ggplot(brainData  %>% 
         filter(Sex == "Males"), 
       aes(x = BrainVol_minusOB, y = OBVolTot, col = Species, shape = Species, linetype = Species)) +
  geom_point(size = 2, stroke = 1.8) +
  geom_smooth(method = "lm", se = TRUE, aes(fill = Species), alpha = 0.2) +
  ylab(expression(Volume ~(mm^3))) +
  xlab(expression('Rest of the brain' ~(mm^3))) +
  ggtitle("OB volume") +
  scale_y_log10() + 
  scale_x_log10() +
  scale_linetype_manual(values = c("solid", "solid", "longdash", "dashed")) +
  scale_shape_manual(values = c(1, 2, 16, 17)) +
  scale_colour_manual(values=treatmentCol[c(1,2,5,6)])+
  scale_fill_manual(values=treatmentCol[c(1,2,5,6)])+
  theme_classic(base_size = 18) + theme(
    panel.background = element_blank(),
    panel.border = element_blank(),
    legend.position="none",
    axis.text = element_text(colour = "black"),
    axis.title.x = element_text(margin = unit(c(3, 0, 0, 0), "mm")),
    axis.title.y = element_text(margin = unit(c(0, 3, 0, 0), "mm")) )
```

**Figure 3.2.2.1b.** Differences in relative brain region volume between parentals and F2 hybrids for males. Model predictions are plotted as the best fit line. Body length (x axis) and brain mass (y axis) are shown in log10 scales.

# 4 Learning performance analyses

## 4.1 F1 and parental females

Detailed methods and analyses for F1 female fish can be found in Vila Pouca et al. 2022 Am Nat.

---

## 4.2 F2 and parental females

### 4.2.1 Associative learning

```
# Count of fish that learnt per species
LCFem2 %>% 
  group_by(Species) %>%
  summarise( nFail = length(Success_AL) - sum(Success_AL),
             nSuccess = sum(Success_AL),
             nTotal = length(Success_AL),
             propGroup = nSuccess/nTotal)
```

```
## # A tibble: 4 x 5
##   Species nFail nSuccess nTotal propGroup
## * <fct>   <int>    <int>  <int>     <dbl>
## 1 R           2       11     13     0.846
## 2 W           0       10     10     1    
## 3 RW_F2       3       22     25     0.88 
## 4 WR_F2       5       19     24     0.792
```

```
## ~~~~~~
## TRIALS TO LEARNING CRITERION

# Subset to learners only
learnersAL_Fem2 <- subset(LCFem2, Success_AL == 1)
# Response var: Trials to LC (poisson, count data)
# Potential predictors: Species + Colour
LC.f2 <- glm(data = learnersAL_Fem2, TrialsLC_AL ~ Species + ColourAL, family = "poisson")
Anova(LC.f2)
```

```
## Analysis of Deviance Table (Type II tests)
## 
## Response: TrialsLC_AL
##          LR Chisq Df Pr(>Chisq)    
## Species    11.729  3   0.008373 ** 
## ColourAL   57.316  1  3.711e-14 ***
## ---
## Signif. codes:  0 '***' 0.001 '**' 0.01 '*' 0.05 '.' 0.1 ' ' 1
```

```
summary(LC.f2)
```

```
## 
## Call:
## glm(formula = TrialsLC_AL ~ Species + ColourAL, family = "poisson", 
##     data = learnersAL_Fem2)
## 
## Deviance Residuals: 
##     Min       1Q   Median       3Q      Max  
## -4.7038  -1.4946  -0.1955   1.0568   4.0826  
## 
## Coefficients:
##                Estimate Std. Error z value Pr(>|z|)    
## (Intercept)     2.63608    0.08017  32.882  < 2e-16 ***
## SpeciesW        0.04697    0.10202   0.460   0.6452    
## SpeciesRW_F2    0.25137    0.08380   2.999   0.0027 ** 
## SpeciesWR_F2    0.13982    0.08866   1.577   0.1148    
## ColourALYellow  0.43358    0.05767   7.519 5.52e-14 ***
## ---
## Signif. codes:  0 '***' 0.001 '**' 0.01 '*' 0.05 '.' 0.1 ' ' 1
## 
## (Dispersion parameter for poisson family taken to be 1)
## 
##     Null deviance: 281.16  on 61  degrees of freedom
## Residual deviance: 212.03  on 57  degrees of freedom
## AIC: 515.3
## 
## Number of Fisher Scoring iterations: 4
```

```
## Post-hoc comparison of groups
summary( glht(LC.f2, mcp(Species = "Tukey")) )   # from package multcomp
```

```
## 
##   Simultaneous Tests for General Linear Hypotheses
## 
## Multiple Comparisons of Means: Tukey Contrasts
## 
## 
## Fit: glm(formula = TrialsLC_AL ~ Species + ColourAL, family = "poisson", 
##     data = learnersAL_Fem2)
## 
## Linear Hypotheses:
##                    Estimate Std. Error z value Pr(>|z|)  
## W - R == 0          0.04697    0.10202   0.460    0.967  
## RW_F2 - R == 0      0.25137    0.08380   2.999    0.014 *
## WR_F2 - R == 0      0.13982    0.08866   1.577    0.387  
## RW_F2 - W == 0      0.20440    0.08584   2.381    0.079 .
## WR_F2 - W == 0      0.09284    0.09037   1.027    0.730  
## WR_F2 - RW_F2 == 0 -0.11155    0.06916  -1.613    0.366  
## ---
## Signif. codes:  0 '***' 0.001 '**' 0.01 '*' 0.05 '.' 0.1 ' ' 1
## (Adjusted p values reported -- single-step method)
```

---

#### Plot trials to learning criterion

```
ggplot(LCFem, aes(x = factor(Species), y = TrialsLC_AL,
               fill = Species, colour = Species)) +
  geom_violin(trim = FALSE, alpha = 0.4, width = 0.9, colour = NA) +
  geom_boxplot(width=0.07, aes(colour = Species), fill = "grey90", outlier.shape = NA) +
  geom_point(position = position_jitter(w = 0.1, h = 0.05), pch = 19, colour = "grey50", size = 1.5) +
  ylab("Trials to learning criterion") +
  xlab("Species group") +
  scale_fill_manual(values = treatmentCol) +
  scale_color_manual(values = treatmentCol) +
  scale_y_continuous(breaks = seq(0,70,10), limits = c(0,70), expand = expansion(mult = c(0, 0))) +
  theme_classic(base_size = 11) + theme(
    panel.background = element_blank(),
    panel.border = element_blank(),
    legend.position="none",
    axis.text = element_text(colour = "black"),
    axis.title.x = element_text(margin = unit(c(3, 0, 0, 0), "mm")),
    axis.title.y = element_text(margin = unit(c(0, 3, 0, 0), "mm")) )
```

**Figure 4.2.1.1.** Trials taken to reach learning criterion in the associative learning task, for females of each species group.

```
## ~~~~~~
## SUCCESS RATE OVER TRIALS

## Some fish had too many null trials, i.e., did not engage in the task during colour training
## Identify individuals that failed to choose in >60% of the trials and remove from learning curve analyses 
keep <- c("TankID", "Species", "ColourAL")
nullAL <- assocLFem2 %>%
  group_by(TankID) %>%
  summarise( freqNA = sum(!complete.cases(Choice)),
             nTotal = length(Choice),
             propNA = freqNA/nTotal)
nullAL <- merge(nullAL, subset(infoFem2, select = keep), by = "TankID")
## Get ID of fish that had > 60% trials without choice
nullIndiv <- as.character( nullAL[nullAL$propNA >= 0.6, "TankID"] )
print(paste0("Individuals to exclude: ", nullIndiv))
```

```
## [1] "Individuals to exclude: T23"
```

```
## Exclude individual from dataset
assocLFem2 <- subset(assocLFem2, !(TankID  %in% nullIndiv))
assocLFem2$TankID <- factor(assocLFem2$TankID)
assocLFem2$ColourAL <- factor(assocLFem2$ColourAL)

## >>> Analyses on the 71 remaining females

# make NA as wrong choice for learning rate analyses
assocLFem2[is.na(assocLFem2$Choice), "Choice"] <- 0

# Sample sizes
overview <- subset(assocLFem2, Trial == 1)
overview$SpCol <- paste0(overview$Species, overview$ColourAL)
table(overview$Species) # by species group
```

```
## 
##     R     W RW_F2 WR_F2 
##    12    10    25    24
```

```
table(overview$SpCol) # by species group & colour
```

```
## 
##        RRed    RW_F2Red RW_F2Yellow     RYellow    WR_F2Red WR_F2Yellow 
##           6          12          13           6          12          12 
##        WRed     WYellow 
##           5           5
```

```
## ~~~~~~
# Success rate AL
# Response var: Choice (binomial, 1 = correct; 0 = incorrect)
# Potential predictors: Species; Colour; Trial; Species*Trial; Colour*Trial
# Random intercept and slope: fish ID
assocL.m1 <- glmer(Choice ~ Species + ColourAL + Trial +
                     # Side + 
                     Species*Trial +
                     ColourAL*Trial +
                     # ColourAL*Species +
                     (Trial|TankID),
                   family = binomial, control = glmerControl("bobyqa"), data = assocLFem2)
# Model output
Anova(assocL.m1)
```

```
## Analysis of Deviance Table (Type II Wald chisquare tests)
## 
## Response: Choice
##                  Chisq Df Pr(>Chisq)    
## Species         1.9688  3    0.57890    
## ColourAL       17.8064  1  2.446e-05 ***
## Trial          70.5753  1  < 2.2e-16 ***
## Species:Trial   2.9763  3    0.39529    
## ColourAL:Trial  3.1594  1    0.07549 .  
## ---
## Signif. codes:  0 '***' 0.001 '**' 0.01 '*' 0.05 '.' 0.1 ' ' 1
```

```
summary(assocL.m1)
```

```
## Generalized linear mixed model fit by maximum likelihood (Laplace
##   Approximation) [glmerMod]
##  Family: binomial  ( logit )
## Formula: Choice ~ Species + ColourAL + Trial + Species * Trial + ColourAL *  
##     Trial + (Trial | TankID)
##    Data: assocLFem2
## Control: glmerControl("bobyqa")
## 
##      AIC      BIC   logLik deviance df.resid 
##   1965.0   2035.9   -969.5   1939.0     1711 
## 
## Scaled residuals: 
##     Min      1Q  Median      3Q     Max 
## -4.3612 -0.7371  0.3402  0.6206  2.9562 
## 
## Random effects:
##  Groups Name        Variance Std.Dev. Corr 
##  TankID (Intercept) 0.951457 0.97543       
##         Trial       0.004846 0.06961  -0.25
## Number of obs: 1724, groups:  TankID, 71
## 
## Fixed effects:
##                      Estimate Std. Error z value Pr(>|z|)    
## (Intercept)          -0.04657    0.45375  -0.103   0.9183    
## SpeciesW             -0.54039    0.60569  -0.892   0.3723    
## SpeciesRW_F2         -0.27198    0.48328  -0.563   0.5736    
## SpeciesWR_F2         -0.32886    0.48857  -0.673   0.5009    
## ColourALYellow       -0.84153    0.33095  -2.543   0.0110 *  
## Trial                 0.20005    0.04456   4.490 7.13e-06 ***
## SpeciesW:Trial        0.03268    0.05292   0.618   0.5369    
## SpeciesRW_F2:Trial   -0.03819    0.04060  -0.941   0.3469    
## SpeciesWR_F2:Trial   -0.03120    0.04082  -0.765   0.4446    
## ColourALYellow:Trial -0.05332    0.03000  -1.777   0.0755 .  
## ---
## Signif. codes:  0 '***' 0.001 '**' 0.01 '*' 0.05 '.' 0.1 ' ' 1
## 
## Correlation of Fixed Effects:
##             (Intr) SpecsW SpRW_F2 SpWR_F2 ClrALY Trial  SpcW:T SRW_F2: SWR_F2:
## SpeciesW    -0.594                                                            
## SpecisRW_F2 -0.758  0.555                                                     
## SpecisWR_F2 -0.728  0.549  0.688                                              
## ColorALYllw -0.424  0.005  0.015  -0.012                                      
## Trial       -0.578  0.275  0.386   0.336   0.274                              
## SpecisW:Trl  0.304 -0.563 -0.294  -0.292   0.019 -0.485                       
## SpcsRW_F2:T  0.453 -0.306 -0.536  -0.380  -0.036 -0.758  0.550                
## SpcsWR_F2:T  0.402 -0.305 -0.386  -0.534   0.030 -0.657  0.551  0.726         
## ClrALYllw:T  0.311  0.018 -0.033   0.032  -0.551 -0.563 -0.044  0.104  -0.034
```

```
# Test significance of random effects
# m2: random intercept only
# m3: random slope only
assocL.m2 <- glmer(Choice ~ Species + ColourAL + Trial +
                     Species*Trial +
                     ColourAL*Trial +
                     (1|TankID),
                   family = binomial, control = glmerControl("bobyqa"), data = assocLFem2)
assocL.m3 <- glmer(Choice ~ Species + ColourAL + Trial +
                     Species*Trial +
                     ColourAL*Trial +
                     (0+Trial|TankID),
                   family = binomial, control = glmerControl("bobyqa"), data = assocLFem2)

anova(assocL.m1, assocL.m2) # test for inclusion of slope
```

```
## Data: assocLFem2
## Models:
## assocL.m2: Choice ~ Species + ColourAL + Trial + Species * Trial + ColourAL * 
## assocL.m2:     Trial + (1 | TankID)
## assocL.m1: Choice ~ Species + ColourAL + Trial + Species * Trial + ColourAL * 
## assocL.m1:     Trial + (Trial | TankID)
##           Df  AIC    BIC  logLik deviance  Chisq Chi Df Pr(>Chisq)    
## assocL.m2 11 2000 2060.0 -989.00     1978                             
## assocL.m1 13 1965 2035.8 -969.49     1939 39.035      2  3.339e-09 ***
## ---
## Signif. codes:  0 '***' 0.001 '**' 0.01 '*' 0.05 '.' 0.1 ' ' 1
```

```
anova(assocL.m1, assocL.m3) # test for inclusion of intercept
```

```
## Data: assocLFem2
## Models:
## assocL.m3: Choice ~ Species + ColourAL + Trial + Species * Trial + ColourAL * 
## assocL.m3:     Trial + (0 + Trial | TankID)
## assocL.m1: Choice ~ Species + ColourAL + Trial + Species * Trial + ColourAL * 
## assocL.m1:     Trial + (Trial | TankID)
##           Df    AIC    BIC  logLik deviance  Chisq Chi Df Pr(>Chisq)    
## assocL.m3 11 1990.2 2050.2 -984.11   1968.2                             
## assocL.m1 13 1965.0 2035.8 -969.49   1939.0 29.257      2  4.435e-07 ***
## ---
## Signif. codes:  0 '***' 0.001 '**' 0.01 '*' 0.05 '.' 0.1 ' ' 1
```

#### Plot group learning curves

```
maxTrialsAL <- max(assocLFem2$Trial) # 40
# model group predictions 
assocL.m1_fit <- effect('Species*Trial',assocL.m1, xlevels = maxTrialsAL, na.rm=T) %>% as.data.frame()
assocL.m1_fit$Species <- factor(assocL.m1_fit$Species, levels = c("R", "W", "RW_F2", "WR_F2"))

ggplot()  + 
  geom_ribbon(data = assocL.m1_fit, col = NA, alpha = 0.2, size = 4,
              aes(Trial, NULL, ymin = lower, ymax = upper, fill = Species)) +
  geom_line(data = assocL.m1_fit, aes(Trial, fit, colour = Species, linetype = Species), size = 0.8)  +
  scale_fill_manual(values = treatmentCol[c(1,2,5,6)]) +
  scale_color_manual(values = treatmentCol[c(1,2,5,6)]) +
  scale_linetype_manual(values = c("solid", "solid", "longdash", "dashed")) +
  scale_x_continuous(breaks = seq(0,maxTrialsAL,5), limits = c(0,maxTrialsAL), expand = expansion(mult = c(0, 0))) +
  scale_y_continuous(breaks = seq(0,1,0.2), limits = c(0,1), expand = expansion(mult = c(0, 0))) +
  labs(x = "Trials",y="Success") +
  theme_classic(base_size = 11) + theme(
    panel.background = element_blank(),
    panel.border = element_blank(),
    legend.position = c(0.9, 0.28),
    axis.text = element_text(colour = "black"),
    axis.title.x = element_text(margin = unit(c(3, 0, 0, 0), "mm")),
    axis.title.y = element_text(margin = unit(c(0, 3, 0, 0), "mm"))    )
```

**Figure 4.2.1.2.** Probability of correct choice over trials for females of each species group in the associative learning task. Lines show predicted model outputs and shaded areas indicate 95% confidence intervals.

#### Plot individual learning curves

```
ggplot()  + 
  binomial_smooth(data = assocLFem2, size = 0.4, colour = "grey80", aes(x = Trial, y = Choice, group = FishID)) +
  geom_ribbon(data = assocL.m1_fit, col = NA, alpha = 0.2, size = 4,
              aes(Trial, NULL, ymin = lower, ymax = upper, fill = Species)) +
  geom_line(data = assocL.m1_fit, aes(Trial, fit, colour = Species, linetype = Species), size = 0.8)  +
  facet_wrap(~ Species) + # Facet wrap by species
  scale_fill_manual(values = treatmentCol[c(1,2,5,6)]) +
  scale_color_manual(values = treatmentCol[c(1,2,5,6)]) +
  scale_linetype_manual(values = c("solid", "dashed", "dashed", "solid")) +
  scale_y_continuous(breaks = seq(0,1,0.2), limits = c(0,1), expand = expansion(mult = c(0, 0))) +
  labs(x = "Trials",y="Success") +
  ggtitle("Associative Learning") +
  theme_classic(base_size = 11) + theme(
    panel.background = element_blank(),
    panel.border = element_blank(),
    legend.position = "none",
    axis.text = element_text(colour = "black"),
    axis.title.x = element_text(margin = unit(c(3, 0, 0, 0), "mm")),
    axis.title.y = element_text(margin = unit(c(0, 3, 0, 0), "mm")),
    plot.title = element_text(hjust = 0))
```

**Figure 4.2.1.3.** Probability of correct choice over trials for females of each species group in the associative learning task. Coloured lines show predicted model outputs for each species group and shaded areas indicate 95% confidence intervals. Thin grey lines show individual curves.

### 4.2.2 Reversal learning

```
# Count of fish that learnt per species
LCFem2 %>% 
  drop_na(Success_RL) %>%
  group_by(Species) %>%
  summarise( nFail = length(Success_RL) - sum(Success_RL),
             nSuccess = sum(Success_RL, na.rm = TRUE),
             nTotal = n(),
             propGroup = nSuccess/nTotal)
```

```
## # A tibble: 4 x 5
##   Species nFail nSuccess nTotal propGroup
## * <fct>   <int>    <int>  <int>     <dbl>
## 1 R           0       11     11     1    
## 2 W           0       10     10     1    
## 3 RW_F2       0       22     22     1    
## 4 WR_F2       2       17     19     0.895
```

```
## ~~~~~~
## TRIALS TO LEARNING CRITERION

# Subset to learners only
learnersRL_Fem2 <- subset(LCFem2, Success_RL == 1)
# Response var: Trials to LC (poisson, count data)
# Potential predictors: Species + Colour
LC.f2.1 <- glm(data = learnersRL_Fem2, TrialsLC_RL ~ Species + ColourRL
             , family = "poisson")
Anova(LC.f2.1)
```

```
## Analysis of Deviance Table (Type II tests)
## 
## Response: TrialsLC_RL
##          LR Chisq Df Pr(>Chisq)    
## Species     8.125  3     0.0435 *  
## ColourRL   32.133  1   1.44e-08 ***
## ---
## Signif. codes:  0 '***' 0.001 '**' 0.01 '*' 0.05 '.' 0.1 ' ' 1
```

```
summary(LC.f2.1)
```

```
## 
## Call:
## glm(formula = TrialsLC_RL ~ Species + ColourRL, family = "poisson", 
##     data = learnersRL_Fem2)
## 
## Deviance Residuals: 
##    Min      1Q  Median      3Q     Max  
## -3.516  -1.822  -0.405   1.229   4.696  
## 
## Coefficients:
##                Estimate Std. Error z value Pr(>|z|)    
## (Intercept)     3.19360    0.06319  50.538  < 2e-16 ***
## SpeciesW       -0.19084    0.08642  -2.208  0.02722 *  
## SpeciesRW_F2   -0.18930    0.07179  -2.637  0.00837 ** 
## SpeciesWR_F2   -0.10159    0.07412  -1.371  0.17049    
## ColourRLYellow  0.29527    0.05245   5.630  1.8e-08 ***
## ---
## Signif. codes:  0 '***' 0.001 '**' 0.01 '*' 0.05 '.' 0.1 ' ' 1
## 
## (Dispersion parameter for poisson family taken to be 1)
## 
##     Null deviance: 267.32  on 59  degrees of freedom
## Residual deviance: 227.18  on 55  degrees of freedom
## AIC: 536.16
## 
## Number of Fisher Scoring iterations: 4
```

```
## Post-hoc comparison of groups
summary( glht(LC.f2.1, mcp(Species = "Tukey")) )   # from package multcomp
```

```
## 
##   Simultaneous Tests for General Linear Hypotheses
## 
## Multiple Comparisons of Means: Tukey Contrasts
## 
## 
## Fit: glm(formula = TrialsLC_RL ~ Species + ColourRL, family = "poisson", 
##     data = learnersRL_Fem2)
## 
## Linear Hypotheses:
##                    Estimate Std. Error z value Pr(>|z|)  
## W - R == 0         -0.19084    0.08642  -2.208   0.1193  
## RW_F2 - R == 0     -0.18931    0.07179  -2.637   0.0414 *
## WR_F2 - R == 0     -0.10159    0.07412  -1.371   0.5150  
## RW_F2 - W == 0      0.00154    0.07849   0.020   1.0000  
## WR_F2 - W == 0      0.08925    0.08049   1.109   0.6817  
## WR_F2 - RW_F2 == 0  0.08771    0.06453   1.359   0.5223  
## ---
## Signif. codes:  0 '***' 0.001 '**' 0.01 '*' 0.05 '.' 0.1 ' ' 1
## (Adjusted p values reported -- single-step method)
```

---

#### Plot trials to learning criterion

```
ggplot(LCFem, aes(x = factor(Species), y = TrialsLC_RL,
               fill = Species, colour = Species)) +
  geom_violin(trim = FALSE, alpha = 0.4, width = 0.9, colour = NA) +
  geom_boxplot(width=0.07, aes(colour = Species), fill = "grey90", outlier.shape = NA) +
  geom_point(position = position_jitter(w = 0.1, h = 0.05), pch = 19, colour = "grey50", size = 1.5) +
  ylab("Trials to learning criterion") +
  xlab("Species group") +
  scale_fill_manual(values = treatmentCol) +
  scale_color_manual(values = treatmentCol) +
  scale_y_continuous(breaks = seq(0,70,10), limits = c(0,70), expand = expansion(mult = c(0, 0))) +
  theme_classic(base_size = 11) + theme(
    panel.background = element_blank(),
    panel.border = element_blank(),
    legend.position="none",
    axis.text = element_text(colour = "black"),
    axis.title.x = element_text(margin = unit(c(3, 0, 0, 0), "mm")),
    axis.title.y = element_text(margin = unit(c(0, 3, 0, 0), "mm")) )
```

**Figure 4.2.2.1.** Trials taken to reach learning criterion in the reversal learning task, for females of each species group.

```
# Assessing the effect of previous task (TrialsLC_AL)
LC.f2.2 <- glm(data = learnersRL_Fem2, TrialsLC_RL ~ Species + ColourRL +
               Species*TrialsLC_AL
             , family = "poisson")
Anova(LC.f2.2)
```

```
## Analysis of Deviance Table (Type II tests)
## 
## Response: TrialsLC_RL
##                     LR Chisq Df Pr(>Chisq)    
## Species               6.8145  3    0.07805 .  
## ColourRL             17.1905  1  3.381e-05 ***
## TrialsLC_AL           0.7346  1    0.39138    
## Species:TrialsLC_AL   9.9300  3    0.01917 *  
## ---
## Signif. codes:  0 '***' 0.001 '**' 0.01 '*' 0.05 '.' 0.1 ' ' 1
```

```
summary(LC.f2.2)
```

```
## 
## Call:
## glm(formula = TrialsLC_RL ~ Species + ColourRL + Species * TrialsLC_AL, 
##     family = "poisson", data = learnersRL_Fem2)
## 
## Deviance Residuals: 
##     Min       1Q   Median       3Q      Max  
## -3.4345  -1.6399  -0.3177   1.2812   4.3819  
## 
## Coefficients:
##                           Estimate Std. Error z value Pr(>|z|)    
## (Intercept)               3.100247   0.139232  22.267  < 2e-16 ***
## SpeciesW                  0.309562   0.194638   1.590  0.11173    
## SpeciesRW_F2             -0.118610   0.171619  -0.691  0.48949    
## SpeciesWR_F2              0.159331   0.156014   1.021  0.30713    
## ColourRLYellow            0.257431   0.062524   4.117 3.83e-05 ***
## TrialsLC_AL               0.006457   0.006312   1.023  0.30634    
## SpeciesW:TrialsLC_AL     -0.028715   0.010249  -2.802  0.00508 ** 
## SpeciesRW_F2:TrialsLC_AL -0.004505   0.007871  -0.572  0.56704    
## SpeciesWR_F2:TrialsLC_AL -0.014638   0.007833  -1.869  0.06165 .  
## ---
## Signif. codes:  0 '***' 0.001 '**' 0.01 '*' 0.05 '.' 0.1 ' ' 1
## 
## (Dispersion parameter for poisson family taken to be 1)
## 
##     Null deviance: 267.32  on 59  degrees of freedom
## Residual deviance: 216.51  on 51  degrees of freedom
## AIC: 533.49
## 
## Number of Fisher Scoring iterations: 4
```

#### Plot task correlation

```
## Plot correlation trials to learn in AL & RL by species ----
LCFem2$Species <- factor(LCFem2$Species, levels = c("R", "W", "RW_F2", "WR_F2"))
ggplot(data = LCFem2, aes(x = TrialsLC_AL, y = TrialsLC_RL, colour = Species)) +
  geom_smooth(method = "lm", se = TRUE, alpha = 0.2, aes(fill = Species, linetype = Species)) +
  geom_point(aes(shape = Species), size = 2, stroke = 1.5, position = position_jitter(w = 0.15, h = 0.15)) +
  scale_shape_manual(values = c(1, 2, 16, 17)) +
  scale_color_manual(values = treatmentCol[c(1,2,5,6)]) +
  scale_fill_manual(values = treatmentCol[c(1,2,5,6)]) +
  scale_linetype_manual(values = c("solid", "solid", "longdash", "dashed")) +
  ylab("Trials to succeed reversal") +
  xlab("Trials to succeed association") +
  theme_classic(base_size = 11) + theme(
    panel.background = element_blank(),
    panel.border = element_blank(),
    axis.text = element_text(colour = "black"),
    axis.title.x = element_text(margin = unit(c(3, 0, 0, 0), "mm")),
    axis.title.y = element_text(margin = unit(c(0, 3, 0, 0), "mm")) )
```

**Figure 4.2.2.2.** Relation between individual performance in the associative learning and the reversal learning task, showing a negative correlation for Endler’s guppies (W) alone.

```
## ~~~~~~
## SUCCESS RATE OVER TRIALS

# make NA as wrong choice for learning rate analyses
reversLFem2[is.na(reversLFem2$Choice), "Choice"] <- 0

# Sample sizes
overview <- subset(reversLFem2, Trial == 1)
overview$SpCol <- paste0(overview$Species, overview$ColourRL)
table(overview$Species) # by species group
```

```
## 
##     R     W RW_F2 WR_F2 
##    11    10    22    19
```

```
table(overview$SpCol) # by species group & colour
```

```
## 
##        RRed    RW_F2Red RW_F2Yellow     RYellow    WR_F2Red WR_F2Yellow 
##           6          11          11           5           7          12 
##        WRed     WYellow 
##           5           5
```

```
## Add data from previous task
## Overall probability of success during Assoc. learning by individual
indivSuccess <- assocLFem2 %>%
  group_by(TankID) %>%
  summarise( successAL = mean(Choice) )
reversLFem2 <- merge(reversLFem2, indivSuccess, by = "TankID")
## Trials to LC in AL
reversLFem2 <- merge(reversLFem2, LCFem2[, c(3,6)], by = "TankID")

## ~~~~~~
# Success rate RL
# Response var: Choice (binomial, 1 = correct; 0 = incorrect)
# Potential predictors: Species; Colour; Trial; Species*Trial; Colour*Trial; Species*successAL; Species*TrialsLC_AL
# Random intercept and slope: fish ID
reversL.m1 <- glmer(Choice ~ Species + Trial + ColourRL + 
                      # Species*successAL +
                      Species*TrialsLC_AL +
                      Species*Trial +
                      # ColourRL*Trial +
                      (Trial|TankID),
                    family = binomial, control = glmerControl("bobyqa"), data = reversLFem2)
# Check model output to see if we can/should drop terms
Anova(reversL.m1)
```

```
## Analysis of Deviance Table (Type II Wald chisquare tests)
## 
## Response: Choice
##                        Chisq Df Pr(>Chisq)    
## Species               2.9890  3    0.39333    
## Trial               157.2492  1  < 2.2e-16 ***
## ColourRL             32.4997  1  1.192e-08 ***
## TrialsLC_AL           0.2274  1    0.63344    
## Species:TrialsLC_AL   6.6281  3    0.08475 .  
## Species:Trial         1.2341  3    0.74483    
## ---
## Signif. codes:  0 '***' 0.001 '**' 0.01 '*' 0.05 '.' 0.1 ' ' 1
```

```
# Test if log(Trial) improves fit:
reversLFem2$logTrial <- log(reversLFem2$Trial)
reversL.m1.1 <- glmer(Choice ~ Species + logTrial + ColourRL +
                        Species*TrialsLC_AL +
                        Species*logTrial +
                        (logTrial|TankID), 
                      family = binomial, control = glmerControl("bobyqa"), data = reversLFem2)
AIC(reversL.m1, reversL.m1.1) # log(Trial) improves model fit
```

```
##              df      AIC
## reversL.m1   16 2126.816
## reversL.m1.1 16 2101.520
```

```
# Check model output to see if we can/should drop terms
Anova(reversL.m1.1)
```

```
## Analysis of Deviance Table (Type II Wald chisquare tests)
## 
## Response: Choice
##                        Chisq Df Pr(>Chisq)    
## Species               2.8223  3    0.41984    
## logTrial            194.0915  1  < 2.2e-16 ***
## ColourRL             32.7570  1  1.044e-08 ***
## TrialsLC_AL           0.5599  1    0.45429    
## Species:TrialsLC_AL   6.3768  3    0.09465 .  
## Species:logTrial      0.3294  3    0.95440    
## ---
## Signif. codes:  0 '***' 0.001 '**' 0.01 '*' 0.05 '.' 0.1 ' ' 1
```

```
# Final model
reversL.m1.1 <- glmer(Choice ~ Species + logTrial + ColourRL +
                        # Species*TrialsLC_AL +
                        Species*logTrial +
                        (logTrial|TankID), 
                      family = binomial, control = glmerControl("bobyqa"), data = reversLFem2)
## ~~~~~~
# Model output
Anova(reversL.m1.1)
```

```
## Analysis of Deviance Table (Type II Wald chisquare tests)
## 
## Response: Choice
##                     Chisq Df Pr(>Chisq)    
## Species            2.4234  3     0.4893    
## logTrial         195.2705  1  < 2.2e-16 ***
## ColourRL          34.0997  1  5.236e-09 ***
## Species:logTrial   0.1301  3     0.9880    
## ---
## Signif. codes:  0 '***' 0.001 '**' 0.01 '*' 0.05 '.' 0.1 ' ' 1
```

```
summary(reversL.m1.1)
```

```
## Generalized linear mixed model fit by maximum likelihood (Laplace
##   Approximation) [glmerMod]
##  Family: binomial  ( logit )
## Formula: Choice ~ Species + logTrial + ColourRL + Species * logTrial +  
##     (logTrial | TankID)
##    Data: reversLFem2
## Control: glmerControl("bobyqa")
## 
##      AIC      BIC   logLik deviance df.resid 
##   2100.2   2166.9  -1038.1   2076.2     1896 
## 
## Scaled residuals: 
##     Min      1Q  Median      3Q     Max 
## -4.1422 -0.6911  0.3908  0.6136  4.5954 
## 
## Random effects:
##  Groups Name        Variance Std.Dev. Corr 
##  TankID (Intercept) 0.8764   0.9362        
##         logTrial    0.1210   0.3479   -0.75
## Number of obs: 1908, groups:  TankID, 62
## 
## Fixed effects:
##                         Estimate Std. Error z value Pr(>|z|)    
## (Intercept)           -2.4924046  0.5646315  -4.414 1.01e-05 ***
## SpeciesW               0.3964083  0.7980036   0.497    0.619    
## SpeciesRW_F2           0.1853676  0.6815036   0.272    0.786    
## SpeciesWR_F2           0.3544718  0.6870027   0.516    0.606    
## logTrial               1.4123662  0.2132315   6.624 3.50e-11 ***
## ColourRLYellow        -1.2273575  0.2101821  -5.839 5.24e-09 ***
## SpeciesW:logTrial      0.0448856  0.3073021   0.146    0.884    
## SpeciesRW_F2:logTrial  0.0706996  0.2602253   0.272    0.786    
## SpeciesWR_F2:logTrial -0.0005729  0.2582566  -0.002    0.998    
## ---
## Signif. codes:  0 '***' 0.001 '**' 0.01 '*' 0.05 '.' 0.1 ' ' 1
## 
## Correlation of Fixed Effects:
##             (Intr) SpecsW SpRW_F2 SpWR_F2 logTrl ClrRLY SpcW:T SRW_F2:
## SpeciesW    -0.674                                                    
## SpecisRW_F2 -0.791  0.558                                             
## SpecisWR_F2 -0.771  0.553  0.648                                      
## logTrial    -0.890  0.610  0.719   0.702                              
## ColorRLYllw -0.092 -0.016 -0.036  -0.055  -0.099                      
## SpcsW:lgTrl  0.597 -0.901 -0.491  -0.486  -0.656 -0.003               
## SpcsRW_F2:T  0.704 -0.496 -0.905  -0.576  -0.779  0.023  0.534        
## SpcsWR_F2:T  0.698 -0.498 -0.583  -0.900  -0.766 -0.005  0.537  0.634
```

```
## ~~~~~~
# Test significance of random effects
# m2: random intercept only
# m3: random slope only
reversL.m2 <- glmer(Choice ~ Species + logTrial + ColourRL +
                      Species*logTrial +
                      (1|TankID),
                    family = binomial, control = glmerControl("bobyqa"), data = reversLFem2)
reversL.m3 <- glmer(Choice ~ Species + logTrial + ColourRL +
                      Species*logTrial +
                      (0+logTrial|TankID),
                    family = binomial, control = glmerControl("bobyqa"), data = reversLFem2)
anova(reversL.m1.1, reversL.m2) # test inclusion of a random slope
```

```
## Data: reversLFem2
## Models:
## reversL.m2: Choice ~ Species + logTrial + ColourRL + Species * logTrial + 
## reversL.m2:     (1 | TankID)
## reversL.m1.1: Choice ~ Species + logTrial + ColourRL + Species * logTrial + 
## reversL.m1.1:     (logTrial | TankID)
##              Df    AIC    BIC  logLik deviance  Chisq Chi Df Pr(>Chisq)
## reversL.m2   10 2100.3 2155.8 -1040.1   2080.3                         
## reversL.m1.1 12 2100.2 2166.9 -1038.1   2076.2 4.0547      2     0.1317
```

```
anova(reversL.m1.1, reversL.m3) # test inclusion of a random intercept
```

```
## Data: reversLFem2
## Models:
## reversL.m3: Choice ~ Species + logTrial + ColourRL + Species * logTrial + 
## reversL.m3:     (0 + logTrial | TankID)
## reversL.m1.1: Choice ~ Species + logTrial + ColourRL + Species * logTrial + 
## reversL.m1.1:     (logTrial | TankID)
##              Df    AIC    BIC  logLik deviance  Chisq Chi Df Pr(>Chisq)
## reversL.m3   10 2100.5 2156.1 -1040.3   2080.5                         
## reversL.m1.1 12 2100.2 2166.9 -1038.1   2076.2 4.3053      2     0.1162
```

#### Plot group learning curves

```
maxTrialsRL <- max(reversLFem2$Trial) # 60
# re-run the model to be able to plot Trials without log
reversL.m4 <- glmer(Choice ~ Species + log(Trial) + ColourRL +
                      Species*log(Trial) +
                      Species*TrialsLC_AL +
                      (log(Trial)|TankID),
                    family = binomial, control = glmerControl("bobyqa"), data = reversLFem2)
# model group predictions 
reversL.m4_fit <- effect('Species*log(Trial)',reversL.m4, xlevels = maxTrialsRL, na.rm=T) %>% as.data.frame()
reversL.m4_fit$Species <- factor(reversL.m4_fit$Species, levels = c("R", "W", "RW_F2", "WR_F2"))

ggplot()  + 
  geom_ribbon(data = reversL.m4_fit, col = NA, alpha = 0.2, size = 4,
              aes(Trial, NULL, ymin = lower, ymax = upper, fill = Species)) +
  geom_line(data = reversL.m4_fit, aes(Trial, fit, colour = Species, linetype = Species), size = 0.8)  +
  scale_fill_manual(values = treatmentCol[c(1,2,5,6)]) +
  scale_color_manual(values = treatmentCol[c(1,2,5,6)]) +
  scale_linetype_manual(values = c("solid", "solid", "longdash", "dashed")) +
  scale_x_continuous(breaks = seq(0,maxTrialsRL,12), limits = c(0,maxTrialsRL), expand = expansion(mult = c(0, 0))) +
  scale_y_continuous(breaks = seq(0,1,0.2), limits = c(0,1), expand = expansion(mult = c(0, 0))) +
  labs(x = "Trials",y="Success") +
  theme_classic(base_size = 11) + theme(
    panel.background = element_blank(),
    panel.border = element_blank(),
    legend.position = c(0.9, 0.28),
    axis.text = element_text(colour = "black"),
    axis.title.x = element_text(margin = unit(c(3, 0, 0, 0), "mm")),
    axis.title.y = element_text(margin = unit(c(0, 3, 0, 0), "mm"))    )
```

**Figure 4.2.2.3.** Probability of correct choice over trials for females of each species group in the reversal learning task. Lines show predicted model outputs and shaded areas indicate 95% confidence intervals.

#### Plot individual learning curves

```
ggplot()  + 
  binomial_smooth(data = reversLFem2, size = 0.4, colour = "grey80", aes(x = Trial, y = Choice, group = FishID)) +
  geom_ribbon(data = reversL.m4_fit, col = NA, alpha = 0.2, size = 4,
              aes(Trial, NULL, ymin = lower, ymax = upper, fill = Species)) +
  geom_line(data = reversL.m4_fit, aes(Trial, fit, colour = Species, linetype = Species), size = 0.8)  +
  facet_wrap(~ Species) + # Facet wrap by species
  scale_fill_manual(values = treatmentCol[c(1,2,5,6)]) +
  scale_color_manual(values = treatmentCol[c(1,2,5,6)]) +
  scale_linetype_manual(values = c("solid", "dashed", "dashed", "solid")) +
  scale_y_continuous(breaks = seq(0,1,0.2), limits = c(0,1), expand = expansion(mult = c(0, 0))) +
  labs(x = "Trials",y="Success") +
  ggtitle("Associative Learning") +
  theme_classic(base_size = 11) + theme(
    panel.background = element_blank(),
    panel.border = element_blank(),
    legend.position = "none",
    axis.text = element_text(colour = "black"),
    axis.title.x = element_text(margin = unit(c(3, 0, 0, 0), "mm")),
    axis.title.y = element_text(margin = unit(c(0, 3, 0, 0), "mm")),
    plot.title = element_text(hjust = 0))
```

**Figure 4.2.2.4.** Probability of correct choice over trials for females of each species group in the reversal learning task. Coloured lines show predicted model outputs for each species group and shaded areas indicate 95% confidence intervals. Thin grey lines show individual curves.

---

## 4.3 F1, F2, and parental males

### 4.3.1 Associative learning

```
# Count of fish that learnt per species
LCMale %>% 
  group_by(Species) %>%
  summarise( nFail = length(Success_AL) - sum(Success_AL),
             nSuccess = sum(Success_AL),
             nTotal = length(Success_AL),
             propGroup = nSuccess/nTotal)
```

```
## # A tibble: 6 x 5
##   Species nFail nSuccess nTotal propGroup
## * <fct>   <int>    <int>  <int>     <dbl>
## 1 R           7        6     13     0.462
## 2 W           6        8     14     0.571
## 3 RW_F1       4        7     11     0.636
## 4 WR_F1       4        7     11     0.636
## 5 RW_F2       3       10     13     0.769
## 6 WR_F2       4        8     12     0.667
```

```
## ~~~~~~
## TRIALS TO LEARNING CRITERION

# Subset to learners only
learnersAL_Male <- subset(LCMale, Success_AL == 1)
# Response var: Trials to LC (poisson, count data)
# Potential predictors: Species + Colour
LC.m2 <- glm(data = learnersAL_Male, TrialsLC_AL ~ Species + ColourAL, family = "poisson")
Anova(LC.m2)
```

```
## Analysis of Deviance Table (Type II tests)
## 
## Response: TrialsLC_AL
##          LR Chisq Df Pr(>Chisq)   
## Species   15.3771  5   0.008867 **
## ColourAL   7.3669  1   0.006643 **
## ---
## Signif. codes:  0 '***' 0.001 '**' 0.01 '*' 0.05 '.' 0.1 ' ' 1
```

```
summary(LC.m2)
```

```
## 
## Call:
## glm(formula = TrialsLC_AL ~ Species + ColourAL, family = "poisson", 
##     data = learnersAL_Male)
## 
## Deviance Residuals: 
##     Min       1Q   Median       3Q      Max  
## -3.1488  -1.4318  -0.2364   1.1808   3.4934  
## 
## Coefficients:
##                Estimate Std. Error z value Pr(>|z|)    
## (Intercept)     3.15700    0.08422  37.487  < 2e-16 ***
## SpeciesW        0.15638    0.11001   1.422  0.15516    
## SpeciesRW_F1   -0.02005    0.11813  -0.170  0.86520    
## SpeciesWR_F1    0.22762    0.11190   2.034  0.04194 *  
## SpeciesRW_F2   -0.09912    0.11827  -0.838  0.40196    
## SpeciesWR_F2   -0.07392    0.12029  -0.615  0.53888    
## ColourALyellow -0.19691    0.07318  -2.691  0.00713 ** 
## ---
## Signif. codes:  0 '***' 0.001 '**' 0.01 '*' 0.05 '.' 0.1 ' ' 1
## 
## (Dispersion parameter for poisson family taken to be 1)
## 
##     Null deviance: 150.03  on 45  degrees of freedom
## Residual deviance: 120.69  on 39  degrees of freedom
## AIC: 359.84
## 
## Number of Fisher Scoring iterations: 4
```

```
# Post-hoc comparison of groups
summary( glht(LC.m2, mcp(Species = "Tukey")) )
```

```
## 
##   Simultaneous Tests for General Linear Hypotheses
## 
## Multiple Comparisons of Means: Tukey Contrasts
## 
## 
## Fit: glm(formula = TrialsLC_AL ~ Species + ColourAL, family = "poisson", 
##     data = learnersAL_Male)
## 
## Linear Hypotheses:
##                    Estimate Std. Error z value Pr(>|z|)  
## W - R == 0          0.15638    0.11001   1.422   0.7128  
## RW_F1 - R == 0     -0.02005    0.11813  -0.170   1.0000  
## WR_F1 - R == 0      0.22762    0.11190   2.034   0.3215  
## RW_F2 - R == 0     -0.09912    0.11827  -0.838   0.9602  
## WR_F2 - R == 0     -0.07392    0.12029  -0.615   0.9900  
## RW_F1 - W == 0     -0.17643    0.10632  -1.659   0.5574  
## WR_F1 - W == 0      0.07124    0.09935   0.717   0.9798  
## RW_F2 - W == 0     -0.25550    0.10312  -2.478   0.1300  
## WR_F2 - W == 0     -0.23030    0.10653  -2.162   0.2546  
## WR_F1 - RW_F1 == 0  0.24768    0.10788   2.296   0.1945  
## RW_F2 - RW_F1 == 0 -0.07907    0.11089  -0.713   0.9803  
## WR_F2 - RW_F1 == 0 -0.05387    0.11422  -0.472   0.9971  
## RW_F2 - WR_F1 == 0 -0.32674    0.10422  -3.135   0.0211 *
## WR_F2 - WR_F1 == 0 -0.30154    0.10777  -2.798   0.0574 .
## WR_F2 - RW_F2 == 0  0.02520    0.10774   0.234   0.9999  
## ---
## Signif. codes:  0 '***' 0.001 '**' 0.01 '*' 0.05 '.' 0.1 ' ' 1
## (Adjusted p values reported -- single-step method)
```

---

#### Plot trials to learning criterion

```
ggplot(LCMale, aes(x = factor(Species), y = TrialsLC_AL,
               fill = Species, colour = Species)) +
  geom_violin(trim = FALSE, alpha = 0.4, width = 0.9, colour = NA) +
  geom_boxplot(width=0.07, aes(colour = Species), fill = "grey90", outlier.shape = NA) +
  geom_point(position = position_jitter(w = 0.1, h = 0.05), pch = 19, colour = "grey50", size = 1.5) +
  ylab("Trials to learning criterion") +
  xlab("Species group") +
  scale_fill_manual(values = treatmentCol) +
  scale_color_manual(values = treatmentCol) +
  scale_y_continuous(breaks = seq(0,70,10), limits = c(0,70), expand = expansion(mult = c(0, 0))) +
  theme_classic(base_size = 11) + theme(
    panel.background = element_blank(),
    panel.border = element_blank(),
    legend.position="none",
    axis.text = element_text(colour = "black"),
    axis.title.x = element_text(margin = unit(c(3, 0, 0, 0), "mm")),
    axis.title.y = element_text(margin = unit(c(0, 3, 0, 0), "mm")) )
```

**Figure 4.3.1.1.** Trials taken to reach learning criterion in the associative learning task, for males of each species group.

```
## ~~~~~~
## SUCCESS RATE OVER TRIALS

## Some fish had too many null trials, i.e., did not engage in the task during colour training
## Identify individuals that failed to choose in >60% of the trials and remove from learning curve analyses 
keepm <- c("FishID", "Species", "ColourAL")
nullALm <- assocLMale %>%
  group_by(FishID) %>%
  summarise( freqNA = sum(!complete.cases(Choice)),
             nTotal = length(Choice),
             propNA = freqNA/nTotal)
nullALm <- merge(nullALm, subset(infoMale, select = keepm), by = "FishID")
## Get ID of fish that had > 60% trials without choice
nullIndivM <- as.character( nullALm[nullALm$propNA >= 0.6, "FishID"] )
print(paste0("Individuals to exclude: ", nullIndivM))
```

```
## [1] "Individuals to exclude: "
```

```
## >>> NO MALES EXCLUDED

# make NA as wrong choice for learning rate analyses
assocLMale[is.na(assocLMale$Choice), "Choice"] <- 0

# Sample sizes
overview3 <- subset(assocLMale, Trial == 1)
overview3$SpCol <- paste0(overview3$Species, overview3$ColourAL)
table(overview3$Species) # by species group
```

```
## 
##     R     W RW_F1 WR_F1 RW_F2 WR_F2 
##    13    14    11    11    13    12
```

```
table(overview3$SpCol) # by species group & colour
```

```
## 
##        Rred    RW_F1red RW_F1yellow    RW_F2red RW_F2yellow     Ryellow 
##           7           6           5           6           7           6 
##    WR_F1red WR_F1yellow    WR_F2red WR_F2yellow        Wred     Wyellow 
##           5           6           6           6           7           7
```

```
## ~~~~~~
# Success rate AL
# Response var: Choice (binomial, 1 = correct; 0 = incorrect)
# Potential predictors: Species; Colour; Trial; Species*Trial; 
# Exclude Colour*Trial due to low sample size and since colour was balanced across groups
# Random intercept and slope: fish ID
assocLm.m1 <- glmer(Choice ~ Species + ColourAL + Trial +
                     Species*Trial +
                     (Trial|FishID),
                   family = binomial, control = glmerControl("bobyqa"), data = assocLMale)
# Model output
Anova(assocLm.m1)
```

```
## Analysis of Deviance Table (Type II Wald chisquare tests)
## 
## Response: Choice
##                 Chisq Df Pr(>Chisq)    
## Species        5.8665  5    0.31943    
## ColourAL       2.9595  1    0.08537 .  
## Trial         44.5739  1  2.449e-11 ***
## Species:Trial  4.8400  5    0.43572    
## ---
## Signif. codes:  0 '***' 0.001 '**' 0.01 '*' 0.05 '.' 0.1 ' ' 1
```

```
summary(assocLm.m1)
```

```
## Generalized linear mixed model fit by maximum likelihood (Laplace
##   Approximation) [glmerMod]
##  Family: binomial  ( logit )
## Formula: Choice ~ Species + ColourAL + Trial + Species * Trial + (Trial |  
##     FishID)
##    Data: assocLMale
## Control: glmerControl("bobyqa")
## 
##      AIC      BIC   logLik deviance df.resid 
##   2935.6   3027.2  -1451.8   2903.6     2260 
## 
## Scaled residuals: 
##     Min      1Q  Median      3Q     Max 
## -2.3699 -0.9654  0.5248  0.7649  1.5215 
## 
## Random effects:
##  Groups Name        Variance  Std.Dev. Corr
##  FishID (Intercept) 0.1371329 0.37031      
##         Trial       0.0003351 0.01831  0.93
## Number of obs: 2276, groups:  FishID, 74
## 
## Fixed effects:
##                     Estimate Std. Error z value Pr(>|z|)  
## (Intercept)        -0.003953   0.242908  -0.016   0.9870  
## SpeciesW           -0.295136   0.308838  -0.956   0.3393  
## SpeciesRW_F1        0.127779   0.332601   0.384   0.7008  
## SpeciesWR_F1        0.152896   0.331968   0.461   0.6451  
## SpeciesRW_F2        0.221314   0.324811   0.681   0.4956  
## SpeciesWR_F2       -0.200033   0.328534  -0.609   0.5426  
## ColourALyellow     -0.319276   0.185590  -1.720   0.0854 .
## Trial               0.027049   0.011725   2.307   0.0211 *
## SpeciesW:Trial      0.027667   0.015872   1.743   0.0813 .
## SpeciesRW_F1:Trial  0.024676   0.017967   1.373   0.1696  
## SpeciesWR_F1:Trial  0.023027   0.017234   1.336   0.1815  
## SpeciesRW_F2:Trial  0.028968   0.019239   1.506   0.1322  
## SpeciesWR_F2:Trial  0.032900   0.018442   1.784   0.0744 .
## ---
## Signif. codes:  0 '***' 0.001 '**' 0.01 '*' 0.05 '.' 0.1 ' ' 1
```

```
# Test significance of random effects
# m2: random intercept only
# m3: random slope only
assocLm.m2 <- glmer(Choice ~ Species + ColourAL + Trial +
                     Species*Trial +
                     (1|FishID),
                   family = binomial, control = glmerControl("bobyqa"), data = assocLMale)
assocLm.m3 <- glmer(Choice ~ Species + ColourAL + Trial +
                     Species*Trial +
                     (0+Trial|FishID),
                   family = binomial, control = glmerControl("bobyqa"), data = assocLMale)

anova(assocLm.m1, assocLm.m2) # test for inclusion of slope
```

```
## Data: assocLMale
## Models:
## assocLm.m2: Choice ~ Species + ColourAL + Trial + Species * Trial + (1 | 
## assocLm.m2:     FishID)
## assocLm.m1: Choice ~ Species + ColourAL + Trial + Species * Trial + (Trial | 
## assocLm.m1:     FishID)
##            Df    AIC    BIC  logLik deviance  Chisq Chi Df Pr(>Chisq)  
## assocLm.m2 14 2937.4 3017.6 -1454.7   2909.4                           
## assocLm.m1 16 2935.6 3027.2 -1451.8   2903.6 5.8351      2    0.05407 .
## ---
## Signif. codes:  0 '***' 0.001 '**' 0.01 '*' 0.05 '.' 0.1 ' ' 1
```

```
anova(assocLm.m1, assocLm.m3) # test for inclusion of intercept
```

```
## Data: assocLMale
## Models:
## assocLm.m3: Choice ~ Species + ColourAL + Trial + Species * Trial + (0 + 
## assocLm.m3:     Trial | FishID)
## assocLm.m1: Choice ~ Species + ColourAL + Trial + Species * Trial + (Trial | 
## assocLm.m1:     FishID)
##            Df    AIC    BIC  logLik deviance  Chisq Chi Df Pr(>Chisq)   
## assocLm.m3 14 2942.8 3023.0 -1457.4   2914.8                            
## assocLm.m1 16 2935.6 3027.2 -1451.8   2903.6 11.212      2   0.003676 **
## ---
## Signif. codes:  0 '***' 0.001 '**' 0.01 '*' 0.05 '.' 0.1 ' ' 1
```

#### Plot group learning curves

```
maxTrialsALm <- max(assocLMale$Trial) # 40
# model group predictions 
assocLm.m1_fit <- effect('Species*Trial',assocLm.m1, xlevels = maxTrialsALm, na.rm=T) %>% as.data.frame()
assocLm.m1_fit$Species <- factor(assocLm.m1_fit$Species, levels = c("R", "W", "RW_F1", "WR_F1", "RW_F2", "WR_F2"))
ggplot()  + 
  geom_ribbon(data = assocLm.m1_fit, col = NA, alpha = 0.2, size = 4,
              aes(Trial, NULL, ymin = lower, ymax = upper, fill = Species)) +
  geom_line(data = assocLm.m1_fit, aes(Trial, fit, colour = Species, linetype = Species), size = 0.8)  +
  scale_fill_manual(values = treatmentCol) +
  scale_color_manual(values = treatmentCol) +
  scale_linetype_manual(values = c("solid", "solid", "longdash", "longdash", "dashed", "dashed")) +
  scale_x_continuous(breaks = seq(0,maxTrialsALm,5), limits = c(0,maxTrialsALm), expand = expansion(mult = c(0, 0))) +
  scale_y_continuous(breaks = seq(0,1,0.2), limits = c(0,1), expand = expansion(mult = c(0, 0))) +
  labs(x = "Trials",y="Success") +
  theme_classic(base_size = 11) + theme(
    panel.background = element_blank(),
    panel.border = element_blank(),
    axis.text = element_text(colour = "black"),
    axis.title.x = element_text(margin = unit(c(3, 0, 0, 0), "mm")),
    axis.title.y = element_text(margin = unit(c(0, 3, 0, 0), "mm"))    )
```

**Figure 4.3.1.2.** Probability of correct choice over trials for males of each species group in the associative learning task. Lines show predicted model outputs and shaded areas indicate 95% confidence intervals.

#### Plot individual learning curves

```
ggplot()  + 
  binomial_smooth(data = assocLMale, size = 0.4, colour = "grey80", aes(x = Trial, y = Choice, group = FishID)) +
  geom_ribbon(data = assocLm.m1_fit, col = NA, alpha = 0.2, size = 4,
              aes(Trial, NULL, ymin = lower, ymax = upper, fill = Species)) +
  geom_line(data = assocLm.m1_fit, aes(Trial, fit, colour = Species, linetype = Species), size = 0.8)  +
  facet_wrap(~ Species) + # Facet wrap by species
  scale_fill_manual(values = treatmentCol) +
  scale_color_manual(values = treatmentCol) +
  scale_linetype_manual(values = c("solid", "solid", "longdash", "longdash", "dashed", "dashed")) +
  scale_y_continuous(breaks = seq(0,1,0.2), limits = c(0,1), expand = expansion(mult = c(0, 0))) +
  labs(x = "Trials",y="Success") +
  ggtitle("Associative Learning") +
  theme_classic(base_size = 11) + theme(
    panel.background = element_blank(),
    panel.border = element_blank(),
    legend.position = "none",
    axis.text = element_text(colour = "black"),
    axis.title.x = element_text(margin = unit(c(3, 0, 0, 0), "mm")),
    axis.title.y = element_text(margin = unit(c(0, 3, 0, 0), "mm")),
    plot.title = element_text(hjust = 0))
```

**Figure 4.3.1.3.** Probability of correct choice over trials for males of each species group in the associative learning task. Coloured lines show predicted model outputs for each species group and shaded areas indicate 95% confidence intervals. Thin grey lines show individual curves.

### 4.3.2 Test session

```
## ~~~~~~
## TEST IF FISH CHOOSE CORRECT COLOUR WITHOUT TRANSPARENT BLOCK

# read data
testAL <- read.csv("associativeL_Test_F1_F2_males.csv", header = TRUE, 
                         colClasses = c("character", "integer", "integer", "character"))
testAL$FishID <- as.factor(testAL$FishID)

## calculate success rate of each individual in the test (over the 10 trials)
testSuccess <- testAL %>%
  group_by(FishID) %>%
  summarise(successRate = mean(Choice))

## check for normality
hist(testSuccess$successRate, main = "Histogram of success rate during test session",
     xlim = c(0,1), xlab = "Success rate", cex.lab=1, cex.main=1.2)
```

```
shapiro.test(testSuccess$successRate)
```

```
## 
##  Shapiro-Wilk normality test
## 
## data:  testSuccess$successRate
## W = 0.9346, p-value = 0.01236
```

```
## >> not normally distributed so using non-parametric test

## ~~~~~~
## test if the distribution of success rate is significantly greater than chance levels (50%)
## chance levels mean the fish choose the red and yellow holes at random, and 
## greater than chance means the fish choose the holes based on the correct colour
wilcox.test(testSuccess$successRate, mu = 0.5, alternative = "greater")
```

```
## 
##  Wilcoxon signed rank test with continuity correction
## 
## data:  testSuccess$successRate
## V = 986.5, p-value = 4.187e-09
## alternative hypothesis: true location is greater than 0.5
```

```
## bootstrapping to get CI
Bmean <- function(data, i) {
  d <- data[i] # allows boot to select sample
  return(mean(d)) }
## bootstrapping with 1000 replications
results <- boot(data=testSuccess$successRate, statistic=Bmean, R=1000)

## Mean success rate
mean(testSuccess$successRate)*100
```

```
## [1] 78.91304
```

```
## 95% confidence interval
boot.ci(results, type="norm")
```

```
## BOOTSTRAP CONFIDENCE INTERVAL CALCULATIONS
## Based on 1000 bootstrap replicates
## 
## CALL : 
## boot.ci(boot.out = results, type = "norm")
## 
## Intervals : 
## Level      Normal        
## 95%   ( 0.7448,  0.8332 )  
## Calculations and Intervals on Original Scale
```

### 4.3.3 Reversal learning

```
# Count of fish that learnt per species
LCMale %>% 
  drop_na(Success_RL) %>%
  group_by(Species) %>%
  summarise( nFail = length(Success_RL) - sum(Success_RL),
             nSuccess = sum(Success_RL, na.rm = TRUE),
             nTotal = n(),
             propGroup = nSuccess/nTotal)
```

```
## # A tibble: 6 x 5
##   Species nFail nSuccess nTotal propGroup
## * <fct>   <int>    <int>  <int>     <dbl>
## 1 R           5        1      6     0.167
## 2 W           7        1      8     0.125
## 3 RW_F1       4        3      7     0.429
## 4 WR_F1       5        1      6     0.167
## 5 RW_F2       4        6     10     0.6  
## 6 WR_F2       3        4      7     0.571
```

```
## ~~~~~~
## TRIALS TO LEARNING CRITERION

# Sample size too low for statistical comparison of trials to learn
```

#### Plot trials to learning criterion

```
# calculate mean, SD of trials to LC by species
learnersRLm <- LCMale[!is.na(LCMale$TrialsLC_RL),]
meanLC_RLm <- learnersRLm %>%
  group_by(Species) %>%
  summarise( avgLC = mean(TrialsLC_RL),
             sdLC = sd(TrialsLC_RL) )
ggplot(LCMale, aes(x = factor(Species), y = TrialsLC_RL,
               fill = Species, colour = Species)) +
  # geom_violin(trim = FALSE, alpha = 0.4, width = 0.9, colour = NA) +
  # geom_boxplot(width=0.07, aes(colour = Species), fill = "grey90", outlier.shape = NA) +
  geom_point(data = meanLC_RLm, aes(x = Species, y = avgLC, colour = Species), size = 15, pch = "-") +
  geom_point(position = position_jitter(w = 0.1, h = 0.05), aes(colour = Species), pch = 19, size = 1.5) +
  ylab("Trials to learning criterion") +
  xlab("Species group") +
  scale_fill_manual(values = treatmentCol) +
  scale_color_manual(values = treatmentCol) +
  scale_y_continuous(breaks = seq(0,70,10), limits = c(0,70), expand = expansion(mult = c(0, 0))) +
  theme_classic(base_size = 11) + theme(
    panel.background = element_blank(),
    panel.border = element_blank(),
    legend.position="none",
    axis.text = element_text(colour = "black"),
    axis.title.x = element_text(margin = unit(c(3, 0, 0, 0), "mm")),
    axis.title.y = element_text(margin = unit(c(0, 3, 0, 0), "mm")) )
```

**Figure 4.3.3.1.** Trials taken to reach learning criterion in the reversal learning task, for males of each species group.

```
## ~~~~~~
## SUCCESS RATE OVER TRIALS

# make NA as wrong choice for learning rate analyses
reversLMale[is.na(reversLMale$Choice), "Choice"] <- 0

# Sample sizes
overview4 <- subset(reversLMale, Trial == 1)
table(overview4$Species) # by species group
```

```
## 
##     R     W RW_F1 WR_F1 RW_F2 WR_F2 
##     6     8     7     7    10     8
```

```
## ~~~~~~
# Success rate AL
# Response var: Choice (binomial, 1 = correct; 0 = incorrect)
# Potential predictors: Species; Colour; Trial; Species*Trial; Colour*Trial
# Random intercept and slope: fish ID
reversLm.m1 <- glmer(Choice ~ Species + ColourAL + Trial +
                     Species*Trial +
                     (Trial|FishID),
                   family = binomial, control = glmerControl("bobyqa"), data = reversLMale)
# Model output
Anova(reversLm.m1)
```

```
## Analysis of Deviance Table (Type II Wald chisquare tests)
## 
## Response: Choice
##                 Chisq Df Pr(>Chisq)    
## Species        8.7344  5    0.12014    
## ColourAL       6.3297  1    0.01187 *  
## Trial         76.8645  1    < 2e-16 ***
## Species:Trial  1.7611  5    0.88111    
## ---
## Signif. codes:  0 '***' 0.001 '**' 0.01 '*' 0.05 '.' 0.1 ' ' 1
```

```
# Test if log(Trial) improves fit:
reversLMale$logTrial <- log(reversLMale$Trial)
reversLm.m1.1 <- glmer(Choice ~ Species + logTrial + ColourRL +
                        Species*logTrial +
                        (logTrial|FishID),
                      family = binomial, control = glmerControl("bobyqa"), data = reversLMale)
AIC(reversLm.m1, reversLm.m1.1) # log(Trial) improves model fit
```

```
##               df      AIC
## reversLm.m1   16 3196.896
## reversLm.m1.1 16 3192.351
```

```
## ~~~~~~
## Final model output
Anova(reversLm.m1.1)
```

```
## Analysis of Deviance Table (Type II Wald chisquare tests)
## 
## Response: Choice
##                    Chisq Df Pr(>Chisq)    
## Species           5.7231  5    0.33410    
## logTrial         67.7047  1    < 2e-16 ***
## ColourRL          5.5519  1    0.01846 *  
## Species:logTrial  3.0112  5    0.69826    
## ---
## Signif. codes:  0 '***' 0.001 '**' 0.01 '*' 0.05 '.' 0.1 ' ' 1
```

```
summary(reversLm.m1.1)
```

```
## Generalized linear mixed model fit by maximum likelihood (Laplace
##   Approximation) [glmerMod]
##  Family: binomial  ( logit )
## Formula: Choice ~ Species + logTrial + ColourRL + Species * logTrial +  
##     (logTrial | FishID)
##    Data: reversLMale
## Control: glmerControl("bobyqa")
## 
##      AIC      BIC   logLik deviance df.resid 
##   3192.4   3285.2  -1580.2   3160.4     2436 
## 
## Scaled residuals: 
##     Min      1Q  Median      3Q     Max 
## -2.0831 -0.8783 -0.4067  0.8857  2.9606 
## 
## Random effects:
##  Groups Name        Variance Std.Dev. Corr 
##  FishID (Intercept) 1.378    1.1737        
##         logTrial    0.157    0.3962   -0.93
## Number of obs: 2452, groups:  FishID, 46
## 
## Fixed effects:
##                       Estimate Std. Error z value Pr(>|z|)  
## (Intercept)           -1.39424    0.69011  -2.020   0.0434 *
## SpeciesW              -0.63094    0.89211  -0.707   0.4794  
## SpeciesRW_F1          -1.19184    0.94510  -1.261   0.2073  
## SpeciesWR_F1          -0.73455    0.93293  -0.787   0.4311  
## SpeciesRW_F2          -1.00372    0.87128  -1.152   0.2493  
## SpeciesWR_F2           0.49412    0.88845   0.556   0.5781  
## logTrial               0.53665    0.21460   2.501   0.0124 *
## ColourRLyellow        -0.43893    0.18628  -2.356   0.0185 *
## SpeciesW:logTrial      0.18793    0.28640   0.656   0.5117  
## SpeciesRW_F1:logTrial  0.28843    0.30292   0.952   0.3410  
## SpeciesWR_F1:logTrial  0.17808    0.30110   0.591   0.5542  
## SpeciesRW_F2:logTrial  0.29673    0.27968   1.061   0.2887  
## SpeciesWR_F2:logTrial -0.08207    0.29147  -0.282   0.7783  
## ---
## Signif. codes:  0 '***' 0.001 '**' 0.01 '*' 0.05 '.' 0.1 ' ' 1
```

```
# Test significance of random effects
# m2: random intercept only
# m3: random slope only
reversLm.m2 <- glmer(Choice ~ Species + logTrial + ColourRL +
                      Species*logTrial +
                      (1|FishID),
                    family = binomial, control = glmerControl("bobyqa"), data = reversLMale)
reversLm.m3 <- glmer(Choice ~ Species + logTrial + ColourRL +
                      Species*logTrial +
                      (0+logTrial|FishID),
                    family = binomial, control = glmerControl("bobyqa"), data = reversLMale)

anova(reversLm.m1.1, reversLm.m2) # test for inclusion of slope
```

```
## Data: reversLMale
## Models:
## reversLm.m2: Choice ~ Species + logTrial + ColourRL + Species * logTrial + 
## reversLm.m2:     (1 | FishID)
## reversLm.m1.1: Choice ~ Species + logTrial + ColourRL + Species * logTrial + 
## reversLm.m1.1:     (logTrial | FishID)
##               Df    AIC    BIC  logLik deviance  Chisq Chi Df Pr(>Chisq)    
## reversLm.m2   14 3206.6 3287.8 -1589.3   3178.6                             
## reversLm.m1.1 16 3192.4 3285.2 -1580.2   3160.4 18.219      2  0.0001106 ***
## ---
## Signif. codes:  0 '***' 0.001 '**' 0.01 '*' 0.05 '.' 0.1 ' ' 1
```

```
anova(reversLm.m1.1, reversLm.m3) # test for inclusion of intercept
```

```
## Data: reversLMale
## Models:
## reversLm.m3: Choice ~ Species + logTrial + ColourRL + Species * logTrial + 
## reversLm.m3:     (0 + logTrial | FishID)
## reversLm.m1.1: Choice ~ Species + logTrial + ColourRL + Species * logTrial + 
## reversLm.m1.1:     (logTrial | FishID)
##               Df    AIC    BIC  logLik deviance  Chisq Chi Df Pr(>Chisq)   
## reversLm.m3   14 3201.8 3283.0 -1586.9   3173.8                            
## reversLm.m1.1 16 3192.4 3285.2 -1580.2   3160.4 13.415      2   0.001222 **
## ---
## Signif. codes:  0 '***' 0.001 '**' 0.01 '*' 0.05 '.' 0.1 ' ' 1
```

#### Plot group learning curves

```
maxTrialsRLm <- max(reversLMale$Trial) # 60
# re-run the model to be able to plot Trials without log
reversL.m4 <- glmer(Choice ~ Species + log(Trial)+ ColourRL +
                      Species*log(Trial) +
                      (log(Trial)|FishID),
                    family = binomial, control = glmerControl("bobyqa"), data = reversLMale)
# model group predictions 
reversL.m4_fit <- effect('Species*log(Trial)',reversL.m4, xlevels = maxTrialsRLm, na.rm=T) %>% as.data.frame()
reversL.m4_fit$Species <- factor(reversL.m4_fit$Species, levels = c("R", "W", "RW_F1", "WR_F1", "RW_F2", "WR_F2"))

ggplot()  + 
  geom_ribbon(data = reversL.m4_fit, col = NA, alpha = 0.2, size = 4,
              aes(Trial, NULL, ymin = lower, ymax = upper, fill = Species)) +
  geom_line(data = reversL.m4_fit, aes(Trial, fit, colour = Species, linetype = Species), size = 0.8)  +
  scale_fill_manual(values = treatmentCol) +
  scale_color_manual(values = treatmentCol) +
  scale_linetype_manual(values = c("solid", "solid", "longdash", "longdash", "dashed", "dashed")) +
  scale_x_continuous(breaks = seq(0,maxTrialsRLm,12), limits = c(0,maxTrialsRLm), expand = expansion(mult = c(0, 0))) +
  scale_y_continuous(breaks = seq(0,1,0.2), limits = c(0,1), expand = expansion(mult = c(0, 0))) +
  labs(x = "Trials",y="Success") +
  theme_classic(base_size = 11) + theme(
    panel.background = element_blank(),
    panel.border = element_blank(),
    axis.text = element_text(colour = "black"),
    axis.title.x = element_text(margin = unit(c(3, 0, 0, 0), "mm")),
    axis.title.y = element_text(margin = unit(c(0, 3, 0, 0), "mm"))    )
```

**Figure 4.3.3.2.** Probability of correct choice over trials for males of each species group in the reversal learning task. Lines show predicted model outputs and shaded areas indicate 95% confidence intervals.

#### Plot individual learning curves

```
ggplot()  + 
  binomial_smooth(data = reversLMale, size = 0.4, colour = "grey80", aes(x = Trial, y = Choice, group = FishID)) +
  geom_ribbon(data = reversL.m4_fit, col = NA, alpha = 0.2, size = 4,
              aes(Trial, NULL, ymin = lower, ymax = upper, fill = Species)) +
  geom_line(data = reversL.m4_fit, aes(Trial, fit, colour = Species, linetype = Species), size = 0.8)  +
  facet_wrap(~ Species) + # Facet wrap by species
  scale_fill_manual(values = treatmentCol) +
  scale_color_manual(values = treatmentCol) +
  scale_linetype_manual(values = c("solid", "solid", "longdash", "longdash", "dashed", "dashed")) +
  scale_y_continuous(breaks = seq(0,1,0.2), limits = c(0,1), expand = expansion(mult = c(0, 0))) +
  labs(x = "Trials",y="Success") +
  ggtitle("Associative Learning") +
  theme_classic(base_size = 11) + theme(
    panel.background = element_blank(),
    panel.border = element_blank(),
    legend.position = "none",
    axis.text = element_text(colour = "black"),
    axis.title.x = element_text(margin = unit(c(3, 0, 0, 0), "mm")),
    axis.title.y = element_text(margin = unit(c(0, 3, 0, 0), "mm")),
    plot.title = element_text(hjust = 0))
```

**Figure 4.3.3.3.** Probability of correct choice over trials for males of each species group in the reversal learning task. Coloured lines show predicted model outputs for each species group and shaded areas indicate 95% confidence intervals. Thin grey lines show individual curves.

---

# 5 Phenotypic trait variation and transgression

## 5.1 Brain morphospace of F1 hybrids

```
## get dataset from selected group
brainData <- eval(parse(text = "brainsF1"))

## Log transformation of brain variables + log transformation and mean center body length
## ~~~~~~
brainData$mcBodyLen <- log10(brainData$BodyLength) - mean(log10(brainData$BodyLength))
brainData$weightTotBr <- log10(brainData$BrainWeight_mg)
brainData$volTotBr <- log10(brainData$TotalBrainVol)
brainData$tel <- log10(brainData$TelVolTot)
brainData$tel_rest <- log10(brainData$BrainVol_minusTel)
brainData$ot <- log10(brainData$OTVolTot)
brainData$ot_rest <- log10(brainData$BrainVol_minusOT)
brainData$cer <- log10(brainData$CerVolAvg)
brainData$cer_rest <- log10(brainData$BrainVol_minusCer)
brainData$dm <- log10(brainData$DMVolAvg)
brainData$dm_rest <- log10(brainData$BrainVol_minusDM)
brainData$ob <- log10(brainData$OBVolTot)
brainData$ob_rest <- log10(brainData$BrainVol_minusOB)
brainData$hyp <- log10(brainData$HypVolTot)
brainData$hyp_rest <- log10(brainData$BrainVol_minusHyp)

## Get relative brain size residuals & relative Tel/OT size residuals
## ~~~~~~
brainData$RelBrainWeight <- stdres(lm(weightTotBr ~ log10(BodyLength), brainData))
brainData$RelTelVol <- stdres(lm(tel ~ tel_rest, brainData) )
brainData$RelOTVol <- stdres(lm(ot ~ ot_rest, brainData) )

## OPTIONS:
## ~~~~~~
limT = 95 # Run analyses on 95 % kde
## Variables to include in analyses: Total brain weight (rel to body size) + Rel Tel + Rel OT (both rel to brain remainder)
inclVars <- c("RelBrainWeight", "RelTelVol","RelOTVol") 

# get subset data frames
R_subsetDF <- subset(brainData, Species == "R", select = inclVars)
RW_subsetDF <- subset(brainData, Species == "RW", select = inclVars)
WR_subsetDF <- subset(brainData, Species == "WR", select = inclVars)
W_subsetDF <- subset(brainData, Species == "W", select = inclVars)

# Calculate KDEs in 3D
## ~~~~~
kde_R3d <-  kde( R_subsetDF )
kde_RW3d <- kde( RW_subsetDF )
kde_WR3d <- kde( WR_subsetDF )
kde_W3d <-  kde( W_subsetDF )

## Create empty data frame to save KDE volume estimations (phenotypic dispersion), volume ratios, and number/proportion of transgressive individuals per hybrid crossing
## Add a row for data on simulated hybrid populations - simH
## ~~~~~
results_3dKDE <- matrix(nrow = 3, ncol = 7)
colnames(results_3dKDE) <- c("Hybrid", "nTransg", "pTransg", "volKDEratio", "volKDE_Hyb", "volKDE_R", "volKDE_W")
results_3dKDE <- as.data.frame(results_3dKDE)
results_3dKDE$Hybrid <- c("RW", "WR", "SimH")
```

```
## ESTIMATE PHENOTYPIC DISPERSION
## ~~~~~
## Get volumes of the 95% kdes
vol_R <- contourSizes(kde_R3d, cont=limT, approx=T)
vol_W <- contourSizes(kde_W3d, cont=limT, approx=T)
vol_RW <- contourSizes(kde_RW3d, cont=limT, approx=T)
vol_WR <- contourSizes(kde_WR3d, cont=limT, approx=T)
## save to main results table
results_3dKDE$volKDE_R <- vol_R
results_3dKDE$volKDE_W <- vol_W
results_3dKDE[results_3dKDE$Hybrid=="RW", "volKDE_Hyb"] <- vol_RW
results_3dKDE[results_3dKDE$Hybrid=="WR", "volKDE_Hyb"] <- vol_WR
## Calculate ratio of the hypervolume of the hybrid class over the mean parental hypervolume
results_3dKDE[results_3dKDE$Hybrid=="RW", "volKDEratio"] <- vol_RW / ((vol_R + vol_W)/2)
results_3dKDE[results_3dKDE$Hybrid=="WR", "volKDEratio"] <- vol_WR / ((vol_R + vol_W)/2)

## CALCULATE PHENOTYPIC MISMATCH & PARENTAL BIAS
## function externally loaded
## ~~~~~
## RW
transgression_analysis_3d(data = brainData, vars = inclVars, nameP1 = "R", nameP2 = "W", nameH = "RW")
## save as new data frame
transgOutputDF <- transg_func_output
## clean environ
rm(transg_func_output)
## ~~~~~
## WR
transgression_analysis_3d(data = brainData, vars = inclVars, nameP1 = "R", nameP2 = "W", nameH = "WR")
## add to data frame
transgOutputDF <- rbind(transgOutputDF, transg_func_output)
## clean environ
rm(transg_func_output)
## ~~~~~
## Save to main results table
n_transgRW <- transgOutputDF[transgOutputDF$Hybrid_ID == "RW", "nTransgH"]
n_transgWR <- transgOutputDF[transgOutputDF$Hybrid_ID == "WR", "nTransgH"]
results_3dKDE[results_3dKDE$Hybrid == "RW", "nTransg"] <- n_transgRW
results_3dKDE[results_3dKDE$Hybrid == "WR", "nTransg"] <- n_transgWR
results_3dKDE[results_3dKDE$Hybrid == "RW", "pTransg"] <- ( n_transgRW / nrow(RW_subsetDF) )*100
results_3dKDE[results_3dKDE$Hybrid == "WR", "pTransg"] <- ( n_transgWR / nrow(WR_subsetDF) )*100
print(results_3dKDE)
```

```
##   Hybrid nTransg  pTransg volKDEratio volKDE_Hyb volKDE_R volKDE_W
## 1     RW       3 8.823529    4.164262   5.500617 1.152545 1.489276
## 2     WR       2 6.250000    2.993248   3.953812 1.152545 1.489276
## 3   SimH      NA       NA          NA         NA 1.152545 1.489276
```

```
print(transgOutputDF)
```

```
##   Hybrid_ID P1_ID P2_ID nTransgH ParentalBias ParentalMismatch Midpoint_P1P2
## 1        RW     R     W        3   -0.0148540        0.4500377     0.6406483
## 2        WR     R     W        2   -0.2534181        0.3433414     0.6406483
```

```
## SIMULATE HYBRID PHENOTYPES FROM COMPLETE ADDITIVE INHERITANCE OF PHENOTYPE
## ~~~~~
## Function that randomly selects N parental individuals from each species to generate a sample of N simulated hybrids
#   > data: full dataframe used in the KDE the analyses
#   > P1: string with name of parental species 1
#   > P2: string with name of parental species 2
#   > N: pre-defined sample size, default n = 20 to match sample size of real dataset
#   > vars: name of the columns to use for analyses

hybrid_simulation <- function(N, data, P1 = "R", P2 = "W", vars)
{
  ## subset dataset for target species and target variables
  parental1DF <- subset(data, Species == P1, select = vars)
  parental2DF <- subset(data, Species == P2, select = vars)
    ## reset rownames
  rownames(parental1DF) <- NULL
  rownames(parental2DF) <- NULL
  ## ~~~~~
  ## generate the random parental pairs
  parental1_rands <- as.numeric( sample( rownames(parental1DF), size = N, replace = TRUE ) )
  parental2_rands <- as.numeric( sample( rownames(parental2DF), size = N, replace = TRUE ) )
  ## create empty matrix to save simulated hybrid population
  simHybDF <- matrix(ncol = 3, nrow = N)
  colnames(simHybDF) <- vars
  ## ~~~~~
  ## simulate N hybrids as the linear mean of the parental individuals
  for(iHybrid in 1:N) {
    phenotP1 <- parental1DF[parental1_rands[iHybrid], ]
    phenotP2 <- parental2DF[parental2_rands[iHybrid], ]
    ## generate mean hybrid phenotype
    phenotSimH <- colMeans( rbind(phenotP1, phenotP2) )
    ## save to matrix
    simHybDF[iHybrid, ] <- phenotSimH }
  return(simHybDF)
}

## Repeat the simulation N times and save all hybrid populations as a list
## ~~~~~
simNindivs = 20 # number of simulated individuals, to match our real sample size
nSim = 100 # nSim: pre-defined number of simulated runs
## run function
simHybList <- replicate(nSim, hybrid_simulation(N=simNindivs, data=brainData, vars=inclVars), simplify=FALSE)

## Calculate KDE of each simulation
## ~~~~~
simHybKDEs <- lapply(simHybList, kde)

## Get mean phenotype of each hybrid population
## ~~~~~
hybMeans <- lapply(simHybList, colMeans)
simHybMeans <- unlist( lapply(hybMeans, function(x) x[1]) )
names(simHybMeans) <- NULL
simHybMeans <- cbind(simHybMeans, unlist( lapply(hybMeans, function(x) x[2]) ) )
rownames(simHybMeans) <- NULL
simHybMeans <- cbind(simHybMeans, unlist( lapply(hybMeans, function(x) x[3]) ) )
rownames(simHybMeans) <- NULL
colnames(simHybMeans) <- inclVars
## data frame with mean phenotypes
simHybMeans <- as.data.frame(simHybMeans)
```

```
## ESTIMATE PHENOTYPIC DISPERSION OF SIMULATED HYBRIDS
## ~~~~~
## Get volume of 95% kde of each simulated population
simHybVols <- unlist( lapply(simHybKDEs, contourSizes, cont=limT, approx=TRUE) )
## save mean volume of simulated hybrid populations
results_3dKDE[results_3dKDE$Hybrid == "SimH", "volKDE_Hyb"] <- mean(simHybVols)
## Calculate ratio of the hypervolume of the simulated hybrid population over the mean parental hypervolume
results_3dKDE[results_3dKDE$Hybrid=="SimH", "volKDEratio"] <- mean(simHybVols) / ((vol_R + vol_W)/2)

## CALCULATE PHENOTYPIC MISMATCH & PARENTAL BIAS FOR EACH SIMULATED POP
## function externally loaded
## ~~~~~
# reshape from list to long format data frame
test <- melt( as.data.frame(simHybList) )
# add simulation ID to each row: every X rows (number indiv * number vars) is a new simulation
n<-length(inclVars)
simIDs <- sort( rep(seq(1:nSim), (simNindivs*n)) )
test$simPops <- paste0("Sim_", simIDs)
varNames <- c( rep(inclVars[1], simNindivs), rep(inclVars[2], simNindivs), rep(inclVars[3], simNindivs) )
test$variable2 <- rep(varNames, nSim)
## reshape back into three variable columns with the simulation ID as identifyer
allSimHPops <- subset(test, variable2 == inclVars[1])
allSimHPops <- data.frame("Species" = allSimHPops[, "simPops"], "var1" = allSimHPops[, "value"])
allSimHPops$var2 <- test[test$variable2 == inclVars[2], "value"]
allSimHPops$var3 <- test[test$variable2 == inclVars[3], "value"]
colnames(allSimHPops) <- c("Species", inclVars[1], inclVars[2], inclVars[3])
# clean environ
rm(n); rm(test)
## create data frame with parental and simulated hybrid data to feed to function calculating mean phenotype height and position
relBrains_simH <- subset(brainData, select = c("Species", inclVars))
relBrains_simH$Species <- as.character(relBrains_simH$Species)
allSimHPops$Species <- as.character(allSimHPops$Species)
relBrains_simH <- rbind(relBrains_simH, allSimHPops)
rownames(relBrains_simH) <- NULL

## Calculate mismatch and bias for each simulated hybrid population
## ~~~~~
simPops_ids <- unique(allSimHPops$Species)
for (iSimPop in simPops_ids) {
  ## calculate height and position for each simulated pop
  transgression_analysis_3d(data = relBrains_simH, vars = inclVars, nameP1 = "R", nameP2 = "W", nameH = iSimPop)
  ## merge output to main data frame
  transgOutputDF <- rbind(transgOutputDF, transg_func_output)
  rm(transg_func_output)
}
## save to output table
results_3dKDE[results_3dKDE$Hybrid == "SimH", "nTransg"] <- mean(transgOutputDF[3:nrow(transgOutputDF), "nTransgH"])
pTransgH <- transgOutputDF[3:nrow(transgOutputDF), "nTransgH"]/simNindivs
results_3dKDE[results_3dKDE$Hybrid == "SimH", "pTransg"] <- mean(pTransgH)

## print on screen mean & SD of all simulated hybrid pops:
## ~~~~~
## phenotypic dispersion
mean(simHybVols)
```

```
## [1] 0.371671
```

```
sd(simHybVols)
```

```
## [1] 0.1254553
```

```
## proportion of transgressive simulated hybrids
mean(pTransgH)
```

```
## [1] 0.0515
```

```
sd(pTransgH)
```

```
## [1] 0.00857233
```

```
## parental bias 
mean(transgOutputDF[3:nrow(transgOutputDF), "ParentalBias"])
```

```
## [1] -2.585784e-05
```

```
sd(transgOutputDF[3:nrow(transgOutputDF), "ParentalBias"])
```

```
## [1] 0.1292198
```

```
## phenotypic mismatch
mean(transgOutputDF[3:nrow(transgOutputDF), "ParentalMismatch"])
```

```
## [1] 0.1865899
```

```
sd(transgOutputDF[3:nrow(transgOutputDF), "ParentalMismatch"])
```

```
## [1] 0.09964163
```

```
results_3dKDE %>%
    kable(digits = 2, caption = "Table 2. Number and proportion of transgressive individuals and phenotypic dispersion (KDE volumes) in brain morphospace for observed and simulated F1 hybrid populations and parental populations.") %>% 
  kable_styling(c("hover"), full_width=TRUE)
```

Table 2. Number and proportion of transgressive individuals and phenotypic dispersion (KDE volumes) in brain morphospace for observed and simulated F1 hybrid populations and parental populations.

| Hybrid | nTransg | pTransg | volKDEratio | volKDE\_Hyb | volKDE\_R | volKDE\_W |
| --- | --- | --- | --- | --- | --- | --- |
| RW | 3.00 | 8.82 | 4.16 | 5.50 | 1.15 | 1.49 |
| WR | 2.00 | 6.25 | 2.99 | 3.95 | 1.15 | 1.49 |
| SimH | 1.03 | 0.05 | 0.28 | 0.37 | 1.15 | 1.49 |

```
kable(head(transgOutputDF), digits = 2, caption = "Table 3. Number of transgressive individuals, parental bias (distance from the midpoint between the parentals), and phenotypic mismatch (deviation from the line connecting parental mean phenotypes) in brain morphospace for observed and simulated F1 hybrid populations.") %>% 
  kable_styling(c("hover"), full_width=TRUE)
```

Table 3. Number of transgressive individuals, parental bias (distance from the midpoint between the parentals), and phenotypic mismatch (deviation from the line connecting parental mean phenotypes) in brain morphospace for observed and simulated F1 hybrid populations.

| Hybrid\_ID | P1\_ID | P2\_ID | nTransgH | ParentalBias | ParentalMismatch | Midpoint\_P1P2 |
| --- | --- | --- | --- | --- | --- | --- |
| RW | R | W | 3 | -0.01 | 0.45 | 0.64 |
| WR | R | W | 2 | -0.25 | 0.34 | 0.64 |
| Sim\_1 | R | W | 1 | -0.05 | 0.23 | 0.64 |
| Sim\_2 | R | W | 1 | 0.02 | 0.11 | 0.64 |
| Sim\_3 | R | W | 1 | 0.01 | 0.28 | 0.64 |
| Sim\_4 | R | W | 2 | 0.17 | 0.28 | 0.64 |

---

#### Plots 95% KDEs with individual data points

```
## Plot 95% kernels
## ~~~~~
kde_Parents <- kde( brainData %>% 
                      subset(Species %in% c("R", "W"), select = inclVars) )
## Plot RW F1
plot(kde_Parents, drawpoints = FALSE, pch = 18, col.pt = "grey30", cex = 1.2, alphavec = 0.2,
     theta=-45, phi=15, d=4, cex.axis = 0.6, cex.lab = 0.9, cex.main = 1.2, cont = limT, col = "grey50", main = "RW F1",
     ylab = "Rel. Telencephalon vol", xlab = "Rel. Brain Weight", zlab = "Rel. Optic Tectum vol")
plot(kde_R3d, add = TRUE, drawpoints = TRUE, pch = 18, col.pt = treatmentCol[1], cex = 1.2, cont = 0, col = NA)
plot(kde_W3d, add = TRUE, drawpoints = TRUE, pch = 18, col.pt = treatmentCol[2], cex = 1.2, cont = 0)
plot(kde_RW3d, add = TRUE, drawpoints = TRUE, pch = 16, col.pt = treatmentCol[3], cex = 1.2, cont = limT, col = treatmentCol[3])
## Plot WR F1
plot(kde_Parents, drawpoints = FALSE, pch = 18, col.pt = "grey30", cex = 1.2, alphavec = 0.2,
     theta=-45, phi=15, d=4, cex.axis = 0.6, cex.lab = 0.9, cex.main = 1.2, cont = limT, col = "grey50", main = "WR F1",
     ylab = "Rel. Telencephalon vol", xlab = "Rel. Brain Weight", zlab = "Rel. Optic Tectum vol")
plot(kde_R3d, add = TRUE, drawpoints = TRUE, pch = 18, col.pt = treatmentCol[1], cex = 1.2, cont = 0, col = NA)
plot(kde_W3d, add = TRUE, drawpoints = TRUE, pch = 18, col.pt = treatmentCol[2], cex = 1.2, cont = 0)
plot(kde_WR3d, add = TRUE, drawpoints = TRUE, pch = 17, col.pt = treatmentCol[4], cex = 1.2, cont = limT, col = treatmentCol[4])
## ~~~~~
## Plot mean phenotypes
# simulated hybrids
scatter3D(simHybMeans[,1],simHybMeans[,2], simHybMeans[,3], alpha = 0.1,
          theta=-45, phi=15, d=4, bty = "b2",  type = "h", col = simHCol,
          xlim = c(-1, 1), ylim = c(-1, 1), zlim = c(-1, 1),
          ticktype = "detailed", pch = 18, cex = 1.5, cex.axis = 0.6, cex.lab = 0.9, cex.main = 1.2, main = "Mean phenotypes", xlab = "Rel. Brain Weight",  ylab = "Rel. Telencephalon vol", zlab = "Rel. Optic Tectum vol")
# R
scatter3D(colMeans(R_subsetDF)[1], colMeans(R_subsetDF)[2], colMeans(R_subsetDF)[3],
          add = TRUE,  type = "h", col = treatmentCol[1],
          ticktype = "detailed", pch = 18, cex = 1.5)
# W
scatter3D(colMeans(W_subsetDF)[1], colMeans(W_subsetDF)[2], colMeans(W_subsetDF)[3],
          add = TRUE,  type = "h", col = treatmentCol[2],
          ticktype = "detailed", pch = 18, cex = 1.5)
# Hybrids
scatter3D(colMeans(RW_subsetDF)[1], colMeans(RW_subsetDF)[2], colMeans(RW_subsetDF)[3],
          add = TRUE,  type = "h", col = treatmentCol[3],
          ticktype = "detailed", pch = 16, cex = 1.5)
scatter3D(colMeans(WR_subsetDF)[1], colMeans(WR_subsetDF)[2], colMeans(WR_subsetDF)[3],
          add = TRUE,  type = "h", col = treatmentCol[4],
          ticktype = "detailed", pch = 17, cex = 1.5)
```

**Figure 5.1.1.** Distribution of individual F1 hybrids and parentals in the brain morphospace, with shaded 95% KDEs (a,b). Position of the mean phenotype of F1 hybrid and parental groups and of simulated hybrid populations (c).

---

#### Plots phenotypic dispersion, mismatch, and parental bias

```
## Hypervolume (phenotypic dispersion)
## ~~~~~
# RW
plot(x = 1, y = results_3dKDE$volKDE_Hyb[1], col = 1, bg = treatmentCol[3], pch = 21, cex = 1.3, 
     ylim = c(0, 6), xlim = c(0.9, 1.1), ylab = "Hypervolume", xlab = NA, xaxt='n')
# simulated pops
points(x = jitter(rep(1, nSim)), y = simHybVols, col = rgb(.7,.7,.7,alpha=0.5), pch = 16, cex = 1)
# WR
points(x = 1, y = results_3dKDE$volKDE_Hyb[2], col = 1, bg = treatmentCol[4], pch = 24, cex = 1.3)
# R
points(x = 1, y = results_3dKDE$volKDE_R[1], col = 1, bg = treatmentCol[1], pch = 23, cex = 1.3)
# W
points(x = jitter(1), y = results_3dKDE$volKDE_W[1], col = 1, bg = treatmentCol[2], pch = 23, cex = 1.3)

## Parental bias
## ~~~~~
# simulated pops
plot(x = jitter(rep(1, nSim)), y = transgOutputDF$ParentalBias[3:nrow(transgOutputDF)], col = rgb(.7,.7,.7,alpha=0.5), pch = 16, cex = 1, ylim = c(-0.6, 0.6), xlim = c(0.9, 1.1), ylab = "Parental bias", xlab = NA, xaxt='n')
# RW
points(x = 1, y = transgOutputDF$ParentalBias[1], col = 1, bg = treatmentCol[3], pch = 21, cex = 1.3)
# WR
points(x = 1, y = transgOutputDF$ParentalBias[2], col = 1, bg = treatmentCol[4], pch = 24, cex = 1.3)
# text
text(0.96, -0.62, "closer to W", cex=0.7, col="grey50", pos=3)
text(0.96, 0.65, "closer to R", cex=0.7, col="grey50", pos=1)

# Phenotypic mismatch
## ~~~~~
# simulated pops
plot(x = jitter(rep(1, nSim)), y = transgOutputDF$ParentalMismatch[3:nrow(transgOutputDF)], col = rgb(.7,.7,.7,alpha=0.5), pch = 16, cex = 1, ylim = c(0, 0.8), xlim = c(0.9, 1.1), ylab = "Phenotypic mismatch", xlab = NA, xaxt='n')
# RW
points(x = 1, y = transgOutputDF$ParentalMismatch[1], col = 1, bg = treatmentCol[3], pch = 21, cex = 1.3)
# WR
points(x = 1, y = transgOutputDF$ParentalMismatch[2], col = 1, bg = treatmentCol[4], pch = 24, cex = 1.3)
```

**Figure 5.1.2.** Phenotypic dispersion (a), parental bias (b), and phenotypic mismatch (c) of observed and simulated F1 hybrid phenotypes in relation to observed parental phenotypes.

## 5.2 Brain morphospace of F2 hybrids

```
## get dataset from selected group
brainData <- eval(parse(text = "brainsF2"))

## Log transformation of brain variables + log transformation and mean center body length
## ~~~~~~
brainData$mcBodyLen <- log10(brainData$BodyLength) - mean(log10(brainData$BodyLength))
brainData$weightTotBr <- log10(brainData$BrainWeight_mg)
brainData$volTotBr <- log10(brainData$TotalBrainVol)
brainData$tel <- log10(brainData$TelVolTot)
brainData$tel_rest <- log10(brainData$BrainVol_minusTel)
brainData$ot <- log10(brainData$OTVolTot)
brainData$ot_rest <- log10(brainData$BrainVol_minusOT)
brainData$cer <- log10(brainData$CerVolAvg)
brainData$cer_rest <- log10(brainData$BrainVol_minusCer)
brainData$dm <- log10(brainData$DMVolAvg)
brainData$dm_rest <- log10(brainData$BrainVol_minusDM)
brainData$ob <- log10(brainData$OBVolTot)
brainData$ob_rest <- log10(brainData$BrainVol_minusOB)
brainData$hyp <- log10(brainData$HypVolTot)
brainData$hyp_rest <- log10(brainData$BrainVol_minusHyp)

## Get relative brain size residuals & relative Tel/OT size residuals
## ~~~~~~
brainData$RelBrainWeight <- stdres(lm(weightTotBr ~ log10(BodyLength), brainData))
brainData$RelTelVol <- stdres(lm(tel ~ tel_rest, brainData) )
brainData$RelOTVol <- stdres(lm(ot ~ ot_rest, brainData) )

## OPTIONS:
## ~~~~~~
limT = 95 # Run analyses on 95 % kde
## Variables to include in analyses: Total brain weight (rel to body size) + Rel Tel + Rel OT (both rel to brain remainder)
inclVars <- c("RelBrainWeight", "RelTelVol","RelOTVol") 

# get subset data frames
R_subsetDF <- subset(brainData, Species == "R", select = inclVars)
RW_subsetDF <- subset(brainData, Species == "RW_F2", select = inclVars)
WR_subsetDF <- subset(brainData, Species == "WR_F2", select = inclVars)
W_subsetDF <- subset(brainData, Species == "W", select = inclVars)

# Calculate KDEs in 3D
## ~~~~~
kde_R3d <-  kde( R_subsetDF )
kde_RW3d <- kde( RW_subsetDF )
kde_WR3d <- kde( WR_subsetDF )
kde_W3d <-  kde( W_subsetDF )

## Create empty data frame to save KDE volume estimations (phenotypic dispersion), volume ratios, 
## and number/proportion of transgressive individuals per hybrid crossing
## Add a row for data on simulated hybrid populations - simH
## ~~~~~
results_3dKDE <- matrix(nrow = 3, ncol = 7)
colnames(results_3dKDE) <- c("Hybrid", "nTransg", "pTransg", "volKDEratio", "volKDE_Hyb", "volKDE_R", "volKDE_W")
results_3dKDE <- as.data.frame(results_3dKDE)
results_3dKDE$Hybrid <- c("RW_F2", "WR_F2", "SimH")
```

```
## ESTIMATE PHENOTYPIC DISPERSION
## ~~~~~
## Get volumes of the 95% kdes
vol_R <- contourSizes(kde_R3d, cont=limT, approx=T)
vol_W <- contourSizes(kde_W3d, cont=limT, approx=T)
vol_RW <- contourSizes(kde_RW3d, cont=limT, approx=T)
vol_WR <- contourSizes(kde_WR3d, cont=limT, approx=T)
## save to main results table
results_3dKDE$volKDE_R <- vol_R
results_3dKDE$volKDE_W <- vol_W
results_3dKDE[results_3dKDE$Hybrid=="RW_F2", "volKDE_Hyb"] <- vol_RW
results_3dKDE[results_3dKDE$Hybrid=="WR_F2", "volKDE_Hyb"] <- vol_WR
## Calculate ratio of the hypervolume of the hybrid class over the mean parental hypervolume
results_3dKDE[results_3dKDE$Hybrid=="RW_F2", "volKDEratio"] <- vol_RW / ((vol_R + vol_W)/2)
results_3dKDE[results_3dKDE$Hybrid=="WR_F2", "volKDEratio"] <- vol_WR / ((vol_R + vol_W)/2)

## CALCULATE PHENOTYPIC MISMATCH & PARENTAL BIAS
## function externally loaded
## ~~~~~
## RW
transgression_analysis_3d(data = brainData, vars = inclVars, nameP1 = "R", nameP2 = "W", nameH = "RW_F2")
## save as new data frame
transgOutputDF <- transg_func_output
## clean environ
rm(transg_func_output)
## ~~~~~
## WR
transgression_analysis_3d(data = brainData, vars = inclVars, nameP1 = "R", nameP2 = "W", nameH = "WR_F2")
## add to data frame
transgOutputDF <- rbind(transgOutputDF, transg_func_output)
## clean environ
rm(transg_func_output)
## ~~~~~
## Save to main results table
n_transgRW <- transgOutputDF[transgOutputDF$Hybrid_ID == "RW_F2", "nTransgH"]
n_transgWR <- transgOutputDF[transgOutputDF$Hybrid_ID == "WR_F2", "nTransgH"]
results_3dKDE[results_3dKDE$Hybrid == "RW_F2", "nTransg"] <- n_transgRW
results_3dKDE[results_3dKDE$Hybrid == "WR_F2", "nTransg"] <- n_transgWR
results_3dKDE[results_3dKDE$Hybrid == "RW_F2", "pTransg"] <- ( n_transgRW / nrow(RW_subsetDF) )*100
results_3dKDE[results_3dKDE$Hybrid == "WR_F2", "pTransg"] <- ( n_transgWR / nrow(WR_subsetDF) )*100
print(results_3dKDE)
```

```
##   Hybrid nTransg  pTransg volKDEratio volKDE_Hyb  volKDE_R volKDE_W
## 1  RW_F2       2 5.128205    1.687948   5.518709 0.8429494 5.696007
## 2  WR_F2       3 8.333333    1.365486   4.464428 0.8429494 5.696007
## 3   SimH      NA       NA          NA         NA 0.8429494 5.696007
```

```
print(transgOutputDF)
```

```
##   Hybrid_ID P1_ID P2_ID nTransgH ParentalBias ParentalMismatch Midpoint_P1P2
## 1     RW_F2     R     W        2   -0.0333041        0.1567676     0.3456275
## 2     WR_F2     R     W        3   -0.4145709        0.7268436     0.3456275
```

```
## SIMULATE HYBRID PHENOTYPES FROM COMPLETE ADDITIVE INHERITANCE OF PHENOTYPE
## ~~~~~
## Function that randomly selects N parental individuals from each species to generate a sample of N simulated hybrids
#   > data: full dataframe used in the KDE the analyses
#   > P1: string with name of parental species 1
#   > P2: string with name of parental species 2
#   > N: pre-defined sample size, default n = 20 to match sample size of real dataset
#   > vars: name of the columns to use for analyses

hybrid_simulation <- function(N, data, P1 = "R", P2 = "W", vars)
{
  ## subset dataset for target species and target variables
  parental1DF <- subset(data, Species == P1, select = vars)
  parental2DF <- subset(data, Species == P2, select = vars)
    ## reset rownames
  rownames(parental1DF) <- NULL
  rownames(parental2DF) <- NULL
  ## ~~~~~
  ## generate the random parental pairs
  parental1_rands <- as.numeric( sample( rownames(parental1DF), size = N, replace = TRUE ) )
  parental2_rands <- as.numeric( sample( rownames(parental2DF), size = N, replace = TRUE ) )
  ## create empty matrix to save simulated hybrid population
  simHybDF <- matrix(ncol = 3, nrow = N)
  colnames(simHybDF) <- vars
  ## ~~~~~
  ## simulate N hybrids as the linear mean of the parental individuals
  for(iHybrid in 1:N) {
    phenotP1 <- parental1DF[parental1_rands[iHybrid], ]
    phenotP2 <- parental2DF[parental2_rands[iHybrid], ]
    ## generate mean hybrid phenotype
    phenotSimH <- colMeans( rbind(phenotP1, phenotP2) )
    ## save to matrix
    simHybDF[iHybrid, ] <- phenotSimH }
  return(simHybDF)
}

## Repeat the simulation N times and save all hybrid populations as a list
## ~~~~~
simNindivs = 20 # number of simulated individuals, to match our real sample size
nSim = 100 # nSim: pre-defined number of simulated runs
## run function
simHybList <- replicate(nSim, hybrid_simulation(N=simNindivs, data=brainData, vars=inclVars), simplify=FALSE)

## Calculate KDE of each simulation
## ~~~~~
simHybKDEs <- lapply(simHybList, kde)

## Get mean phenotype of each hybrid population
## ~~~~~
hybMeans <- lapply(simHybList, colMeans)
simHybMeans <- unlist( lapply(hybMeans, function(x) x[1]) )
names(simHybMeans) <- NULL
simHybMeans <- cbind(simHybMeans, unlist( lapply(hybMeans, function(x) x[2]) ) )
rownames(simHybMeans) <- NULL
simHybMeans <- cbind(simHybMeans, unlist( lapply(hybMeans, function(x) x[3]) ) )
rownames(simHybMeans) <- NULL
colnames(simHybMeans) <- inclVars
## data frame with mean phenotypes
simHybMeans <- as.data.frame(simHybMeans)
```

```
## ESTIMATE PHENOTYPIC DISPERSION OF SIMULATED HYBRIDS
## ~~~~~
## Get volume of 95% kde of each simulated population
simHybVols <- unlist( lapply(simHybKDEs, contourSizes, cont=limT, approx=TRUE) )
## save mean volume of simulated hybrid populations
results_3dKDE[results_3dKDE$Hybrid == "SimH", "volKDE_Hyb"] <- mean(simHybVols)
## Calculate ratio of the hypervolume of the simulated hybrid population over the mean parental hypervolume
results_3dKDE[results_3dKDE$Hybrid=="SimH", "volKDEratio"] <- mean(simHybVols) / ((vol_R + vol_W)/2)

## CALCULATE PHENOTYPIC MISMATCH & PARENTAL BIAS FOR EACH SIMULATED POP
## function externally loaded
## ~~~~~
# reshape from list to long format data frame
test <- melt( as.data.frame(simHybList) )
# add simulation ID to each row: every X rows (number indiv * number vars) is a new simulation
n<-length(inclVars)
simIDs <- sort( rep(seq(1:nSim), (simNindivs*n)) )
test$simPops <- paste0("Sim_", simIDs)
varNames <- c( rep(inclVars[1], simNindivs), rep(inclVars[2], simNindivs), rep(inclVars[3], simNindivs) )
test$variable2 <- rep(varNames, nSim)
## reshape back into three variable columns with the simulation ID as identifyer
allSimHPops <- subset(test, variable2 == inclVars[1])
allSimHPops <- data.frame("Species" = allSimHPops[, "simPops"], "var1" = allSimHPops[, "value"])
allSimHPops$var2 <- test[test$variable2 == inclVars[2], "value"]
allSimHPops$var3 <- test[test$variable2 == inclVars[3], "value"]
colnames(allSimHPops) <- c("Species", inclVars[1], inclVars[2], inclVars[3])
# clean environ
rm(n); rm(test)
## create data frame with parental and simulated hybrid data to feed to function calculating mean phenotype mismatch and bias
relBrains_simH <- subset(brainData, select = c("Species", inclVars))
relBrains_simH$Species <- as.character(relBrains_simH$Species)
allSimHPops$Species <- as.character(allSimHPops$Species)
relBrains_simH <- rbind(relBrains_simH, allSimHPops)
rownames(relBrains_simH) <- NULL

## Calculate mismatch and bias for each simulated hybrid population
## ~~~~~
simPops_ids <- unique(allSimHPops$Species)
for (iSimPop in simPops_ids) {
  ## calculate height and position for each simulated pop
  transgression_analysis_3d(data = relBrains_simH, vars = inclVars, nameP1 = "R", nameP2 = "W", nameH = iSimPop)
  ## merge output to main data frame
  transgOutputDF <- rbind(transgOutputDF, transg_func_output)
  rm(transg_func_output)
}
## save to output table
results_3dKDE[results_3dKDE$Hybrid == "SimH", "nTransg"] <- mean(transgOutputDF[3:nrow(transgOutputDF), "nTransgH"])
pTransgH <- transgOutputDF[3:nrow(transgOutputDF), "nTransgH"]/simNindivs
results_3dKDE[results_3dKDE$Hybrid == "SimH", "pTransg"] <- mean(pTransgH)

## print on screen mean & SD of all simulated hybrid pops:
## ~~~~~
## phenotypic dispersion
mean(simHybVols)
```

```
## [1] 0.6517074
```

```
sd(simHybVols)
```

```
## [1] 0.2790556
```

```
## proportion of transgressive simulated hybrids
mean(pTransgH)
```

```
## [1] 0.0535
```

```
sd(pTransgH)
```

```
## [1] 0.01914195
```

```
## parental bias 
mean(transgOutputDF[3:nrow(transgOutputDF), "ParentalBias"])
```

```
## [1] 0.0004270208
```

```
sd(transgOutputDF[3:nrow(transgOutputDF), "ParentalBias"])
```

```
## [1] 0.1252289
```

```
## phenotypic mismatch
mean(transgOutputDF[3:nrow(transgOutputDF), "ParentalMismatch"])
```

```
## [1] 0.2007647
```

```
sd(transgOutputDF[3:nrow(transgOutputDF), "ParentalMismatch"])
```

```
## [1] 0.1055932
```

```
results_3dKDE %>%
    kable(digits = 2, caption = "Table 4. Number and proportion of transgressive individuals and phenotypic dispersion (KDE volumes) in brain morphospace for observed and simulated F2 hybrid populations and parental populations.") %>% 
  kable_styling(c("hover"), full_width=TRUE)
```

Table 4. Number and proportion of transgressive individuals and phenotypic dispersion (KDE volumes) in brain morphospace for observed and simulated F2 hybrid populations and parental populations.

| Hybrid | nTransg | pTransg | volKDEratio | volKDE\_Hyb | volKDE\_R | volKDE\_W |
| --- | --- | --- | --- | --- | --- | --- |
| RW\_F2 | 2.00 | 5.13 | 1.69 | 5.52 | 0.84 | 5.7 |
| WR\_F2 | 3.00 | 8.33 | 1.37 | 4.46 | 0.84 | 5.7 |
| SimH | 1.07 | 0.05 | 0.20 | 0.65 | 0.84 | 5.7 |

```
kable(head(transgOutputDF), digits = 2, caption = "Table 5. Number of transgressive individuals, parental bias (distance from the midpoint between the parentals), and phenotypic mismatch (deviation from the line connecting parental mean phenotypes) in brain morphospace for observed and simulated F2 hybrid populations.") %>% 
  kable_styling(c("hover"), full_width=TRUE)
```

Table 5. Number of transgressive individuals, parental bias (distance from the midpoint between the parentals), and phenotypic mismatch (deviation from the line connecting parental mean phenotypes) in brain morphospace for observed and simulated F2 hybrid populations.

| Hybrid\_ID | P1\_ID | P2\_ID | nTransgH | ParentalBias | ParentalMismatch | Midpoint\_P1P2 |
| --- | --- | --- | --- | --- | --- | --- |
| RW\_F2 | R | W | 2 | -0.03 | 0.16 | 0.35 |
| WR\_F2 | R | W | 3 | -0.41 | 0.73 | 0.35 |
| Sim\_1 | R | W | 1 | 0.07 | 0.10 | 0.35 |
| Sim\_2 | R | W | 4 | 0.16 | 0.29 | 0.35 |
| Sim\_3 | R | W | 1 | 0.08 | 0.09 | 0.35 |
| Sim\_4 | R | W | 1 | 0.07 | 0.17 | 0.35 |

---

#### Plots 95% KDEs with individual data points

```
## Plot 95% kernels
## ~~~~~
kde_Parents <- kde( brainData %>% 
                      subset(Species %in% c("R", "W"), select = inclVars) )
## Plot RW F2
plot(kde_Parents, drawpoints = FALSE, pch = 18, col.pt = "grey30", cex = 1.2, alphavec = 0.2,
     theta=-45, phi=15, d=4, cex.axis = 0.6, cex.lab = 0.9, cex.main = 1.2, cont = limT, col = "grey50", main = "RW F2",
     ylab = "Rel. Telencephalon vol", xlab = "Rel. Brain Weight", zlab = "Rel. Optic Tectum vol")
plot(kde_R3d, add = TRUE, drawpoints = TRUE, pch = 18, col.pt = treatmentCol[1], cex = 1.2, cont = 0, col = NA)
plot(kde_W3d, add = TRUE, drawpoints = TRUE, pch = 18, col.pt = treatmentCol[2], cex = 1.2, cont = 0)
plot(kde_RW3d, add = TRUE, drawpoints = TRUE, pch = 16, col.pt = treatmentCol[5], cex = 1.2, cont = limT, col = treatmentCol[5])
## Plot WR F2
plot(kde_Parents, drawpoints = FALSE, pch = 18, col.pt = "grey30", cex = 1.2, alphavec = 0.2,
     theta=-45, phi=15, d=4, cex.axis = 0.6, cex.lab = 0.9, cex.main = 1.2, cont = limT, col = "grey50", main = "WR F2",
     ylab = "Rel. Telencephalon vol", xlab = "Rel. Brain Weight", zlab = "Rel. Optic Tectum vol")
plot(kde_R3d, add = TRUE, drawpoints = TRUE, pch = 18, col.pt = treatmentCol[1], cex = 1.2, cont = 0, col = NA)
plot(kde_W3d, add = TRUE, drawpoints = TRUE, pch = 18, col.pt = treatmentCol[2], cex = 1.2, cont = 0)
plot(kde_WR3d, add = TRUE, drawpoints = TRUE, pch = 17, col.pt = treatmentCol[6], cex = 1.2, cont = limT, col = treatmentCol[6])
## ~~~~~
## Plot mean phenotypes
# simulated hybrids
scatter3D(simHybMeans[,1],simHybMeans[,2], simHybMeans[,3], alpha = 0.1,
          theta=-45, phi=15, d=4, bty = "b2",  type = "h", col = simHCol,
          xlim = c(-1, 1), ylim = c(-1, 1), zlim = c(-1, 1),
          ticktype = "detailed", pch = 18, cex = 1.5, cex.axis = 0.6, cex.lab = 0.9, cex.main = 1.2, 
          main = "Mean phenotypes", xlab = "Rel. Brain Weight",  ylab = "Rel. Telencephalon vol", zlab = "Rel. Optic Tectum vol")
# R
scatter3D(colMeans(R_subsetDF)[1], colMeans(R_subsetDF)[2], colMeans(R_subsetDF)[3],
          add = TRUE,  type = "h", col = treatmentCol[1],
          ticktype = "detailed", pch = 18, cex = 1.5)
# W
scatter3D(colMeans(W_subsetDF)[1], colMeans(W_subsetDF)[2], colMeans(W_subsetDF)[3],
          add = TRUE,  type = "h", col = treatmentCol[2],
          ticktype = "detailed", pch = 18, cex = 1.5)
# Hybrids
scatter3D(colMeans(RW_subsetDF)[1], colMeans(RW_subsetDF)[2], colMeans(RW_subsetDF)[3],
          add = TRUE,  type = "h", col = treatmentCol[5],
          ticktype = "detailed", pch = 16, cex = 1.5)
scatter3D(colMeans(WR_subsetDF)[1], colMeans(WR_subsetDF)[2], colMeans(WR_subsetDF)[3],
          add = TRUE,  type = "h", col = treatmentCol[6],
          ticktype = "detailed", pch = 17, cex = 1.5)
```

**Figure 5.2.1.** Distribution of individual F2 hybrids and parentals in the brain morphospace, with shaded 95% KDEs (a,b). Position of the mean phenotype of F2 hybrid and parental groups and of simulated hybrid populations (c).

---

#### Plots phenotypic dispersion, mismatch, and parental bias

```
## Hypervolume (phenotypic dispersion)
## ~~~~~
# RW
plot(x = 1, y = results_3dKDE$volKDE_Hyb[1], col = 1, bg = treatmentCol[5], pch = 21, cex = 1.3, 
     ylim = c(0, 6), xlim = c(0.9, 1.1), ylab = "Hypervolume", xlab = NA, xaxt='n')
# simulated pops
points(x = jitter(rep(1, nSim)), y = simHybVols, col = rgb(.7,.7,.7,alpha=0.5), pch = 16, cex = 1)
# WR
points(x = 1, y = results_3dKDE$volKDE_Hyb[2], col = 1, bg = treatmentCol[6], pch = 24, cex = 1.3)
# R
points(x = 1, y = results_3dKDE$volKDE_R[1], col = 1, bg = treatmentCol[1], pch = 23, cex = 1.3)
# W
points(x = jitter(1), y = results_3dKDE$volKDE_W[1], col = 1, bg = treatmentCol[2], pch = 23, cex = 1.3)

## Parental bias
## ~~~~~
# simulated pops
plot(x = jitter(rep(1, nSim)), y = transgOutputDF$ParentalBias[3:nrow(transgOutputDF)], col = rgb(.7,.7,.7,alpha=0.5), pch = 16, cex = 1, ylim = c(-0.6, 0.6), xlim = c(0.9, 1.1), ylab = "Parental bias", xlab = NA, xaxt='n')
# RW
points(x = 1, y = transgOutputDF$ParentalBias[1], col = 1, bg = treatmentCol[5], pch = 21, cex = 1.3)
# WR
points(x = 1, y = transgOutputDF$ParentalBias[2], col = 1, bg = treatmentCol[6], pch = 24, cex = 1.3)
# text
text(0.96, -0.62, "closer to W", cex=0.7, col="grey50", pos=3)
text(0.96, 0.65, "closer to R", cex=0.7, col="grey50", pos=1)

# Phenotypic mismatch
## ~~~~~
# simulated pops
plot(x = jitter(rep(1, nSim)), y = transgOutputDF$ParentalMismatch[3:nrow(transgOutputDF)], col = rgb(.7,.7,.7,alpha=0.5), pch = 16, cex = 1, ylim = c(0, 0.8), xlim = c(0.9, 1.1), ylab = "Phenotypic mismatch", xlab = NA, xaxt='n')
# RW
points(x = 1, y = transgOutputDF$ParentalMismatch[1], col = 1, bg = treatmentCol[5], pch = 21, cex = 1.3)
# WR
points(x = 1, y = transgOutputDF$ParentalMismatch[2], col = 1, bg = treatmentCol[6], pch = 24, cex = 1.3)
```

**Figure 5.2.2.** Phenotypic dispersion (a), parental bias (b), and phenotypic mismatch (c) of observed and simulated F2 hybrid phenotypes in relation to observed parental phenotypes.

## 5.3 Cognitive space of F1 females

Detailed methods and analyses for F1 female fish can be found in Vila Pouca et al. 2022 Am Nat.

---

## 5.4 Cognitive space of F2 females

```
## OPTIONS:
## ~~~~~~
limT = 95 # Run analyses on 95 % kde

## Learning criterion data for each task; get only complete cases of both tasks
CognSpace <- LCFem2 %>%
  drop_na(TrialsLC_AL, TrialsLC_RL)
CognSpace$FishID <- factor(CognSpace$FishID)
## log transform Trials to LC
CognSpace$logTrials_assoc <- log(CognSpace$TrialsLC_AL)
CognSpace$logTrials_rev <- log(CognSpace$TrialsLC_RL)

## subset data frames
inclVars <- c("logTrials_assoc", "logTrials_rev")

R_subsetDF <- subset(CognSpace, Species == "R", select = inclVars)
RW2_subsetDF <- subset(CognSpace, Species == "RW_F2", select = inclVars)
WR2_subsetDF <- subset(CognSpace, Species == "WR_F2", select = inclVars)
W_subsetDF <- subset(CognSpace, Species == "W", select = inclVars)

# Calculate KDEs in 3D
## ~~~~~
kde_R <-  kde( R_subsetDF )
kde_RW2 <- kde( RW2_subsetDF )
kde_WR2 <- kde( WR2_subsetDF )
kde_W <-  kde( W_subsetDF )

## Create empty data frame to save KDE volume estimations (phenotypic dispersion), volume ratios, and number/proportion of transgressive individuals per hybrid crossing
## Add a row for data on simulated hybrid populations - simH
## ~~~~~
results_KDE <- matrix(nrow = 3, ncol = 7)
colnames(results_KDE) <- c("Hybrid", "nTransg", "pTransg", "volKDEratio", "volKDE_Hyb", "volKDE_R", "volKDE_W")
results_KDE <- as.data.frame(results_KDE)
results_KDE$Hybrid <- c("RW_F2", "WR_F2", "SimH")
```

```
## ESTIMATE PHENOTYPIC DISPERSION
## ~~~~~
vol_R <- contourSizes(kde_R, cont=limT, approx=T)
vol_W <- contourSizes(kde_W, cont=limT, approx=T)
vol_RW2 <- contourSizes(kde_RW2, cont=limT, approx=T)
vol_WR2 <- contourSizes(kde_WR2, cont=limT, approx=T)
## save to results table
results_KDE$volKDE_R <- vol_R
results_KDE$volKDE_W <- vol_W
results_KDE[results_KDE$Hybrid=="RW_F2", "volKDE_Hyb"] <- vol_RW2
results_KDE[results_KDE$Hybrid=="WR_F2", "volKDE_Hyb"] <- vol_WR2
## Calculate ratio of the hypervolume of the hybrid class over the mean parental hypervolume
results_KDE[results_KDE$Hybrid=="RW_F2", "volKDEratio"] <- vol_RW2 / ((vol_R + vol_W)/2)
results_KDE[results_KDE$Hybrid=="WR_F2", "volKDEratio"] <- vol_WR2 / ((vol_R + vol_W)/2)

## Count N transgressive individuals from the plots (individuals that fall outside parental 95% kde)
par(mfrow=c(1,2))
plot(kde_R, drawpoints = TRUE, pch = 18, col.pt = treatmentCol[1], cex = 1.5, cont = limT, col = treatmentCol[1], main = "RW F2", xlim = c(1.5,4), ylim = c(1.5,4.5), xlab="Trials to learn association (log)", ylab="Trials to learn reversal (log)")
plot(kde_W, add = TRUE, drawpoints = TRUE, pch = 18, col.pt = treatmentCol[2], cex = 1.5, cont = limT, col = treatmentCol[2])
plot(kde_RW2, add = TRUE, drawpoints = TRUE, pch = 16, col.pt = treatmentCol[5], cex = 1.5, cont = 0, col = treatmentCol[5])

plot(kde_R, drawpoints = TRUE, pch = 18, col.pt = treatmentCol[1], cex = 1.5, cont = limT, col = treatmentCol[1], main = "WR F2", xlim = c(1.5,4), ylim = c(1.5,4.5), xlab="Trials to learn association (log)", ylab=NA)
plot(kde_W, add = TRUE, drawpoints = TRUE, pch = 18, col.pt = treatmentCol[2], cex = 1.5, cont = limT, col = treatmentCol[2])
plot(kde_WR2, add = TRUE, drawpoints = TRUE, pch = 17, col.pt = treatmentCol[6], cex = 1.5, cont = 0, col = treatmentCol[6])

n_transgRW2 <- 0
# >> N = 0 RW F2 individuals that are transgressive
n_transgWR2 <- 0
# >> N = 0 WR F2 individuals that are transgressive

## Save the transgressive individual counts
results_KDE[results_KDE$Hybrid=="RW_F2", "nTransg"] <- n_transgRW2
results_KDE[results_KDE$Hybrid=="WR_F2", "nTransg"] <- n_transgWR2
results_KDE[results_KDE$Hybrid=="RW_F2", "pTransg"] <- ( n_transgRW2 / nrow(RW2_subsetDF) )*100
results_KDE[results_KDE$Hybrid=="WR_F2", "pTransg"] <- ( n_transgWR2 / nrow(WR2_subsetDF) )*100

## CALCULATE PHENOTYPIC MISMATCH & PARENTAL BIAS
## function externally loaded
## ~~~~~
transgression_analysis(data = CognSpace, nameP1 = "R", nameP2 = "W", nameH = "RW_F2", vars = inclVars)
## save output of RW into a new file
transgOutputDF <- transg_outputDF
# clean environ
rm(transg_outputDF)

## Calculate height and distance for WR
transgression_analysis(data = CognSpace, nameP1 = "R", nameP2 = "W", nameH = "WR_F2", vars = inclVars)
## merge output of WR
transgOutputDF <- rbind(transgOutputDF, transg_outputDF)
# clean environ
rm(transg_outputDF)

print(results_KDE)
```

```
##   Hybrid nTransg pTransg volKDEratio volKDE_Hyb volKDE_R volKDE_W
## 1  RW_F2       0       0   0.9699813   2.113112 2.314476 2.042541
## 2  WR_F2       0       0   0.9535623   2.077344 2.314476 2.042541
## 3   SimH      NA      NA          NA         NA 2.314476 2.042541
```

```
print(transgOutputDF)
```

```
##   Hybrid_ID P1_ID P2_ID ParentalBias ParentalMismatch Midpoint_P1P2
## 1     RW_F2     R     W  -0.13082978       0.22166591    0.08710428
## 2     WR_F2     R     W   0.05471729       0.03088551    0.08710428
```

**Figure 5.4.1.** Distribution of individual F2 hybrids and parentals in the cognitive space, with countours of parental 95% KDEs (a,b).

```
## SIMULATE HYBRID PHENOTYPES FROM COMPLETE ADDITIVE INHERITANCE OF PHENOTYPE
## ~~~~~
## Function that randomly selects N parental individuals from each species to generate a sample of N simulated hybrids
#   > data: full dataframe used in the KDE the analyses
#   > P1: string with name of parental species 1
#   > P2: string with name of parental species 2
#   > N: pre-defined sample size, default n = 20 to match sample size of real dataset
#   > vars: name of the columns to use for analyses

hybrid_simulation <- function(N, data, P1 = "R", P2 = "W", vars)
{
  ## subset dataset for target species and target variables
  parental1DF <- subset(data, Species == P1, select = vars)
  parental2DF <- subset(data, Species == P2, select = vars)
  ## reset rownames
  rownames(parental1DF) <- NULL
  rownames(parental2DF) <- NULL
  ## ~~~~~
  ## generate the random parental pairs
  parental1_rands <- as.numeric( sample( rownames(parental1DF), size = N, replace = TRUE ) )
  parental2_rands <- as.numeric( sample( rownames(parental2DF), size = N, replace = TRUE ) )
  ## create empty matrix to save simulated hybrid population
  simHybDF <- matrix(ncol = 2, nrow = N)
  colnames(simHybDF) <- vars
  ## ~~~~~
  ## simulate N hybrids as the linear mean of the parental individuals
  for(iHybrid in 1:N) {
    phenotP1 <- parental1DF[parental1_rands[iHybrid], ]
    phenotP2 <- parental2DF[parental2_rands[iHybrid], ]
    ## generate mean hybrid phenotype
    phenotSimH <- colMeans( rbind(phenotP1, phenotP2) )
    ## save to matrix
    simHybDF[iHybrid, ] <- phenotSimH  }
  return(simHybDF)
}

## Repeat the simulation N times and save all hybrid populations as a list
nSim = 100 # nSim: pre-defined number of simulated runs
simNindivs = 20
## run function
simHybList <- replicate(nSim, hybrid_simulation(N=simNindivs, data=CognSpace, vars=inclVars), simplify=FALSE)

## Calculate KDE of each simulation
## ~~~~~
simHybKDEs <- lapply(simHybList, kde)

## Get mean phenotype of each hybrid population
## ~~~~~
hybMeans <- lapply(simHybList, colMeans)
# extract trials to assoc means
simHybMeans <- unlist( lapply(hybMeans, function(x) x[1]) )
names(simHybMeans) <- NULL
# extract trials to revers means
simHybMeans <- cbind(simHybMeans, unlist( lapply(hybMeans, function(x) x[2]) ) )
rownames(simHybMeans) <- NULL
colnames(simHybMeans) <- inclVars
## data frame with mean phenotypes
simHybMeans <- as.data.frame(simHybMeans)
```

```
## ESTIMATE PHENOTYPIC DISPERSION OF SIMULATED HYBRIDS
## ~~~~~
## Get volume of 95% kde of each simulated population
simHybVols <- unlist( lapply(simHybKDEs, contourSizes, cont=limT, approx=TRUE) )
## save mean volume of simulated hybrid populations
results_KDE[results_KDE$Hybrid == "SimH", "volKDE_Hyb"] <- mean(simHybVols)
## Calculate ratio of the hypervolume of the simulated hybrid population over the mean parental hypervolume
results_KDE[results_KDE$Hybrid=="SimH", "volKDEratio"] <- mean(simHybVols) / ((vol_R + vol_W)/2)

# reshape from list to long format data frame
allSimHPops_1 <- as.data.frame(simHybList)
test <- melt(allSimHPops_1)
rm(allSimHPops_1)
# add simulation ID to each row: every 40 rows is a new simulation
simIDs <- sort( rep(seq(1:nSim), 40) )
test$simPops <- paste0("Sim_", simIDs)
varNames <- c( rep("logTrials_assoc", 20), rep("logTrials_rev", 20) )
test$variable <- rep(varNames, nSim)
## reshape back into two variable columns with the simulation ID as identifyer
allSimHPops <- subset(test, variable == "logTrials_assoc")
allSimHPops <- data.frame("Species" = allSimHPops[, "simPops"], "logTrials_assoc" = allSimHPops[, "value"])
allSimHPops$logTrials_rev <- test[test$variable == "logTrials_rev", "value"]

## Count N transgressive individuals from the plots (individuals that fall outside parental 95% kde)
plot(kde_R, drawpoints = FALSE, cont = limT, col = treatmentCol[1], main = "Simulated hybrids", xlim = c(1.5,4), ylim = c(1.5,4.5), xlab="Trials to learn association (log)", ylab="Trials to learn reversal (log)")
plot(kde_W,  add = TRUE, drawpoints = FALSE, cont = limT, col = treatmentCol[4])
points(x = allSimHPops$logTrials_assoc, y = allSimHPops$logTrials_rev, col = simHCol, pch = 20, cex = 1.2)

nSimTransg <- 0
nSimTot <- 20*nSim
## >>> 0 transgressive individuals out of 2000 simulated hybrids

## save to results table
results_KDE[results_KDE$Hybrid=="SimH", "nTransg"] <- nSimTransg
results_KDE[results_KDE$Hybrid=="SimH", "pTransg"] <- nSimTransg / nSimTot

## CALCULATE PHENOTYPIC MISMATCH & PARENTAL BIAS FOR EACH SIMULATED POP
## function externally loaded
## ~~~~~

## create data frame with parental and simulated hybrid data to feed to function calculating mean phenotype mismatch and bias
CognSpace_simH <- subset(CognSpace, select = c("Species", "logTrials_assoc", "logTrials_rev"))
CognSpace_simH$Species <- as.character(CognSpace_simH$Species)
allSimHPops$Species <- as.character(allSimHPops$Species)
CognSpace_simH <- rbind(CognSpace_simH, allSimHPops)
rownames(CognSpace_simH) <- NULL

## Calculate height and distance for each simulated hybrid population
simPops_ids <- unique(allSimHPops$Species)
for (iSimPop in simPops_ids) {
  ## calculate height and position for each simulated pop
  transgression_analysis(data = CognSpace_simH, nameP1 = "R", nameP2 = "W", nameH = iSimPop, vars = inclVars)
  ## merge output to main data frame
  transgOutputDF <- rbind(transgOutputDF, transg_outputDF)
  rm(transg_outputDF)
}
## print on screen mean & SD of all simulated hybrid pops:
## ~~~~~
## phenotypic dispersion
mean(simHybVols)
```

```
## [1] 1.170715
```

```
sd(simHybVols)
```

```
## [1] 0.2784975
```

```
## proportion of transgressive simulated hybrids
mean(nSimTransg)
```

```
## [1] 0
```

```
sd(nSimTransg / nSimTot)
```

```
## [1] NA
```

```
## parental bias 
mean(transgOutputDF[3:nrow(transgOutputDF), "ParentalBias"])
```

```
## [1] -0.003077614
```

```
sd(transgOutputDF[3:nrow(transgOutputDF), "ParentalBias"])
```

```
## [1] 0.06709406
```

```
## phenotypic mismatch
mean(transgOutputDF[3:nrow(transgOutputDF), "ParentalMismatch"])
```

```
## [1] 0.05561416
```

```
sd(transgOutputDF[3:nrow(transgOutputDF), "ParentalMismatch"])
```

```
## [1] 0.04008136
```

**Figure 5.4.2.** Distribution of individual simulated hybrids in the cognitive space, with countours of parental 95% KDEs (c).

---

#### Plots phenotypic dispersion, mismatch, and parental bias

```
## Hypervolume (phenotypic dispersion)
## ~~~~~
# RW
plot(x = 1, y = results_KDE$volKDE_Hyb[1], col = 1, bg = treatmentCol[5], pch = 21, cex = 1.3, 
     ylim = c(0, 2.6), xlim=c(0.9,1.1), ylab = "Hypervolume", xlab = NA, xaxt='n')
# simulated pops
points(x = jitter(rep(1, nSim)), y = simHybVols, col = rgb(0.7, 0.7, 0.7, alpha=0.5), pch = 16, cex = 1)
# WR
points(x = 1.015, y = results_KDE$volKDE_Hyb[2], col = 1, bg = treatmentCol[6], pch = 24, cex = 1.3)
# R
points(x = 1, y = results_KDE$volKDE_R[1], col = 1, bg = treatmentCol[1], pch = 23, cex = 1.3)
# W
points(x = 0.99, y = results_KDE$volKDE_W[1], col = 1, bg = treatmentCol[2], pch = 23, cex = 1.3)

## Parental bias
## ~~~~~
# simulated pops
plot(x = jitter(rep(1, nSim)), y = transgOutputDF$ParentalBias[3:nrow(transgOutputDF)], col = rgb(0.7, 0.7, 0.7, alpha=0.5), pch = 16, cex = 1, ylim = c(-0.4, 0.4), xlim = c(0.9, 1.1), ylab = "Parental bias", xlab = NA, xaxt='n')
# RW
points(x = 1, y = transgOutputDF$ParentalBias[1], col = 1, bg = treatmentCol[5], pch = 21, cex = 1.3)
# WR
points(x = 1, y = transgOutputDF$ParentalBias[2], col = 1, bg = treatmentCol[6], pch = 24, cex = 1.3)
# text
text(0.96, -0.42, "closer to W", cex=0.7, col="grey50", pos=3)
text(0.96, 0.45, "closer to R", cex=0.7, col="grey50", pos=1)

# Phenotypic mismatch
## ~~~~~
# simulated pops
plot(x = jitter(rep(1, nSim)), y = transgOutputDF$ParentalMismatch[3:nrow(transgOutputDF)], col = rgb(0.7, 0.7, 0.7, alpha=0.5), pch = 16, cex = 1, ylim = c(0, 0.4), xlim = c(0.9, 1.1), ylab = "Phenotypic mismatch", xlab = NA, xaxt='n')
# RW
points(x = 1, y = transgOutputDF$ParentalMismatch[1], col = 1, bg = treatmentCol[5], pch = 21, cex = 1.3)
# WR
points(x = 1, y = transgOutputDF$ParentalMismatch[2], col = 1, bg = treatmentCol[6], pch = 24, cex = 1.3)
```

**Figure 5.4.3.** Phenotypic dispersion (a), parental bias (b), and phenotypic mismatch (c) of observed and simulated F2 hybrid cognitive phenotypes in relation to observed parental cognitive phenotypes.

---

## 5.5 Cognitive space of hybrid males

Due to the small number of males that succeeded in the reversal learning task, we could not run any analyses on male cognitive performance.

div< class = “model”>

```
## sample size for the comparison
length(complete.cases(learnersRLm) == TRUE)
```

```
## [1] 16
```

```
ggplot(data = LCMale, aes(x = TrialsLC_AL, y = TrialsLC_RL, colour = Species)) +
  geom_point(aes(shape = Species), size = 3, stroke = 1.3, position = position_jitter(w = 0.15, h = 0.15)) +
  scale_shape_manual(values = c(1,2, 16, 17, 16, 17)) +
  scale_color_manual(values = treatmentCol) +
  scale_fill_manual(values = treatmentCol) +
  scale_y_continuous(breaks = seq(0,60,10), limits = c(2,60), expand = expansion(mult = c(0, 0))) +
  scale_x_continuous(breaks = seq(0,40,10), limits = c(2,40), expand = expansion(mult = c(0, 0))) +
  ylab("Trials to succeed reversal") +
  xlab("Trials to succeed association") +
  theme_classic(base_size = 11) + theme(
    panel.background = element_blank(),
    panel.border = element_blank(),
    axis.text = element_text(colour = "black"),
    axis.title.x = element_text(margin = unit(c(3, 0, 0, 0), "mm")),
    axis.title.y = element_text(margin = unit(c(0, 3, 0, 0), "mm")) )
```
